# In vivo base editing extends lifespan of a humanized mouse model of prion disease

**DOI:** 10.1038/s41591-024-03466-w

**Published:** 2025-01-14

**Authors:** Meirui An, Jessie R. Davis, Jonathan M. Levy, Fiona E. Serack, John W. Harvey, Pamela P. Brauer, Catherine P. Pirtle, Kiara N. Berríos, Gregory A. Newby, Wei-Hsi Yeh, Nikita Kamath, Meredith Mortberg, Yuan Lian, Michael Howard, Kendrick DeSouza-Lenz, Kenia Guzman, Aaron Thai, Samantha Graffam, Vanessa Laversenne, Alissa A. Coffey, Jeannine Frei, Sarah E. Pierce, Jiri G. Safar, Benjamin E. Deverman, Eric Vallabh Minikel, Sonia M. Vallabh, David R. Liu

**Affiliations:** 1 Merkin Institute of Transformative Technologies in Healthcare, Broad Institute of MIT and Harvard, Cambridge, MA USA; 2 Department of Chemistry and Chemical Biology, Harvard University, Cambridge, MA USA; 3 Howard Hughes Medical Institute, Harvard University, Cambridge, MA USA; 4 Stanley Center for Psychiatric Research, Broad Institute of MIT and Harvard, Cambridge, MA USA; 5 Comparative Medicine, Broad Institute of MIT and Harvard, Cambridge, MA USA; 6 Case Western Reserve University, Cleveland, OH USA; 7 McCance Center for Brain Health and Department of Neurology, Massachusetts General Hospital, Boston, MA USA; 8 Department of Neurology, Harvard Medical School, Boston, MA USA; 9 Prion Alliance, Cambridge, MA USA

**Keywords:** Targeted gene repair, Prion diseases

## Abstract

Prion disease is a fatal neurodegenerative disease caused by the misfolding of prion protein (PrP) encoded by the *PRNP* gene. While there is currently no cure for the disease, depleting PrP in the brain is an established strategy to prevent or stall templated misfolding of PrP. Here we developed in vivo cytosine and adenine base strategies delivered by adeno-associated viruses to permanently modify the *PRNP* locus to achieve PrP knockdown in the mouse brain. Systemic injection of dual-adeno-associated virus PHP.eB encoding BE3.9max and single guide RNA installing *PRNP* R37X resulted in 37% average installation of the desired edit, 50% reduction of PrP in the mouse brain and 52% extension of lifespan in transgenic human *PRNP* mice inoculated with pathogenic human prion isolates representing the most common sporadic and genetic subtypes of prion disease. We further engineered base editing systems to achieve improved in vivo potency and reduced base editor expression in nontargeting tissues, resulting in 63% average PrP reduction in the mouse brain from a 6.7-fold lower viral dose, with no detected off-target editing of anticipated clinical significance observed in either human cells or mouse tissues. These findings support the potential of in vivo base editing as one-time treatment for prion disease.

## Abstract (web summary)

An in vivo base editing approach targeting the *PRNP* gene led to a 52% increase in the lifespan of mouse models inoculated with the most common sporadic and genetic types of human pathogenic prion isolate.

## Main

The misfolding and accumulation of PrP in neurons causes prion disease, a currently incurable and always fatal neurological disease which includes subtypes such as Creutzfeldt–Jakob disease (CJD), Gerstmann–Straussler–Scheinker disease and fatal familial insomnia^1^. A PrP-lowering antisense oligonucleotide (ASO)^2^ is currently in a Phase I clinical trial, but no approved therapies are currently available for human use. A therapeutic to halt or delay disease progression is urgently needed.

Misfolded PrP causes prion disease via a toxic gain of function^1^, with 85% of cases caused by a spontaneous misfolding event of PrP, 15% caused by protein-coding mutations in the *PRNP* gene and <1% caused by infection. Removal of cellular PrP to eliminate substrates for misfolded prion aggregation is a promising therapeutic strategy for prion disease. PrP has a signaling function related to myelin maintenance on peripheral nerves^3^, but reduction or elimination of PrP appears to be compatible with healthy life^3,4^. Heterozygous *Prnp* knockout mice show enhanced resistance to prion disease, and homozygous *Prnp* knockout mice are completely resistant to prion disease^5^. While no homozygous *PRNP*-null humans have been observed, heterozygous null humans appear healthy, and occur at a frequency of ~1/18,000 healthy individuals^4^.

While ASO-mediated knockdown of PrP holds promise as a potential disease-modifying therapy for prion disease, central nervous system (CNS)-targeting ASOs currently suffer from limited potency and biodistribution to deep brain regions^6^, the need for repeated intrathecal dosing and an unknown long-term tolerability profile. As an alternative and perhaps synergistic strategy to ASOs, genome editing of *PRNP* could offer a one-time treatment with biodistribution and safety profiles distinct from ASOs. Nuclease-mediated disruption of *PRNP* could theoretically be used to knockdown or inactivate PrP, but the uncontrolled mixture of deletions, insertions and other mutated byproducts poses the risk of creating new pathogenic PrP variants. A targeted epigenetic approach was recently used in mice to durably lower mouse *Prnp* expression by promoter methylation, raising the possibility of applying this approach to human *PRNP*^7^.

Base editing has been shown to mediate precise and permanent knockout of genes via installation of an early stop codon^8–10^ or disruption of a start codon^11^ with minimal indel byproducts^12,13^, offering a more precise method for PrP knockdown. Furthermore, a base editing strategy that disrupts *PRNP* expression would be largely independent of prion etiology or nature of a patient’s mutation, greatly expanding the patient population that would benefit from such a therapy. These considerations led us to pursue a precise knockdown strategy of PrP in the humanized prion mouse model using in vivo base editing.

## Results

### CBE-mediated premature stop codon installation in *PRNP*

**Fig. 1:**
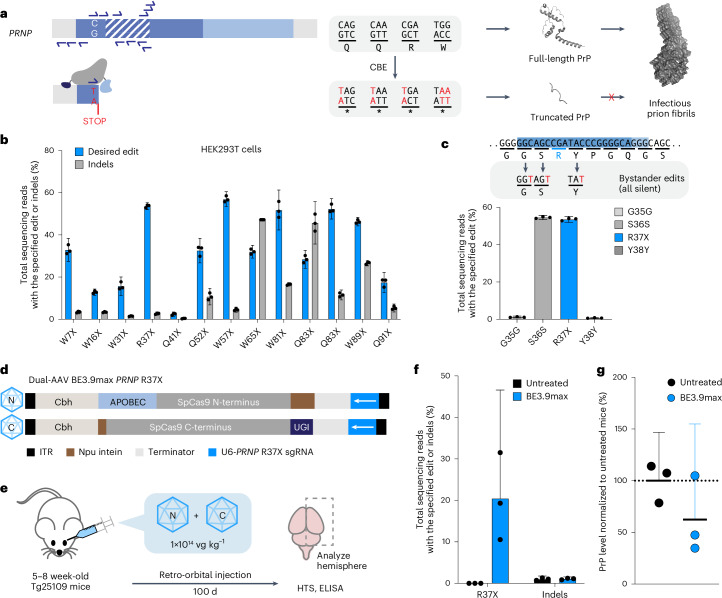
Development of initial base editing strategies to install stop codon in *PRNP* locus. **a**, Schematic of the CBE-mediated stop codon installation as a strategy to knockdown cellular PrP. The *PRNP* locus consists of N-terminal (dark blue, amino acids 1–144) and C-terminal (light blue, amino acids 145–253) domains. The signal peptide (gray, amino acids 1–22), octapeptide repeat (OPR) region (dashed box, amino acids 51–90) and GPI signal (gray, amino acids 231–253) are highlighted. CBE may convert CAG (Gln), CAA (Gln), CGA (Arg) or TGG (Trp) codons to a stop codon. sgRNA spacers that install stop codons in *PRNP* evaluated in this study are shown as half-arrows. Truncated PrP no longer templates fibril formation. **b**, Frequency of the desired stop codon installation or indel formation from candidate sgRNAs using BE4max via plasmid transfection of HEK293T cells. **c**, Editing efficiency at bystander positions with BE4max and *PRNP* R37X sgRNA via plasmid transfection of HEK293T cells. Three silent mutations (G35G, S36S and Y38Y) are possible due to bystander editing from cytosine base editing. **d**, Schematic of dual-AAV PHP.eB BE3.9max with *PRNP* R37X sgRNA. The N-terminal AAV encodes a Cbh promoter, APOBEC deaminase domain and amino acids 1–572 of SpCas9 fused to NpuN intein. The C-terminal AAV encodes a Cbh promoter, NpuC intein, amino acids 573–1367 of SpCas9 and one copy of the UGI domain. Both AAVs contain a U6 Pol III cassette expressing the *PRNP* R37-targeting sgRNA. **e**, Experimental design for initial assessment of the effect of *PRNP* base editing on PrP levels. The 5–8-week-old Tg25109 mice were treated retro-orbitally with dual-AAV PHP.eB BE3.9max for installation of *PRNP* R37X at a dose of 1 × 10^14^ vg kg^−1^. Brain was harvested 100 d post injection to assess editing efficiency via HTS and PrP protein reduction via ELISA. **f**, Frequency of R37X installation in untreated (*n* = 3) and dual-AAV PHP.eB BE3.9max-treated mice (*n* = 3). **g**, PrP levels in dual-AAV PHP.eB BE3.9max-treated mice (*n* = 3) in the bulk brain hemisphere normalized to those of untreated mice (*n* = 3). Dots represent individual biological replicates (*n* = 3) and data are presented as mean ± 95% CI.

Cytosine base editors (CBEs) convert C•G to T•A^12^, enabling the conversion of Arg, Gln or Trp codons into stop codons^8–10^ (Fig. 1a). We designed 13 single guide RNAs (sgRNAs) with SpCas9-compatible NGG protospacer-adjacent motifs that would position the base editing window to convert a codon to a premature stop codon. We focused on the region of *PRNP* corresponding to the N terminus of PrP (amino acids 1–131), as some C-terminal truncating variants are known to exhibit pathogenic gain-of-function^14,15^. We transfected HEK293T cells with plasmids encoding the designed sgRNA and BE4max^16^, a CBE containing an APOBEC cytidine deaminase domain, SpCas9 nickase (D10A) and two copies of the uracil DNA glycosylase inhibitor (UGI). BE4max was a state-of-the-art CBE at the time this study began.

Assessment of editing efficiencies by high-throughput sequencing (HTS) revealed efficient base editing targeting Trp 57 (W57X), Arg 37 (R37X), Gln 83 (Q83X) and Trp 81 (W81X), with average editing efficiencies of 57%, 54%, 52% and 52%, respectively (Fig. 1b). We chose to advance the R37X strategy because the heterozygous *PRNP* R37X variant has been observed twice in healthy humans^4^, suggesting that this truncation variant is not pathogenic. Installation of *PRNP* R37X with BE4max resulted in three silent bystander edits: at Gly 35 (GGC to GGT), Ser 36 (AGC to AGT) and Tyr 38 (TAC to TAT) with average efficiencies of 1.2%, 55% and 0.68%, respectively (Fig. 1c), with indel levels of 2.7% (Fig. 1b).

**Extended Data Fig. 1:**
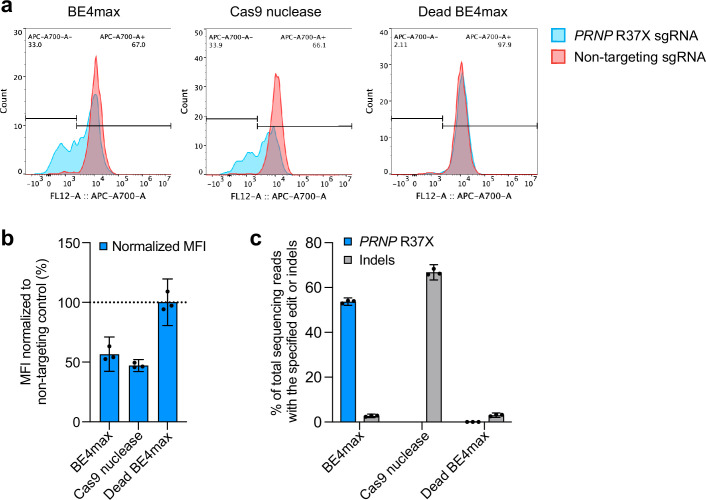
*In vitro* validation of PrP reduction with CBE-mediated PRNP R37X installation. **a**, Representative flow cytometry gating plot showing the fluorescence signal in HEK293T cells treated with BE4max, Cas9 nuclease or dead (deaminase-inactive) BE4max editor, with either *PRNP* R37X sgRNA (blue) or non-targeting sgRNA (red). Six days after plasmid transfection, cells were incubated with a fluorescently conjugated 6D11 antibody that binds PrP and were analyzed by flow cytometry. **b**, Mean fluorescence intensity (MFI) of the HEK293T cells after staining with 6D11 antibodies. MFI are plotted after normalizing the MFI of cells treated with *PRNP* R37X sgRNAs to MFI of cells treated with non-targeting sgRNAs. **c**, Frequency of the *PRNP* R37X edit and indels in HEK293T cells after plasmid transfection with BE4max, Cas9 nuclease or dead BE4max, with *PRNP* R37-targeting sgRNA. Dots represent individual biological replicates (n=3) and data are presented as mean values +/- 95% CI.

To assess the reduction of cellular PrP after base editor treatment, we incubated the transfected cells with a fluorophore-conjugated anti-PrP antibody and performed flow cytometry analysis. We observed 43% average reduction in mean fluorescence intensity in cells treated with BE4max and *PRNP* R37X sgRNAs compared with cells treated with a control *BCL11A*-targeting sgRNA (Extended Data Fig. 1a,b). This reduction was similar to the level of PrP knockdown achieved with Cas9 nuclease (53% average reduction) but with substantially increased editing product purity (Extended Data Fig. 1c). Given the high on-target editing efficiency without additional nonsynonymous bystander edits, concomitant protein knockdown and the presence of R37X polymorphism in healthy humans, we advanced the *PRNP* R37X strategy for in vivo studies in mice.

### Assessment of cellular PrP reduction in vivo

To deliver the base editor and sgRNA into animals, we used the previously reported v5 dual-adeno-associated virus (AAV) BE architecture packaging two halves of intein^17^-fused CBE3.9max (ref. ^18^), a CBE that contains one copy of UGI domain to accommodate the base editor within the packaging size limit of AAV. Each AAV includes a U6 promoter that expresses sgRNA encoding *PRNP* R37X (Fig. 1d). Upon co-transduction of both vectors, functional base editor is reconstituted^18^. We packaged the resulting AAVs using the PHP.eB capsid^19^, which can transduce neurons either by direct injection into the CNS, or by systemic injection via efficient blood–brain barrier crossing in compatible mouse strains^20^.

**Extended Data Fig. 2:**
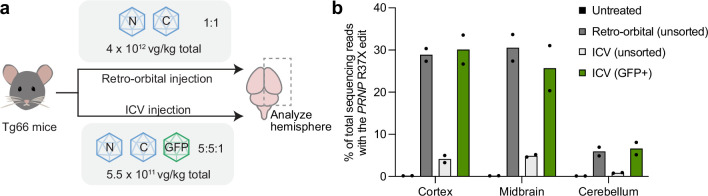
Optimization of *in vivo* administration route. **a**, Experimental design for assessment of *in vivo* administration route. 4-week-old Tg66 mice were treated with dual-AAV PHP.eB BE3.9max for installation of *PRNP* R37X by systemic administration through retro-orbital injection or direct CNS administration through intracerebroventricular (ICV) stereotaxic injection. Mice treated with retro-orbital injection received a total of 4x10^12^ vg/kg AAV (2x10^12^ vg/kg each for N- and C-terminal AAV) (n=2). Mice treated with ICV injection received a total of 5.5x10^11^ vg/kg AAV (2.5x10^11^ vg/kg each for N- and C-terminal AAV and 5.0x10^10^ vg/kg for AAV encoding EGFP fused to nuclear membrane-localized Klarsicht/ANC-1/Syne-1 homology (KASH) domain (EGFP:KASH)) (n=2). Mouse brains were harvested five weeks after treatment. GFP-positive nuclei were sorted from ICV-injected samples via FACS to enrich for AAV-transduced cells. Genomic DNA extracted from the brain tissues were analyzed for editing efficiency. **b**, Frequency of the desired R37X edit in the bulk brain hemisphere of mice untreated (n=2) or treated with dual-AAV PHP.eB BE3.9max by retro-orbital injection (n=2), or by ICV injection (n=2). Dots represent individual biological replicates and bar graphs represent mean values.

Pilot experiments comparing direct CNS administration by intracerebroventricular injection with systemic administration by retro-orbital injection in mice expressing a human *PRNP* transgene^21^ showed that systemic administration can achieve potent editing in the brain (Extended Data Fig. 2a,b). Further evaluation of the base editing strategy in vivo was performed in humanized Tg25109 mice^22^ that harbor three copies of wild-type human *PRNP* and produce human PrP at approximately wild-type levels. To assess whether *PRNP* R37X installation could reduce PrP protein levels in vivo, we systemically administered dual-AAV PHP.eB encoding BE3.9max and *PRNP* R37X-installing sgRNA at a total dose of 1 × 10^14^ total viral genomes (vg) kg^−1^ (5 × 10^13^ vg kg^−1^ each of N- and C-terminal BE-AAVs) (Fig. 1e). Mouse brains were harvested 100 d post injection, and editing efficiency and PrP levels in bulk brain hemispheres were measured by HTS and enzyme-linked immunosorbent assay (ELISA), respectively. We observed 20% of total alleles contained the desired *PRNP* R37X edit (Fig. 1f), accompanied by a corresponding 31% decrease in PrP levels (Fig. 1g).

### In vivo base editing protects from human prion challenge

Since several small-molecule therapeutics that were effective in wild-type mice infected with murine pathogenic prion isolates proved to be ineffective in humanized mice infected with human pathogenic prion isolates^23–26^, we performed a human pathogenic prion inoculate challenge study using the humanized Tg25109 mice to better assess the therapeutic relevance of BE-AAV for treating human prion disease.

We dosed mice with BE-AAVs 1 week before pathogenic prion inoculation for the following reasons: first, the age-associated penetrance of prion disease mutation provides an opportunity for prophylaxis in pre-symptomatic *PRNP* mutation carriers, a large majority of whom are negative for prion seeding activity^27^. Second, previous studies of PrP-targeting ASO treatment in mice showed comparable therapeutic benefits at any time point from prophylactic dosing up to early stage of disease onset, suggesting that the findings from the prophylactic paradigm should be applicable to treatment at later timepoints up to the advanced neuropathology that precedes first symptoms^2^. Third, we aimed to reduce biosafety risk by dosing AAVs before inoculation, minimizing animal handling after human prion inoculation.

**Fig. 2:**
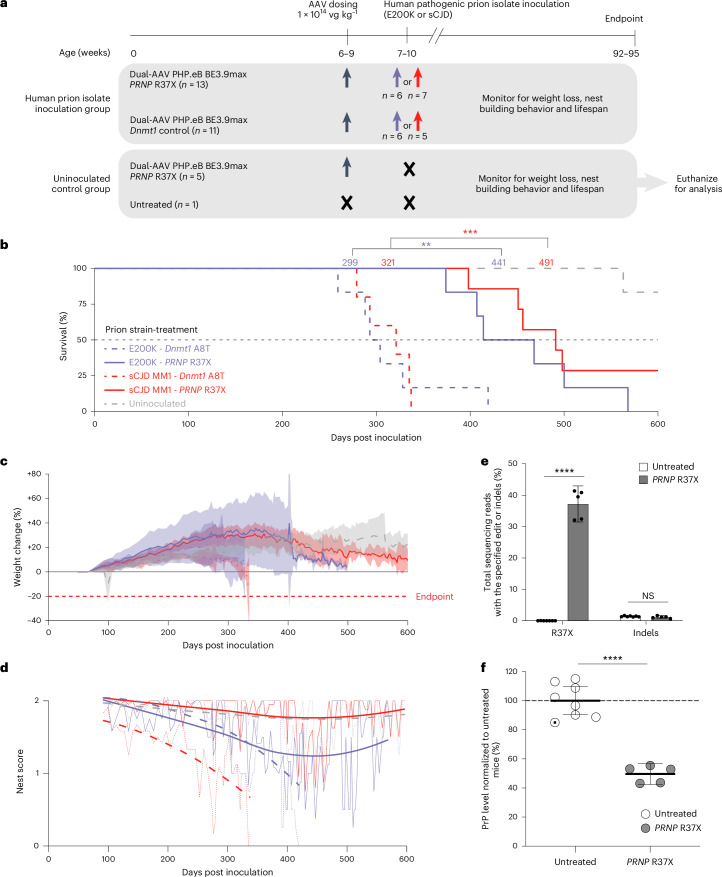
In vivo base editing provides protection from pathogenic human prion challenge. **a**, Design of the human pathogenic prion challenge study. Tg25109 mice were divided into two cohorts: a human prion isolate inoculation group and an uninoculated control group. Among the human prion isolate inoculation group, *n* = 13 received dual-AAV PHP.eB BE3.9max *PRNP* R37X treatment and *n* = 11 received dual-AAV PHP.eB BE3.9max *Dnmt1* control treatment. Among the uninoculated control group, *n* = 5 received dual-AAV PHP.eB BE3.9max *PRNP* R37X treatment and *n* = 1 remained untreated. Mice were treated with AAV at total dose of 1 × 10^14^ vg kg^−1^ at age 6–9 weeks. At 1 week after AAV treatment, mice were inoculated with either E200K or sCJD prion isolates. After prion inoculation, mice were monitored for weight loss, nest-building behavior and lifespan. Study endpoint was 600 d post prion isolate inoculation (92–95 weeks of age). The uninoculated control group was euthanized to harvest brain hemispheres for analysis via HTS and PrP ELISA. **b**, Kaplan–Meier curve of Tg25109 mice inoculated with either the E200K (purple) or sCJD MM1 pathogenic human prion isolate (red). Median survival from each treatment condition is marked (*P* = 4 × 10^−4^ for sCJD-inoculated cohort; *P* = 0.01 for E200K cohort; *P* = 2 × 10^−6^ combined). **c**,**d**, Body weight (**c**) (lines represent mean and shaded areas represent 95% CI) for all timepoints with ≥2 animals surviving, and nest-building score (**d**) (fitted to the locally estimated scatterplot smoothing (LOESS) model) of Tg25109 mice in the human prion challenge study. **e**,**f**, Frequency of the desired R37X edit (**e**) (*P* < 0.0001) and indels, and PrP protein level (**f**) (*P* < 0.0001) in the bulk brain hemisphere of mice from the uninoculated control group treated with dual-AAV PHP.eB BE3.9max with *PRNP* R37X sgRNA (*n* = 5), and in untreated mice from the uninoculated control group (*n* = 1, marked as a white circle with a black dot) or from additional untreated adult Tg25109 mice (*n* = 7, marked as white circles). Dots represent individual biological replicates and data are presented as mean ± 95% CI. Significance was calculated by two-tailed Student’s *t*-test; **P ≤ 0.01; ***P ≤ 0.001; ****P ≤ 0.0001. NS, not significant.

We injected 6–9-week-old Tg25109 mice retro-orbitally with 1 × 10^14^ vg kg^−1^ dual-AAV PHP.eB BE3.9max with either an sgRNA installing *PRNP* R37X (*n* = 21) as the treatment group, or an sgRNA installing *Dnmt1* A8T as a control group (*n* = 16); the *Dnmt1* edit lacks any known association with prion biology nor is it expected to impact phenotype^18^ (Fig. 2a). After AAV injection but before inoculation with prion isolates, three mice in the *PRNP* R37X treatment group and five mice from the *Dnmt1* A8T control group reached predefined euthanasia criteria and were excluded from the study. At 1 week after AAV treatment, of the remaining AAV-treated mice, *n* = 13 mice in the *PRNP* R37X treatment group and *n* = 11 in the *Dnmt1* A8T control group were randomly assigned into cohorts and received stereotaxic inoculation of either one of two clinical pathogenic human prion isolates. The clinical pathogenic prion isolates were sCJD MM1, the most common form of sporadic prion disease^28^, and E200K, the most common mutation in genetic prion disease^29^. Due to biosafety considerations, brain tissue was not retrieved from the prion-inoculated cohorts. Therefore, we kept a small cohort (*n* = 5/18) that received the BE3.9max *PRNP* R37X treatment, but no prion challenge, to facilitate analysis of editing and protein knockdown at the study endpoint.

All cohorts were subsequently monitored for weight, nest-building behavior and signs of neurological decline. By the endpoint of the study at 600 days post prion isolate inoculation (dpi), the average lifespan of the human pathogenic prion-inoculated mice was substantially extended among the *PRNP* R37X-installing base editor-treated mice compared with control mice across both prion isolates (Fig. 2b). In the sCJD MM1 prion-inoculated cohort, BE3.9max *PRNP* R37X-treated animals outlived controls by ≥59% (499 ± 76 versus 313 ± 26 dpi, *n* = 7 versus 5, counting two animals alive at end of study as 600 dpi; *P* = 4 × 10^−4^), with two treated animals alive at the study endpoint while none of the control animals reached study endpoint. In the E200K prion-inoculated cohort, BE3.9max *PRNP* R37X-treated animals outlived controls by 44% (455 ± 71 versus 315 ± 56 dpi, *n* = 6 versus 6; *P* = 0.01). Combined, *n* = 13 treated animals outlived *n* = 11 controls by 52% (*P* = 2 × 10^−6^). Furthermore, steady weight gains were observed in treated mice while the control mice showed sharp decline in body weight (Fig. 2c and Supplementary Table 1). No declines in nest-building behaviors were observed in the sCJD MM1-inoculated mice treated with *PRNP* R37X condition, in contrast to the control mice that showed gradually declining nest scores (Fig. 2d and Supplementary Table 2). These results indicate that the treatment led to extended healthspan, in addition to extended survival.

At 600 dpi, the uninoculated *PRNP* R37X cohort was harvested for the assessment of editing efficiency and PrP protein levels. We observed 37% installation of *PRNP* R37X in whole brain hemispheres in the treated mice (Fig. 2e), and 42% reduction in PrP compared with a single in-study age-matched control mouse or 50% compared with a group of *n* = 7 adult Tg25109 mice (Fig. 2f). Robust PrP reduction observed in the mouse brains supports the significant lifespan extension observed in the prion-inoculated BE3.9max *PRNP* R37X treatment groups.

### Optimization of base editing strategies for improved potency

High doses of AAV are associated with clinical side effects^30,31^, consistent with our observation of toxicity in the mice treated with base editor-expressing AAV at 1 × 10^14^ vg kg^−1^. To advance a BE-AAV strategy towards potential therapeutic application, we sought to optimize base editing strategies to yield similar or better PrP reduction at lower doses of AAV.

**Fig. 3:**
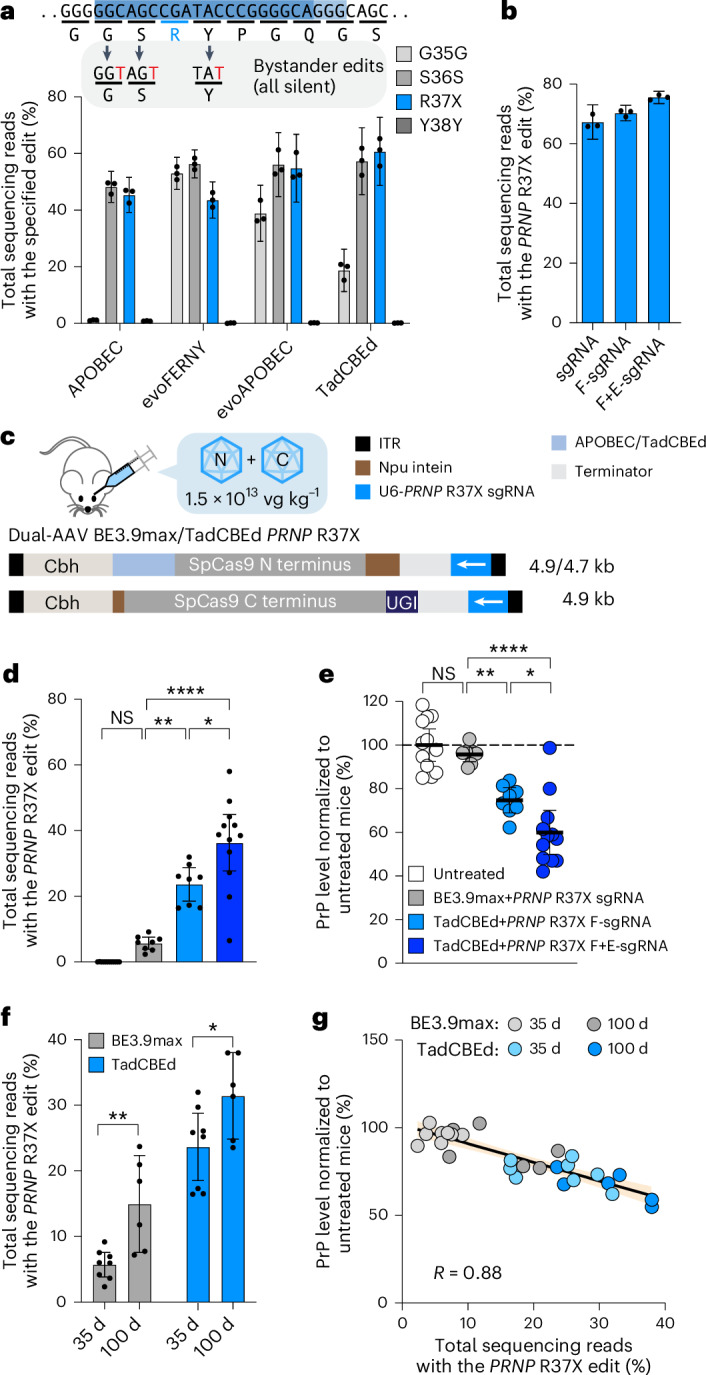
Optimization of CBE strategy for improved potency. **a**, Frequency of R37X installation and bystander editing in HEK293T cells transfected with the specified CBEs and *PRNP* R37X sgRNA. **b**, Frequency of R37X installation in HEK293T cells transfected with TadCBEd and *PRNP* R37X sgRNAs with the specified scaffold modifications: canonical (sgRNA), U-to-A flip (F-sgRNA) or U-to-A flip+5-bp stem extension (F+E-sgRNA). **c**, Schematic of dual-AAV PHP.eB BE3.9max or TadCBEd with *PRNP* R37X sgRNA. The 5–8-week-old Tg25109 mice were treated retro-orbitally with AAVs at 1.5 × 10^13^ vg kg^−1^. **d**,**e**, Frequency of R37X installation (**d**), and PrP level in bulk brain hemisphere (**e**) of Tg25109 mice untreated (*n* = 12) or treated with dual-AAV PHP.eB packaging BE3.9max *PRNP* R37X sgRNA (*n* = 8), TadCBEd *PRNP* R37X sgRNA (*n* = 8) or TadCBEd *PRNP* R37X F+E-sgRNA (*n* = 12) at a total dose of 1.5 × 10^13^ vg kg^−1^ and harvested 35 d post injection. In **d**, untreated versus BE3.9max, *P* = 0.78; BE3.9max versus TadCBEd, *P* = 0.0012; TadCBEd versus TadCBEd with F+E-sgRNA, *P* = 0.030; BE3.9max versus TadCBEd with F+E-sgRNA, *P* < 0.0001. In **e**, untreated versus BE3.9max, *P* > 0.99; BE3.9max versus TadCBEd, *P* = 0.0031; TadCBEd versus TadCBEd with F+E-sgRNA, *P* = 0.045; BE3.9max versus TadCBEd with F+E-sgRNA, *P* < 0.0001. **f**, Frequency of R37X installation in Tg25109 mice 35 d (*n* = 8) versus 100 d (*n* = 6) post treatment with BE3.9max (*P* = 0.0042) or TadCBEd (*P* = 0.038). **g**, Scatter plot showing the relationship between R37X editing frequencies and PrP protein levels in brain tissues of Tg25109 mice treated with BE3.9max or TadCBEd, harvested at 35 d (*n* = 8) or 100 d (*n* = 6). Linear regression is shown with solid line and shaded area represents 95% CI. Dots represent individual biological replicates (*n* = 3 unless noted otherwise) and data are presented as mean ± 95% CI. Significance in **d** and **e** was calculated by two-way analysis of variance (ANOVA) test with Bonferroni correction; NS, *P* > 0.12; *P ≤ 0.033; **P ≤ 0.002; ****P ≤ 0.0001. Significance in **f** was calculated by two-tailed Student’s *t*-test; NS, *P* > 0.05; *P ≤ 0.05; **P ≤ 0.01. kb, kilobases.

We tested three enhanced SpCas9-based CBEs via plasmid transfection in HEK293T cells for installation of *PRNP* R37X. Recently developed TadCBEd^32^ resulted in the highest on-target editing efficiency (61% average editing), with all observed bystander edits creating only silent mutations (Fig. 3a). Optimization of sgRNA to adopt flip-and-extend scaffold^33^ (hereafter referred simply as F+E-sgRNA) further improved editing efficiency in HEK293T cells to 76% (Fig. 3b).

Next, we treated Tg25109 mice at a reduced dose of 1.5 × 10^13^ vg kg^−1^ total (7.5 × 10^12^ vg kg^−1^ each of N- and C-terminal BE-AAVs) and harvested mouse brains 5 weeks after for analysis (Fig. 3c). TadCBEd significantly improved editing efficiency at this lower tested dose over BE3.9max (24% versus 5.7%; *P* = 0.001) (Fig. 3d). Using the F+E-sgRNA scaffold further improved the editing efficiency compared with the sgRNA with the canonical scaffold (36% versus 24%; *P* = 0.03) (Fig. 3d). Improvements in PrP knockdown were observed, from 4.2% PrP reduction with dual-AAV PHP.eB BE3.9max treatment, to 25% PrP reduction with the improved editor (dual-AAV PHP.eB TadCBEd; *P* = 0.003), to 43% PrP reduction with the optimized sgRNA (dual-AAV PHP.eB TadCBEd with F+E-sgRNA; *P* < 0.0001 compared with dual-AAV PHP.eB BE3.9max and *P* = 0.05 compared with dual-AAV PHP.eB TadCBEd with the canonical sgRNA). Analysis of mice harvested 100 d after treatment showed an increase in editing efficiency over time (5.7% at 35 d versus 15% at 100 d for BE3.9max; *P* = 0.004, and 24% at 35 d versus 31% at 100 d for TadCBEd; *P* = 0.04) (Fig. 3f) and corresponding reduction of PrP levels (Fig. 3g), suggesting that the editing is not complete at the 35-d time point and the efficacy of treatment may further increase over time. Importantly, none of the mice treated with this lower AAV dose showed notable weight loss or signs of distress (Supplementary Table 3), suggesting that this lower dose of BE-AAV is well tolerated and the previously observed toxicity indeed arose at least in part from the high dose of BE-AAVs.

**Extended Data Fig. 3:**
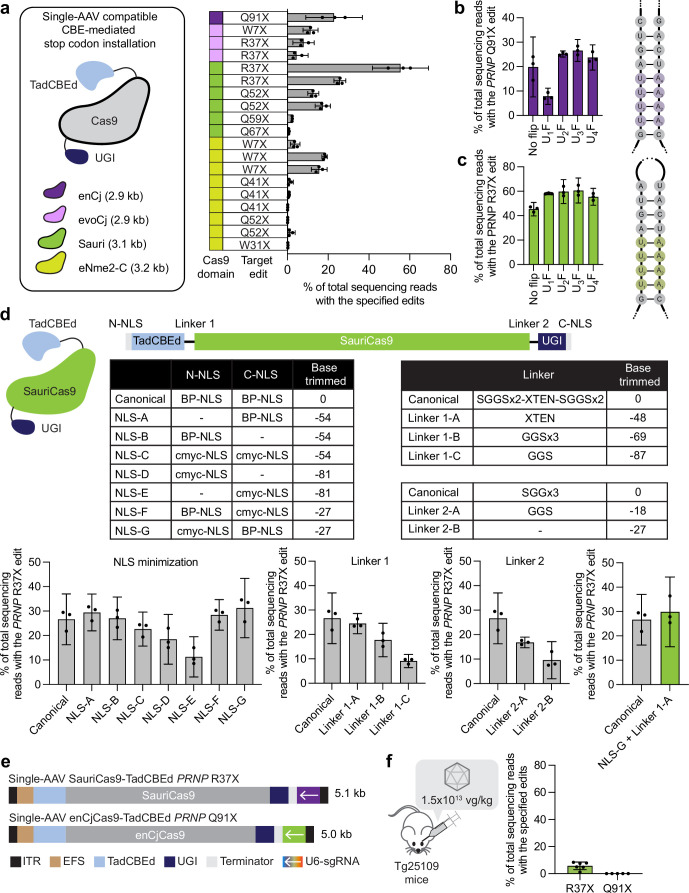
Development of single-AAV CBE strategies. **a**, Schematic of single-AAV compatible CBE-mediated stop codon installation strategy and frequency of the desired stop codon installation in HEK293T cells transfected with size-minimized CBEs and corresponding sgRNAs. Four size-minimized Cas9 domain (enCjCas9, evoCjCas9, SauriCas9, and eNme2-Cas9) were fused to TadCBEd and a UGI domain to generate size-minimized CBEs. **b** and **c**, Frequency of the desired Q91X edit (**b**) and R37X edit (**c**) in HEK293T cells transfected with the base editors and the sgRNAs with the specified modifications. Positions of the poly-U stretch in the enCjCas9 sgRNA scaffold (c) and SauriCas9 sgRNA scaffold (d) are highlighted. ‘No flip’ refers to sgRNA with canonical scaffold; U_n_F refers to sgRNA with the U_n_-A_n_ position flipped to A_n_-U_n_. **d**, Schematic of SauriCas9-TadCBEd, composed of N-terminal NLS (N-NLS), TadCBEd domain, linker 1, SauiCas9 domain, linker 2, UGI domain, and C-terminal NLS (C-NLS). Frequency of the desired R37X edit in HEK293T cells transfected with the SauriCas9-TadCBEd containing the specified modification and the *PRNP* R37X F-sgRNA. **e**, Schematic of single-AAV PHP.eB SauriCas9-TadCBEd with *PRNP* R37X F-sgRNA and single-AAV PHP.eB enCjCas9-TadCBEd with *PRNP* Q91X F-sgRNA. AAVs (5.1 kb and 5.0 kb, respectively, including ITRs) encode an EFS promoter, TadCBEd deaminase domain, SauriCas9 or enCjCas9 domain, a UGI domain, and a U6 Pol III cassette expressing either the *PRNP* R37-targeting sgRNA or *PRNP* Q91-targeting sgRNA. **f**, Frequency of the specified stop codon installation in the bulk brain hemisphere of Tg25109 mice, 35 days after treatment with either single-AAV PHP.eB SauriCas9-TadCBEd *PRNP* R37X F-sgRNA (n=6) or single-AAV PHP.eB enCjCas9-TadCBEd *PRNP* Q91X F-sgRNA (n=5). AAVs were retro-orbitally administered to 5–8 weeks-old Tg25109 mice at a total dose of 1.5x10^13^ vg/kg. Dots represent individual biological replicates (n=3 unless noted otherwise) and data are presented as mean values +/- 95% CI.

We also explored the potential of delivering size-minimized CBEs with a single AAV vector^34^ to obviate the need for the manufacture and co-transduction of multiple vectors (Extended Data Fig. 3a). Despite extensive engineering efforts (Methods), such as sgRNA scaffold optimization (Extended Data Fig. 3b,c) and minimization of CBE proteins and AAV *cis* elements (Extended Data Fig. 3d), the two most promising strategies that we tested in vivo—SauriCas9-TadCBEd with *PRNP* R37X-installing F-sgRNA and enCjCas9-TadCBEd with *PRNP* Q91X-installing F-sgRNA (Extended Data Fig. 3e)—did not yield efficient editing in the mouse brain (Extended Data Fig. 3f).

**Extended Data Fig. 4:**
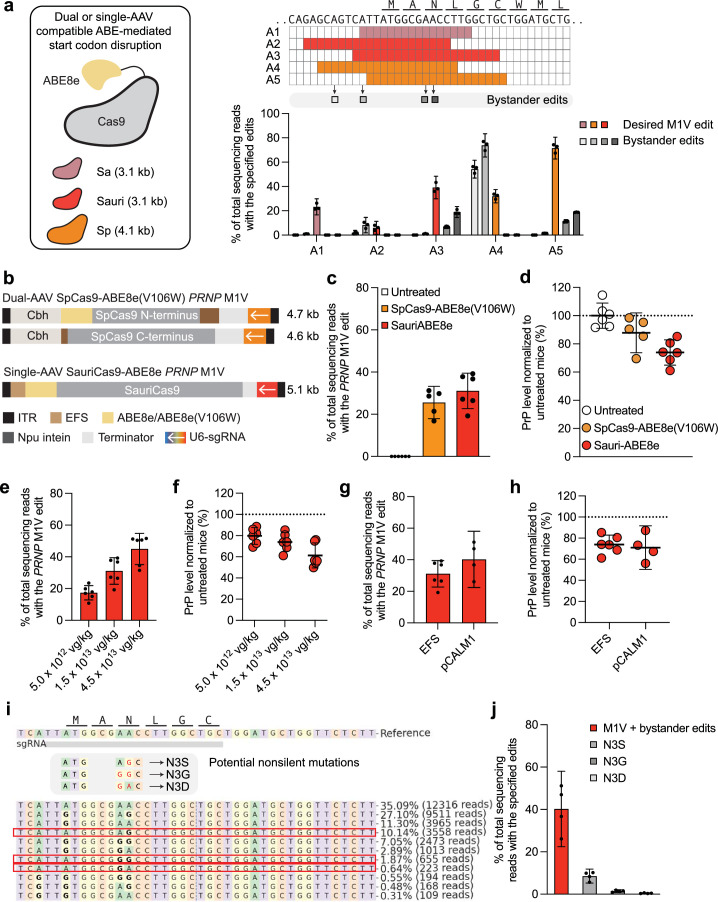
Development of ABE-mediated start codon disruption strategy for PrP reduction. **a**, Schematic of ABE-mediated start codon disruption strategy highlighting sgRNA spacer sequences and potential bystander sites. Frequency of M1V and bystander edits in HEK293T cells transfected with ABEs and corresponding sgRNAs. **b**, Schematics of AAV constructs for dual-AAV PHP.eB SpCas9-ABE8e(V106W) with A5 *PRNP* M1V F+E-sgRNA and single-AAV PHP.eB SauriCas9-ABE8e with A3 *PRNP* M1V F-sgRNA. **c**, Frequency of the desired M1V edit, and **d**, PrP protein level in the bulk brain hemisphere of Tg25109 mice untreated (n=6), or treated with dual-AAV PHP.eB SpCas9-ABE8e(V106W) A5 *PRNP* M1V F+E-sgRNA (n=5) or single-AAV PHP.eB SauriCas9-ABE8e A3 *PRNP* M1V F-sgRNA (n=6). **e** and **f**, Dose-dependent effects of single-AAV PHPe.B SauriCas9-ABE8e (5.0x10^12^ vg/kg, 1.5x10^13^ vg/kg, and 4.5x10^13^ vg/kg, n=6 per dose) on M1V editing frequency (e) and PrP levels (f) in the bulk brain hemisphere of treated mice. Data for the ‘1.5x10^13^ vg/kg’ condition correspond to the ‘SauriCas9-ABE8e’ condition in Extended Data Fig. 4d and e. **g**, Frequency of the desired M1V edit, and **h**, PrP protein level in the bulk brain hemisphere of Tg25109 mice treated with single-AAV PHP.eB SauriCas9-ABE8e with A3 M1V F-sgRNA with either EFS promoter (n=6) or pCALM1 promoter (n=4) driving the expression of the SauriCas9-ABE8e. Data for the ‘EFS’ condition correspond to the ‘SauriCas9-ABE8e’ condition in Extended Data Fig. 4d and e. **i**, Representative allele frequency table showing editing outcome after treatment with single-AAV PHP.eB SauriCas9-ABE8e with A3 *PRNP* M1V F-sgRNA. Incidences where bystander edits occur without on-target editing are highlighted in red box. **j**, Frequency of bystander editing that leads to N3S, N3D, and N3G mutation in genomic DNA harvested from brain hemisphere of Tg25109 mice treated with single-AAV PHP.eB SauriCas9-ABE8e with A3 *PRNP* M1V F-sgRNA, harvested 35 days post-treatment (n=4). Dots represent individual biological replicates (n=3 unless noted otherwise) and data are presented as mean values +/- 95% CI.

We also explored the possibility of using adenine base editor (ABE), which can mediate the conversion of A•T to G•C^12^, to achieve gene silencing by mutating an ATG start codon to GTG or ACG. Among five ABE strategies tested, we advanced two strategies that yielded the highest start codon disruption efficiencies in the cell culture for test in vivo: single-AAV compatible^34^ SauriCas9-ABE8e with A3 *PRNP* M1V F-sgRNA and dual-AAV compatible SpCas9-ABE8e(V106W) with A5 *PRNP* M1V F+E-sgRNA (Extended Data Fig. 4a,b). With a total dose of 1.5 × 10^13^ vg kg^−1^, these ABE strategies yielded 25% and 31% average editing with 12% and 26% PrP reduction in the mouse brain 35 d post treatment (Extended Data Fig. 4c,d), respectively. While increased viral dose (Extended Data Fig. 4e,f) and optimization of the promoter driving the expression of the base editor (Extended Data Fig. 4g,h) improved potency of single-AAV SauriCas9-ABE8e, the consequence of the resulting nonsilent bystander mutations requires further investigation (Extended Data Fig. 4i,j).

Given the combination of efficient on-target editing, potent PrP reduction and absence of nonsilent bystander mutations, we advanced dual-AAV PHP.eB TadCBEd with the *PRNP* R37X F+E-sgRNA for further study.

### Analysis of off-target editing in human cells

To assess Cas-dependent DNA off-target editing in the human genome from the *PRNP* R37X installation strategy, we applied the circularization for in vitro reporting of cleavage effect by sequencing (CIRCLE-seq)^35^ method to genomic DNA from HEK239T cells, nominating 299 human sites as candidate off-target sites associated with the SpCas9 DNA-targeting domain and the *PRNP* R37X sgRNA (Supplementary Table 4). No nominated candidate sites were associated with tumor suppressor genes derived from IntOGen^36,37^.

**Fig. 4:**
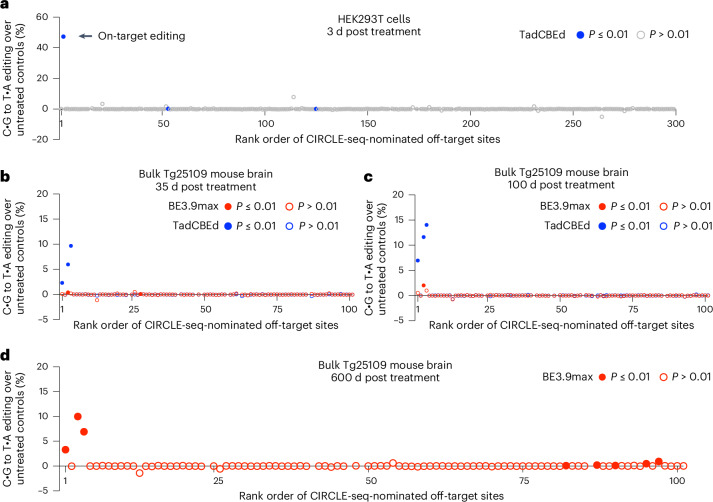
Off-target analysis of R37X base editing strategy in human and mouse genome. **a**, The percentage of C•G-to-T•A substitution in BE-AAV-treated samples above background (untreated samples) at 299 CIRCLE-seq nominated off-target sites in the human genome (GRCh37). Genomic DNA was extracted from HEK293T cells untreated (*n* = 3) or after 3 d following transfection of plasmids encoding TadCBEd and the *PRNP* R37X sgRNA (*n* = 3). Each dot represents mean of three biological replicates. **b**,**c**, The percentage of C•G-to-T•A substitution in BE-AAV-treated samples above background (untreated samples) at the top 100 CIRCLE-seq-nominated off-target sites in the mouse genome (GRCm38). Genomic DNA was extracted from the bulk brain hemisphere of Tg25109 mice untreated (*n* = 6), or 35 d (**b**) and 100 d (**c**) after treatment with dual-AAV PHP.eB BE3.9max with *PRNP* R37X sgRNA (*n* = 6), or dual-AAV PHP.eB TadCBEd with *PRNP* R37X sgRNA (*n* = 6) at a total dose of 1.5 × 10^13^ vg kg^−1^. Each dot represents mean of six biological replicates. **d**, The percentage of C•G-to-T•A substitution in BE-AAV-treated samples above background at the top 100 CIRCLE-seq-nominated off-target sites in the mouse genome (GRCm38). Genomic DNA was extracted from the bulk brain hemispheres of Tg25109 mice untreated (*n* = 5), or 600 d after treatment with dual-AAV PHP.eB BE3.9max with *PRNP* R37X sgRNA (*n* = 5) at a total dose of 1 × 10^14^ vg kg^−1^. Each dot represents mean of five biological replicates. In all panels, significance was calculated by one-tailed Student’s *t*-test. Off-target editing with *P* > 0.01 compared with untreated control is labeled with hollow circles, and those with *P* ≤ 0.01 are labeled with solid circles. Plots showing individual data points and error bars are provided in Supplementary Figs. 1–4.

Next, we measured editing at the CIRCLE-seq-nominated sites in cultured human cells by transfecting HEK293T cells with plasmids encoding TadCBEd and *PRNP* R37X sgRNA. We confirmed potent on-target editing in the treated cells averaging 48% 3 d after transfection (Fig. 4a). We then measured the frequency of off-target editing by analyzing the frequencies of C•G-to-T•A substitutions in each CIRCLE-seq-nominated site. We observed off-target editing significantly higher than background levels of the untreated groups (*P* ≤ 0.01) at two sites (hOT-53 and hOT-125). Off-target editing at the hOT-53 site, located in exon 19 of *CNTNAP1*, was observed at a frequency of 0.29%, leading to an R1058H missense mutation. While some missense mutations in the *CNTNAP1* gene are associated with hypomyelinating neuropathy^38^, the R1058H mutation has not been observed in patients and is not annotated as a pathogenic variant in the ClinVar database^39^. Furthermore, humans with heterozygous R1058H *CNTNAP1* exist in the global population at a frequency of 1/20,000 while it is further enriched in the South Asian population to 1/10,000 in the gnomAD database^40,41^. Therefore, we do not expect low levels of off-target editing at hOT-53 to imply clinical consequence, although additional studies to confidently assess the consequence of this mutation are needed. Off-target editing was observed at a frequency of 0.10% above background at the hOT-125 site, located in an intergenic region of chromosome 2, with no known or anticipated clinical consequence.

### Analysis of off-target editing in mouse tissues

An understanding of how prolonged expression of base editing components from AAV affects the magnitude of off-target edits will help inform the safety of base editing treatments. To assess the off-target editing in a delivery context that recapitulates expression levels in the CNS during therapeutic use, we sought to measure the magnitude of surrogate off-target edits in mouse brain samples treated with base editors over various treatment durations. Using genomic DNA extracted from liver tissue of Tg25109 mice as input DNA, CIRCLE-seq nominated 197 candidate off-target sites in the mouse genome (Supplementary Table 5).

We sequenced the top 100 CIRCLE-seq-nominated off-target sites in the genomic DNA extracted from mouse brains harvested 35, 100 or 600 d following treatment with AAVs encoding CBEs and *PRNP* R37X sgRNA. Specifically, 35-d cohorts received either BE3.9max or TadCBEd treatment at 1.5 × 10^13^ vg kg^−1^ dose, 100-d cohorts received either BE3.9max or TadCBEd treatment at 1.5 × 10^13^ vg kg^−1^ dose and 600-d cohorts received BE3.9max treatment at 1.0 × 10^14^ vg kg^−1^ dose.

In the 35-d cohort, we observed off-target editing significantly higher than background levels of the untreated groups (*P* ≤ 0.01) at two sites (mOT-3 and mOT-28) in BE3.9max-treated samples and at three sites (mOT-1, mOT-3 and mOT-4) in TadCBEd-treated samples (Fig. 4b). In the 100-d cohort, we observed significant off-target editing at only one site (mOT-3) in BE3.9max-treated samples and at three sites (mOT-1, mOT-3 and mOT-4) in TadCBEd-treated samples (Fig. 4c). The increase in off-target editing observed with TadCBEd compared with BE3.9max at both timepoints (14-fold and 5.7-fold higher at mOT-3 site, on average, at 35 d and 100 d, respectively (Fig. 4b,c)) is consistent with the higher activity of TadCBEd and its more efficient on-target editing compared with BE3.9max (4.1-fold and 2.1-fold higher on-target editing at 35 and 100 d, respectively) (Fig. 3f).

In the 600-d cohort treated with BE3.9max at a higher dose of 1.0 × 10^14^ vg kg^−1^, we observed significant off-target editing at eight sites (mOT-1, mOT-3, mOT-4, mOT-82, mOT-87, mOT-90, mOT-95, mOT-97), with elevated levels of off-target editing frequencies at mOT-1, mOT-3 and mOT-4 compared with the 35-d and 100-d cohorts (Fig. 4d). This result is consistent with the higher dose of AAV administered and the longer anticipated duration of the editor expression.

### Enhancing tissue specificity with AAV vector engineering

Given that off-target editing may accumulate over time in tissues where base editor expression persists, editor expression in nontarget tissues may increase the risk of off-target editing without offering any commensurate benefit to patients^42,43^. Prion toxicity is cell autonomous and affects only neurons^44^; astrocytes also replicate prions but do not suffer toxicity, while other cell types appear not to causally contribute to prion disease^45^. To minimize off-target editing risk in tissues and cell types not relevant to prion disease, we sought to enhance the neuronal specificity of base editor expression.

**Fig. 5:**
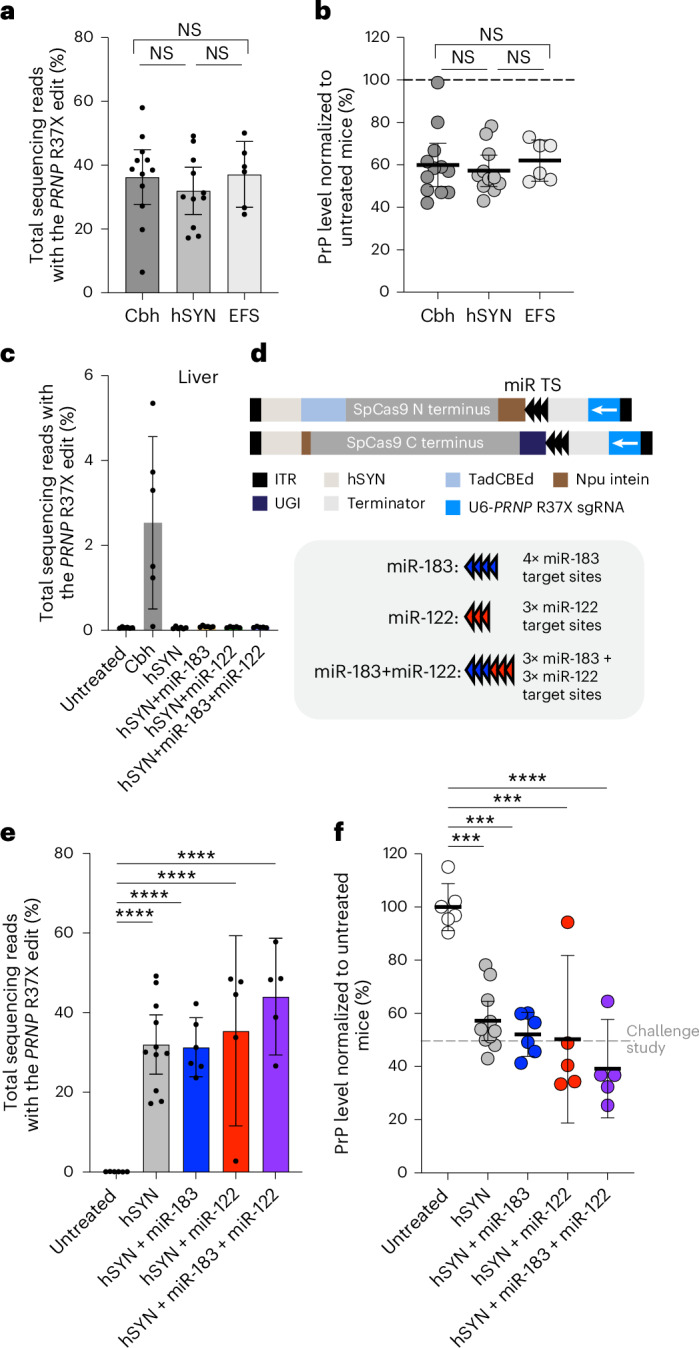
Engineering tissue-specific expression of dual-AAV TadCBEd. **a**,**b**, Frequency of the desired R37X edit (**a**), and PrP protein level in the bulk brain hemisphere (**b**) of Tg25109 mice treated with dual-AAV PHP.eB TadCBEd *PRNP* R37X F+E-sgRNA, with Cbh (*n* = 12), hSYN (*n* = 11) or EFS (*n* = 6) promoter driving the expression of the base editor. Data for the ‘Cbh’ condition correspond to the ‘TadCBEd+*PRNP* R37X F+E-sgRNA’ condition in Fig. 3d,e, and are replotted for comparison. **c**, Frequency of the desired R37X edit in the liver of mice untreated (*n* = 6) or treated with dual-AAV TadCBEd encoding Cbh promoter (Cbh; *n* = 6), hSYN promoter (hSYN; *n* = 6), hSYN promoter plus miR-183 target sites (hSYN+miR-183; *n* = 6), hSYN promoter plus miR-122 target sites (hSYN+miR-122; *n* = 5) or hSYN promoter plus miR-183 and miR-122 target sites (hSYN+miR-183+miR-122; *n* = 5). **d**, Schematic of dual-AAV PHP.eB TadCBEd *PRNP* R37X F+E-sgRNA with hSYN promoter driving the expression of the base editor and miR target site (TS) incorporation. ‘miR-183’ contains 4 copies of miR-183 target sites; ‘miR-122’ contains 3 copies of miR-122 target sites; ‘miR-183+miR-122’ contains 3 copies each of miR-183 and miR-122 target sites. **e**,**f**, Frequency of the desired R37X edit (**e**) (*P* < 0.0001 for all groups versus untreated), and PrP protein level (**f**) in the bulk brain hemisphere of Tg25109 mice harvested 35 d after treatment with dual-AAV TadCBEd, with or without the specified miR target site incorporation (untreated versus hSYN, *P* = 0.0003; untreated versus hSYN+miR-183, *P* = 0.0001; hSYN+miR-122, *P* = 0.0002; untreated versus hSYN+miR-183+miR-122, *P* < 0.0001) at a total dose of 1.5 × 10^13^ vg kg^−1^ (untreated, *n* = 6; hSYN, *n* = 11; hSYN+miR-183, *n* = 6; hSYN+miR-122, *n* = 5; hSYN+miR-183+miR-122, *n* = 5). Data for the ‘hSYN’ condition correspond to the ‘hSYN’ condition in **a** and **b**, and are replotted for comparison. Dots represent individual biological replicates and data are presented as mean ± 95% CI. Significance in **a** and **b** was calculated by one-way ANOVA test with Bonferroni correction; NS, *P* > 0.12. Significance in **e** and **f** was calculated by two-way ANOVA test with Dunnett’s correction; ***P < 0.0002; ****P < 0.0001.

**Extended Data Fig. 5:**
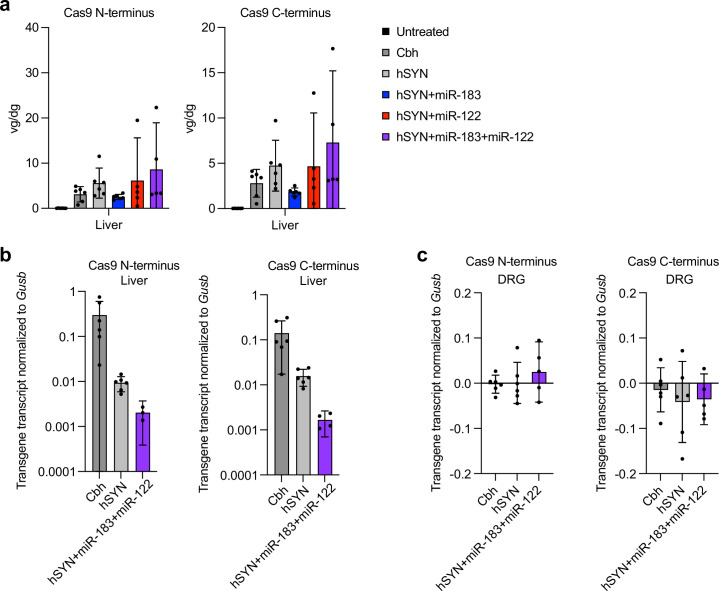
Characterization of tissue-specific expression of dual-AAV PHP.eB TadCBEd with *PRNP* R37X F+E-sgRNA strategy. **a**, Viral genome concentration normalized to the diploid genome (vg/dg) in the liver of Tg25109 mice untreated (n=6), or treated with dual-AAV PHP.eB TadCBEd *PRNP* R37X F+E-sgRNA with the Cbh promoter (Cbh; n=6), hSYN promoter (hSYN; n=6), hSYN promoter with miR-183 target sites incorporation (hSYN+miR-183; n=6), hSYN promoter with miR-122 target sites incorporation (hSYN+miR-122; n=5), or hSYN promoter with miR-183 and miR-122 target sites incorporation (hSYN+miR-183+miR-122; n=5) at a total dose of 1.5x10^13^ vg/kg. ddPCR was performed with probes specific for Cas9 N- and C-terminus, and *Actb* housekeeping gene. **b** and **c**, Cargo transgene transcript normalized to *Gusb* transcript in the liver (b) and DRG (c) of mice treated with dual-AAV PHP.eB TadCBEd *PRNP* R37X F+E-sgRNA, with Cbh promoter (Cbh; n=6), hSYN promoter (hSYN; n=6), or hSYN promoter with miR-183 and miR-122 target site incorporation (hSYN+miR183+miR122; n=4). ddPCR was performed with probes specific for Cas9 N- and C-terminus, and *Gusb* transcript. Dots represent individual biological replicates and data are presented as mean values +/− 95% CI.

We first tested different promoters to drive the expression of TadCBEd in the AAV transgene expression cassette. With the Cbh promoter in the canonical v5 BE-AAV architecture that induces robust and ubiquitous expression serving as the benchmark, we tested the smaller ubiquitous EFS promoter and the neuron-specific hSYN promoter^46,47^. At 5 weeks after injection, we observed comparable editing efficiencies among three groups (Fig. 5a). Importantly, potent PrP reduction was observed using the neuron-specific hSYN promoter (43% average reduction), similar to that of the Cbh and EFS promoters (40% and 38% average reduction, respectively) (Fig. 5b), consistent with the mainly neuronal expression of PrP. In the liver, a tissue that is transduced by CNS-tropic AAV capsids^19,48^, we observed 2.5% average editing in the Cbh promoter cohort, while editing levels were below the limit of detection (<0.1%) in the hSYN promoter cohort (Fig. 5c). Assessment of viral genome levels revealed comparable transduction in the two treatment groups in the liver (Extended Data Fig. 5a), confirming that restricted expression from the hSYN promoter, rather than reduced viral transduction, accounts for the reduction in liver editing (Extended Data Fig. 5b). These findings suggest that use of the hSYN promoter can increase the specificity of base editing for neuronal over non-neuronal cell types.

Transgene overexpression in dorsal root ganglion (DRG) and liver has been linked to toxicity with high-dose AAV treatment^49,50^. Previous studies demonstrated that by incorporating target sites for microRNAs (miRs) abundantly expressed within nontarget cell types but not in target cell types, transgene expression in nontarget cell types can be selectively repressed by miR-mediated downregulation of transgene expression^49,50^. Therefore, we assessed how miR-183 (abundantly expressed in the DRG) and miR-122 (abundantly expressed in the liver) target site incorporation in the 3′ untranslated region of base editor transcripts in the AAV expression cassette, both separately and in combination, along with how the hSYN promoter affects *PRNP* base editing efficiency in target and nontarget tissues (Fig. 5d).

Analysis of editing efficiencies in the bulk brain hemisphere 5 weeks after injection of dual-AAV containing miR target sites in the hSYN promoter driving base editor expression demonstrated that miR target site incorporation unexpectedly increased editing efficiencies compared with use of the hSYN promoter without miR target sites (hSYN). The hSYN+miR-183+miR-122 cohort demonstrated the most efficient editing (44%), compared with 32% average editing for the hSYN group (Fig. 5e). Correspondingly, we observed potent reduction of PrP in the bulk brain hemisphere with hSYN+miR-183+miR-122 (63%, compared with 43% average reduction for hSYN) (Fig. 5f).

Transgene expression in DRG was not detected (Extended Data Fig. 5c), and therefore the extent to which miR-183 target site incorporation reduces transgene expression in the DRG cannot be accurately assessed in the context of our experiments and warrants future investigation in a model in which DRG toxicity is more pronounced, such as in nonhuman primates. Editing in the liver was absent in all conditions with hSYN promoter, while transgene expression in the liver was further reduced in the hSYN+miR-183+miR-122 cohort compared with the hSYN group (Extended Data Fig. 5b), substantiating the value of multiplexing a cell-type-specific promoter with miR target site incorporation to further reduce transgene expression in nontarget cell types. Given that miR-183 is conserved in mice, monkeys and humans^49^, and miR-122 is conserved in virtually all mammals^51^, the incorporation of miR-183 and miR-122 target sites should serve as a strategy with cross-species relevance.

Overall, our data suggest that a base editing strategy of dual-AAV PHP.eB TadCBEd *PRNP* R37X with the hSYN promoter and miR-183 and miR-122 target sites offers the most promising combination of on-target editing efficiency and reduced risk of undesired editing among the strategies tested.

## Discussion

Prion disease currently has no approved therapy. While several small-molecule drugs have shown promise in treating prion infections in wild-type mice, they have failed to demonstrate efficacy in humanized mouse models infected with human prion isolates^23–26^, highlighting the need for a therapeutic strategy that can address human prion isolates across diverse prion etiologies. ASO-mediated PrP knockdown is showing promise in Phase I of clinical trial, but the transient nature of ASOs requires repeated dosing. In this study, we demonstrated that treatment of humanized *PRNP* mice with PrP-reducing BE-AAVs substantially extends lifespan after challenge with two distinct human pathogenic prion isolates.

Our human pathogenic prion challenge study was performed at a 1 × 10^14^ vg kg^−1^ total dose level, which, though clinically precedented^52^, is associated with substantial safety risks^30,31^. To reduce the therapeutic dose and improve therapeutic potential, we further optimized PrP-reducing BE-AAVs to be more potent, and engineered the AAV genome expression cassette to limit expression in key nontarget tissues. We were not able to assess the extent of lifespan extension with the optimized construct as our institution revised its biosafety policy to eliminate future challenge studies using human prions following the report of a second occupationally acquired case of prion disease and a moratorium on prion research in France^53^. However, previous studies examining ASO-mediated PrP-lowering strategy demonstrated a correlation between PrP reduction and lifespan extension^2^, suggesting that the optimized BE-AAV construct is expected to further improve lifespan of prion-infected mice beyond the 52% lifespan extension achieved with the original BE3.9max *PRNP* R37X strategy.

This study has several limitations that warrant future investigation. First, off-target editing for in vivo genome editing is challenging to predict in a preclinical setting, as clinically relevant exposure of human cells and tissues to the editors cannot be modeled. While we did not detect any significant guide-dependent off-target editing of anticipated clinical consequence in HEK293T cells after transfection of TadCBEd and *PRNP* R37X sgRNA plasmids, future studies are needed to investigate off-target editing in more clinically relevant contexts. We did not assess RNA off-target editing or guide-independent genomic DNA off-target editing, as both have been previously characterized for the base editors we used^32,54^ and are not dependent on the guide RNA^55^. While the more potent TadCBEd editor used in our optimized dual-AAV BE *PRNP* R37X constructs has been shown to have a lower propensity for guide-independent and RNA off-target editing than previously engineered cytosine deaminase-based editors (such as BE3.9max)^32,54^, further characterization of these off-target editing events in tissues of therapeutic relevance will help inform this potential risk of BE-AAV treatment.

During the course of this study, an elegant targeted epigenetic approach using coupled histone tail for autoinhibition release of methyltransferase (CHARM) to achieve mouse PrP reduction was reported^7^. While CHARM demonstrated potent PrP reduction via methylation of the mouse *Prnp* promoter, the study was performed in wild-type mice using mouse *Prnp* promoter-targeting agents. The development of targeted epigenetic approaches targeting human *PRNP*, as well as validation of the long-term durability of CHARM-mediated epigenetic silencing in vivo, understanding the extent and consequences of off-target methylation and silencing, and characterizing efficacy against prion disease progression, would further advance the therapeutic relevance of epigenetic approaches, which offer strengths that complement those of gene-editing approaches.

Base editing to permanently reduce PrP levels represents a substantial advance in potential strategies to ameliorate prion diseases. Although we used the mouse-specific capsid PHP.eB in this study, the PrP-reducing BE-AAV strategies described here are compatible with human-blood–brain barrier-crossing AAV serotypes such as BI-hTFR1 (ref. ^56^), which may provide broad CNS distribution in humans. Future studies extending the findings of this work, such as a more thorough assessment of biodistribution, characterization of possible immune responses to AAV and the base editor transgene, and the therapeutic benefit of treatments at later timepoints, may eventually provide patients with a one-time treatment option that can ameliorate all types of prion disease.

## Methods

### Ethics statement

Human brain tissue for challenge studies was provided by the National Prion Disease Pathology Surveillance Center (NPDPSC; Cleveland, OH). Use of human tissue was approved under NPDPSC Institutional Review Board (IRB) protocol 01-14-18 and Safar lab IRB protocol 03-14-28. Broad Institute Office of Research Subject Protection (ORSP) determination NHSR-5934 ruled that the use of human brain tissue for challenge studies was not human subject research. Animal experiments were approved by the Broad Institute Institutional Animal Care and Use Committee (D16-00903; 0162-05-16-2 and 0048-04-15-2).

### Molecular cloning

Editor and sgRNA plasmids were cloned using USER assembly with USER enzyme (New England Biolabs, cat. no. M5505L) or Gibson assembly with NEBuilder HiFi DNA Assembly Master Mix (New England Biolabs, cat. no. E2621L). Plasmids encoding recombinant AAV (rAAV) genomes were cloned by restriction digestion of v5 AAV CBE (Addgene, cat. no. 137176) or single-BE-AAV (Addgene, cat. no. 189925) followed by Gibson assembly with eBlock fragments (IDT) or PCR amplicons. DNA was PCR amplified with Phusion U Green Multiplex PCR Master Mix (Thermo Fisher Scientific, cat. no. F564S). Plasmids were transformed into Mach1 (Thermo Fisher Scientific, cat. no. C862003) or NEB Stable (New England Biolabs, cat. no. C3040H) chemically competent *Escherichia coli* and were prepared using Plasmid Plus Midiprep kits (Qiagen, cat. no. 12945).

### Cell culture

HEK293T cells (ATCC, cat. no. CRL-3216) were cultured in DMEM plus GlutaMAX (Thermo Fisher Scientific, cat. no. 10569044) supplemented with 10% (v/v) fetal bovine serum (FBS). HEK293T clone 17 cells (ATCC, cat. no. CRL-11268) were maintained in either DMEM plus GlutaMAX (Thermo Fisher Scientific, cat. no. 10569044) supplemented with 10% (v/v) FBS or in F17 media (Thermo Fisher, cat. no. A1383501). Cells were maintained at 37 °C with 5–8% CO_2_. Cell lines were tested negative for mycoplasma during the course of this study.

### Plasmid transfection of HEK293T cells

Cells were seeded in 96-well plates (Corning, cat. no. 353075) at a density of 15,000–20,000 cells per well. At 16–24 h after seeding, 200 ng of editor plasmids and 40 ng of sgRNA plasmids were diluted in Opti-MEM (Life Technologies, cat. no. 31985070), and were mixed with 0.5 μl of Lipofectamine 2000 (Invitrogen, cat. no. 11668500). Sequences of sgRNAs evaluated in this study are provided in Supplementary Table 6. After incubation at room temperature for 10 min, the transfection mix was added directly to the cells. Unless specified otherwise, genomic DNA was isolated 72 h after transfection, by incubating with 50 μl of lysis buffer per well (10 mM Tris–HCl pH 8.0, 0.05% SDS and 25 μg ml^−1^ proteinase K) at 37 °C for 1 h, followed by 80 °C for 30 min.

### HTS of genomic DNA samples and data analysis

Primer sequences and the corresponding amplicon sequences are listed in Supplementary Table 7. Briefly, 1 μl of cell lysate containing the genomic DNA was used as an input in the first round of PCR (PCR1) for the amplification of the target locus. PCR1 was performed either using Phusion U Green Multiplex PCR Master Mix (Thermo Fisher Scientific, F564S) under the following conditions: 98 °C (3 min); 25 cycles of 98 °C (10 s), 61 °C (20 s) and 72 °C (40 s); and 72 °C (2 min), or by quantitative PCR (qPCR) using SYBR Green fluorescence to monitor the PCR1 reaction and stop at the exponential phase to avoid over-amplification of the target locus. Then, 1 μl of PCR1 product was subsequently used as an input for the second round of PCR (PCR2) to append unique Illumina barcodes. PCR2 was performed using Phusion U Green Multiplex PCR Master Mix (Thermo Fisher Scientific, cat. no. F564S) under the following conditions: 98 °C (3 min); 10 cycles of 98 °C (10 s), 60 °C (20 s) and 72 °C (30 s); and 72 °C (2 min). PCR2 products were pooled and gel purified using Qiaquick Gel Extraction Kit (Qiagen, cat. no. 28704). The pooled library was quantified by Qubit dsDNA HS Assay kit (Thermo Fisher Scientific, cat. no. Q32852) and was sequenced using Illumina MiSeq 300 v2 Kit (Illumina) on an Illumina MiSeq instrument.

HTS reads were demultiplexed by MiSeq Reporter software v.2.6 (Illumina). Data analysis was performed using CRISPResso2 (v.2.2.12), with minimum average quality score (‘-q’) set to 30, ‘discard_indel_reads’ set to TRUE and the quantification window (‘-w’) set to 10. Base editing efficiency was calculated as the percentage of reads containing the specified edit at a given position without indels divided by the number of total aligned reads. Indels were calculated as the percentage of reads for discarded reads divided by the number of total aligned reads. The lower limit of detection defined by the error rate of the HTS method is assumed to be 0.1%.

### Antibody staining and flow cytometry

At 6 d after plasmid transfection, HEK293T cells were washed with flow cytometry buffer (1 × PBS supplemented with 5% FBS) and were incubated with Alexa647-conjugated anti-CD230 6D11 antibody (BioLegend, cat. no. 808008) diluted 1:100 with flow cytometry buffer. Cells were incubated with the antibody for 30 min on ice in the dark. Subsequently, cells were washed two times with 100 μl of flow cytometry buffer and analyzed with flow cytometry using the CytoFLEX LX Flow Cytometer (Beckman Coulter, cat. no. C06779) at the Broad Institute Flow Cytometry Core, with CytExpert Acquisition and Analysis Software (v.2.4).

### AAV production

AAVs were produced with either adherent cell culture or suspension cell culture. Adherent cell AAV production was performed as previously described^18,57^. Briefly, HEK293T clone 17 cells were plated in 150-mm^2^ dishes at a density of 1 × 10^7^ cells per dish. At 18–22 h after seeding, 5.7 μg of AAV vector plasmid, 11.4 μg of pHelper plasmid and 22.8 μg of rep-cap plasmid were mixed with PEI MAX polyethyleneimine transfection reagent (Polysciences, cat. no. 24765) and were added to each 150-mm^2^ plate. Media change was performed with 5% (v/v) FBS supplemented DMEM plus GlutaMAX 24 h after transfection. Suspension cell AAV production was performed by seeding 1 × 10^6^ cells per ml in 200–1000 ml of F17 expression media (Thermo Fisher, cat. no. A1383501) incubated at 37 °C and 8% CO_2_, and transfecting cells 24 h after seeding with 2 μg of total DNA per million cells (AAV vector plasmid, pHelper plasmid and rep-cap plasmid in 1:2:1 ratio) with Transport 5 PEI Max transfection reagent at 2:1 ratio to the total DNA (Polysciences, cat. no. 26008).

At 72 or 96 h after transfection, media and cells were collected and centrifuged at 2,000*g* for 10 min. Cell pellets were resuspended in hypertonic lysis buffer (40 mM Tris base, 500 mM NaCl, 2 mM MgCl_2_) supplemented with 100 U ml^−1^ salt active nuclease (ArcticZymes, cat. no. 70920) and incubated at 37 °C for 1 h. Then, 5 × PEG solution (40% PEG 8000 (Sigma Aldrich, cat. no. 89510) and 2.5 M NaCl in water) was added to the media and was incubated at 4 °C overnight or for at least 2 h. After PEG precipitation, the media was centrifuged at 3,000*g* for 30 min. The pellets were resuspended in the hypertonic lysis buffer and combined with the resuspended cell pellets. The combined lysates were centrifuged at 3,000*g* for 10 min. The supernatant was transferred into ultracentrifuge tubes (Beckman Coulter, cat. no. 342414), followed by sequential addition of the 15%, 25%, 40% and 60% iodixanol gradients. Ultracentrifugation was performed with Ti 70 rotor in an Optima XPN-100 Ultracentrifuge (Beckman Coulter) at 68,000 rpm (340,000*g*) for 1 h, or a Sorvall WX+ Ultracentrifuge (Thermo Scientific, cat. no. 75000090) at 67,000 rpm (330,000*g*) for 1 h and 15 min. Subsequently, the solution containing AAVs at the interface of 40–60% iodixanol gradient was withdrawn with a syringe and needle. The solution was dialyzed through PES 100-kD MWCO columns (Thermo Fisher Scientific, cat. no. 88532) to perform buffer exchange to PBS supplemented with 0.001% Pluronic F-68 (MP Biomedicals, cat. no. 2750049). The concentrated AAV was filtered through a sterile 0.22-μm column (Millipore, cat. no. UFC30GC0S) and was stored at 4 °C. Titer was quantified using AAVpro Titration Kit version 2 (Takara Bio, cat. no. 6233) using qPCR according to the manufacturer’s protocol.

### Animals

Initial in vivo experiments for injection route optimization (Extended Data Fig. 2) used the Tg66 humanized mouse line^21^, a generous gift from the Research Foundation for Mental Hygiene facilitated by National Institutes of Health (NIH) Rocky Mountain Laboratories. All subsequent experiments involving human gene-targeting reagents utilized Tg25109 mice provided by Prion Alliance. These animals were heterozygous for the Tg25109 transgene array, which contains three copies of a human 129M PRNP bacterial artificial chromosome, on a background of endogenous *Prnp* knockout (ZH3/ZH3)^58^ on a mixed C57BL6N/J background. Animal experiments were approved by the Broad Institute Institutional Animal Care and Use Committee (D16-00903; 0162-05-16-2 and 0048-04-15-2). Mouse housing facilities were maintained at 20–22 °C with 30–50% humidity. Mice were kept on a 12-h light/dark cycle with ad libitum access to standard rodent diet and water. Both sexes were included for each experimental condition in in vivo experiments involving mouse models. Both sexes were assigned to each experimental group as evenly as possible, availability of mice permitting. Following AAV treatment, animals were monitored by body score after the treatment, and monitored for signs of pain or distress (including lethargy, notable hair loss, loss of body weight of 20% or more from pre-injection baseline), respiratory distress, neurological deficits, dehydration or inability to access food or water. Animals that met these predefined humane endpoint criteria were euthanized and excluded from the studies.

### Intracerebroventricular injections

Intracerebroventricular injections were performed on mice at 4 weeks of age. Anesthesia was induced with 4% isoflurane and maintained with 1.5–2.5% isoflurane. A subcutaneous injection of meloxicam at 0.6–2 mg kg^−1^ was given as a prophylactic analgesic. Animals were immobilized on a heating pad using a Kopf stereotaxic apparatus (David Kopf Scientific Instruments) using ear bars and tooth bar to immobilize the skull. AAV solution was injected bilaterally at 500 nl min^−1^ via Hamilton syringe (Hamilton Company, cat. no. 88011) driven by a Micro4 microsyringe pump (WPI) at coordinates (+0.3 mm, ±1 mm, −3 mm).

### Retro-orbital injections

Before injection, AAVs were diluted with sterile 0.9% Sodium Chloride injection solution (Covetrus, cat. no. 061758) to formulate doses indicated, with an approximate injection volume of 100 μl per mouse. Anesthesia was induced and maintained using inhaled isoflurane at 2–3%. Mice were weighed before the injection, and the injection volume of the AAV was calculated based on the weight of the mice. AAVs were administered intravenously into the right retro-orbital sinus of the animal using a 300-μl insulin syringe with a 31 G needle (Becton Dickinson, cat. no. 328438). Immediately following injection, one drop of 0.5% proparacaine hydrochloride ophthalmic solution (Patterson Veterinary, cat. no. 07-885-9765) was applied topically to the eye.

### Nuclear isolation and sorting

Nuclei isolation was performed as previously described^18,57^. Briefly, the frozen brain tissue parts were transferred to the Dounce homogenizer (Sigma Aldrich, cat. no. D8938) and were homogenized in 2 ml of EZ-PREP buffer (Sigma Aldrich, cat. no. NUC-101) for 20 strokes with pestle A and pestle B. The homogenates were transferred to a new tube containing 2 ml of additional EZ-PREP buffer. The homogenates were centrifuged at 500*g* for 5 min. Supernatant was decanted and the nuclei pellets were resuspended in 4 ml of Nuclei Suspension Buffer (ice-cold PBS supplemented with 100 μg ml^−1^ BSA and 33 μM Vybrant DyeClycle Ruby (Thermo Fisher, cat. no. V10309)) and centrifuged at 500*g* for 5 min. After an additional centrifugation and resuspension step, the pellet was resuspended in Nuclei Suspension Buffer (1 ml for cortex, 1.5 ml for midbrain and 0.5 ml for cerebellum). The solution was filtered through a 35-μm cell strainer. FACS was performed on the SONY MA900 Cell Sorter (Sony Biotechnology). The nuclei were sorted directed into the lysis buffer (DNAdvance Lysis Buffer (Beckman Coulter, cat. no. A48705) supplemented with dithiothreitol and proteinase K).

### Prion challenge studies

At 1 week after AAV treatment, animals in the human prion isolate inoculation cohort were infected by intracerebral prion inoculation with 30 μl of a 1% brain homogenate as previously described^2,59^. Sample RES-03 was frontal cortex from autopsy-confirmed sporadic CJD type MM1 and sample RES-07 was frontal cortex from autopsy-confirmed E200K with codon 129 genotype MM. Brain tissue was prepared as reported previously^2^. Briefly, brains were homogenized at 10% (w/v) in PBS (Gibco, cat. no. 14190) using 3 × 40-s high pulses in 7-ml tubes with zirconium oxide beads (Precellys, cat. no. KT039611307.7) in a Minilys tissue homogenizer (Bertin Technologies, cat. no. EQ06404-200-RD000.0). Homogenates were diluted to 1% (w/v) in more PBS (Gibco, cat. no. 14190), irradiated on dry ice with 7.0 kGy of X-rays and passed through progressively finer blunt needles (Sai Infusion, B18, B21, B24, B27, B30). Homogenates were pipetted into glass vials with removable caps and then injected through 31 G disposable syringes (Becton Dickinson, cat. no. 328449) into sealed amber glass vials. The homogenates were freehand inoculated to isoflurane-anesthetized animals between the right ear and midline. Baseline weights were taken at 16 weeks of age (which corresponded to 43–86 dpi depending upon the mouse’s age at inoculation). Inoculated mice were then monitored for general health, nest-building behavior and body weights weekly, beginning at 112 dpi. Nest-building behavior was rated on a scale of 0 to 2 by examining cotton square nestlets (Ancare) and Enviro-dri packed paper (Shepherd): 0 = unused; 1 = used but flat; 2 = pulled into three-dimensional nest structure, with values of 0.5 and 1.5 permitted. The pre-specified endpoint was 20% weight loss relative to the baseline or moribund meaning unable to reach food or water. Animals that met the predefined endpoint criteria were euthanized by CO_2_ inhalation followed by cervical dislocation. All remaining animals were euthanized at 600 dpi.

### Mouse tissue collection, homogenization and genomic DNA extraction

At collection, mice were euthanized by CO_2_ asphyxiation followed by cervical dislocation. Bulk tissue was harvested only for non-prion-infected mice. For harvest of brain tissue, after retrieval of the brain from the skull, the brain stem was removed to maintain consistency across samples, then hemispheres were split sagittally. All tissues were flash-frozen on dry ice and stored at −80 °C until further processing. For homogenization of the brain tissues, cold lysis buffer (0.2% w/v CHAPS, 1 × PBS and 1 tablet of protease inhibitor (Sigma Aldrich, cat. no. 04693159001)) was added to the brain tissue samples at 10% w/v, followed by homogenization on a Bertin MiniLys homogenizer in 7-ml tubes pre-loaded with zirconium oxide beads (Precellys, cat. no. KT039611307.7) using 3 × 40-s pulses. Brain homogenates were aliquoted into 40-μl aliquots for PrP quantification and 300-μl aliquots for genomic DNA extraction and stored at −80 °C until further analysis. The brain homogenates for genomic DNA extraction were incubated with proteinase K (Thermo Fisher, cat. no. EO0491) overnight at 55 °C. Liver tissue samples were incubated in DNAdvance lysis buffer (Beckman Coulter, cat. no. A48705) supplemented with 25 mM dithiothreitol and proteinase K (Thermo Fisher, cat. no. EO0491) overnight at 55 °C with shaking at 800 rpm. The genomic DNA was subsequently purified using DNAdvance kit (Beckman Coulter, cat. no. A48705) following the manufacturer’s protocol, and was used as an input for PCR1 as described above for HTS sample preparation.

### PrP quantification

Quantification of PrP in brain tissue utilized frozen whole hemispheres and a previously described in-house ELISA assay^60^. Briefly, the ELISA assay uses EP1802Y antibody (Abcam, cat. no. ab52604) for capture and biotinylated 8H4 antibody (Abcam, cat. no. ab61409) for detection, diluted to 2.0 μg ml^−1^, followed by streptavidin-HRP (Thermo Fisher Scientific, cat. no. 21130) and TMB (Cell Signaling, cat. no. 7004P4). The standard curve was recombinant mouse PrP (MoPrP23-231) generated in-house^61^. The assay is validated to demonstrate identical reactivity for human and mouse PrP^60^. Results were normalized to the mean of untreated controls and expressed as a percentage residual PrP.

### Assessment of single-AAV compatible CBE strategy

To explore the potential of delivering CBEs in a single AAV vector, five size-minimized CBEs were constructs by fusing TadCBEd and one UGI domain to compact Cas9 domains, including phage-associated continuous evolution (PACE)-evolved *Neisseria meningitidis* 2 Cas9 (eNme2Cas9; 1,080 amino acids)^62^, engineered *Camphylobacter jejuni* Cas9 (enCjCas9; 983 amino acids)^63^, PACE-evolved *C. jejuni* Cas9 (evoCjCas9; 983 amino acids)^64^ and *Staphylococcus auricularis* Cas9 (SauriCas9; 1,060 amino acids)^65^ (Extended Data Fig. 3a). A total of 19 sgRNAs capable of installing premature stop codons at a variety of *PRNP* positions were designed (Trp 7, Trp 31, Arg 37, Gln 41, Gln 52, Gln 59, Gln 67 or Gln 91). Plasmid transfection and HTS analysis were performed as described above to assess editing efficiencies in vitro.

Further sgRNA scaffold optimization was performed for SauriCas9-TadCBEd with *PRNP* R37X sgRNA which showed the highest editing efficiency among all candidates, and enCjCas9-TadCBEd with *PRNP* Q91X sgRNA which uses the smallest base editor tested. The canonical sgRNA scaffolds for SaCas9 (ref. ^66^) (which is commonly used for SauriCas9) and CjCas9 (ref. ^67^) (which is used for enCjCas9) both contain a stretch of four U bases that are susceptible to premature transcription termination with U6 promoter. Therefore, four sgRNA scaffolds were designed by converting one of the four U•A within this stretch to A•U for both enCjCas9 and SauriCas9 sgRNAs (Extended Data Fig. 3b,c). For the enCjCas9-TadCBEd strategy, U_3_F-sgRNA (in which the third U•A is converted to A•U) which showed the highest editing efficiency was chosen to be incorporated in the AAV construct, and it was named as enCjCas9-TadCBEd *PRNP* Q91X F-sgRNA. For the SauriCas9-TadCBEd strategy, U_3_F-sgRNA (in which the third U•A is converted to A•U) which also showed highest editing efficiency was chosen to be incorporated in AAV constructs, and it was named as SauriCas9-TadCBEd *PRNP* Q91X F-sgRNA.

When the previously reported single-AAV ABE architecture^34^ is directly used to package SauriCas9-based CBEs, due to its large Cas9 domain (1,060 amino acids), the size of the transgene is 5.2 kb including inverted terminal repeats (ITRs), exceeding the optimal packaging capacity of AAV (≤4.7 kb including ITRs^68^). Therefore, further SauriCas9-TadCBEd minimization was conducted by testing various smaller nuclear localization signals, the linker between the TadCBEd deaminase and the N terminus of SauriCas9 domain (linker 1), and the linker between the C terminus of SauriCas9 domain and the UGI domain (linker 2) (Extended Data Fig. 3d). SauriCas9-TadCBEd with size-minimized nuclear localization signal and linker 1 sequence that showed similar editing efficiency to the canonical SauriCas9-TadCBEd was incorporated in the single-AAV construct. Furthermore, bovine growth hormone-derived poly(A) (224 base pairs (bp)) was substituted with synthetic poly(A) (49 bp) in the single-AAV construct (Extended Data Fig. 3e).

### CIRCLE-seq off-target nomination

CIRCLE-seq off-target nomination was performed as previously described^35,69^. Briefly, the genomic DNA extracted from HEK293T cells and Tg25109 mouse liver tissues was used as an input to generate DNA fragments with an average length of 300 bp using Covaris S2 instrument. The sheared DNA fragments were subsequently processed to generate circularized DNA using the KAPA HTP Library Preparation Kit (KAPA Biosystems, cat. no. KK8235) as previously described^70^. In vitro cleavage reactions were performed using the circularized DNA, purified Cas9 nuclease protein (New England Biolabs, cat. no. M0386) and synthetic sgRNA with the standard 2′-O-methyl modification at the first three and last three bases, with the following spacer sequence ‘GGCAGCCGAUACCCGGGGCA’, corresponding to the *PRNP* R37X sgRNA. Cleaved products were prepared for HTS as previously described^70^. Libraries were sequenced with 150-bp/150-bp paired-end reads with an Illumina MiSeq instrument. The data analyses were performed using the open-source CIRCLE-seq analysis software.

### rhAmpSeq off-target site amplification and analysis

Ensembl Variant Effect Predictor (VEP)^71^ was used to determine the genomic location of all candidate off-target loci in the human genome nominated by CIRCLE-seq. To perform VEP on human amplicons, hg19 coordinates were lifted over to GRCh38 using UCSC’s liftOver tool. All loci nominated by CIRCLE-seq were checked against a list of tumor suppressors derived from IntOGen^36,37^ and no tumor suppressor genes were found in the list. A pooled sequencing primer was generated for nominated mouse and human off-target sites using the rhAmpSeq design tool (IDT). Genomic DNA was extracted from editor-treated mouse whole brain hemispheres (mouse off-targets) and HEK293T cells (human off-targets). Genomic DNA inputs were amplified with rhAmpSeq pooled sequencing primers according to the manufacturer’s protocol. The amplified libraries were sequenced with 300-bp single-end reads with an Illumina MiSeq or NextSeq instrument. Sequences for rhAmpSeq amplicons were extracted using the R Bioconductor BSGenome package (v.1.4.3) using the GRCm38/mm10 (mouse) and GRCh37/hg19 (human) reference genomes. CRISPResso2 (ref. ^72^) was used to align the rhAmpSeq reads to the amplicon reference sequences and quantify the number of reads with each possible edit. CRISPResso2 output was further processed using a custom Python 3.9.2 script. For each amplicon, the targeting strand was determined by matching the sgRNA spacer sequence or its reverse complement to the reference sequence. Reads with at least one C•G-to-T•A substitution on the edited strand are determined as ‘edited’; all other nucleotide changes were excluded as inconsistent with the base editor’s mechanism. For each amplicon, proportion edited was set to number of edited divided by total reads. Finally, all allele editing results were merged across all samples, grouped by amplicon and treatment, and the mean percentage edited was computed. Because some amplicons exhibited high background ‘editing’ (likely due to sequencing error noise) in the untreated groups, the proportion edited in the untreated group was subtracted from the proportion in the treated group for the purposes of data visualization. Differences between groups were assessed using one-tailed Student’s *t*-test, testing only the hypothesis that the proportion edited is higher in the treated group than the untreated. *P* values less than 0.01 were considered nominally significant. Source code for off-target analyses is available in the study’s online GitHub repository (see below).

### Droplet digital PCR

Genomic DNAs isolated from the mouse tissues as described above were used as an input for droplet digital PCR (ddPCR) for the quantification of the viral genome concentrations. ddPCR Supermix for Probes (No dUTP) (Bio-Rad, cat. no. 1863024) was mixed with the input DNA, the target primers and probe mix (primer and probe sequences listed in Supplementary Table 7), the reference mouse *Actb* CNV primer and probe mix (Bio-Rad, cat. no. dMmuCNS292036842) and 6.25 U of HindIII-HF (New England Biolabs, cat. no. R3104S).

RNA extraction from the liver and DRG of Tg25109 mice was performed using Qiagen AllPrep DNA/RNA Kit (Qiagen, cat. no. 80204) according to the manufacturer’s protocol. Complementary DNA (cDNA) was generated using the SuperScript III First-Strand Synthesis Supermix (Thermo Fisher Scientific, cat. no. 18080400) for input to quantify the transgene expression. dPCR Supermix for Probes (No dUTP) (Bio-Rad, 1863024) was mixed with the input cDNA, the target primers and probe mix (primer and probe sequences listed in Supplementary Table 7), the reference mouse *Gusb* GEX primer and probe mix (Bio-Rad, cat. no. dMmuCPE5096673) and 6.25 U of HindIII-HF (New England Biolabs, cat. no. R3104S). RNA samples were used as an input as negative controls for ddPCR to ensure minimal DNA contamination in the RNA transgene expression analysis.

ddPCR was performed on the QX ONE ddPCR System, with the following PCR program: 95 °C (10 min); 50 cycles of 94 °C (30 s) and 58 °C (1 min); and 98 °C (10 min). The data acquisition and analysis were performed on the QX Manager Software, Standard Edition v.1.4.0.

### Statistics and reproducibility

Data are presented as mean ± 95% confidence interval (95% CI). The sample size and the statistical tests used for each figure are described in the figure legends. Statistical tests were conducted using GraphPad Prism software. Sex disaggregated numbers for individual experiments are provided in Supplementary Table 8. No statistical method was used to predetermine sample size but our sample sizes are similar to those reported in previous publications^7,73^. In the human prion challenge study, mice that met predetermined euthanasia criteria after AAV injection but before prion isolate inoculation were excluded from the study. No other data were excluded from the analyses. Experimental groups were assigned randomly and investigators performing injections and prion isolate inoculation were blinded from the experimental conditions.

### Reporting summary

Further information on research design is available in the Nature Portfolio Reporting Summary linked to this article.

## Online content

Any methods, additional references, Nature Portfolio reporting summaries, source data, extended data, supplementary information, acknowledgements, peer review information; details of author contributions and competing interests; and statements of data and code availability are available at 10.1038/s41591-024-03466-w.

## Supplementary information

Supplementary Information Supplementary Figs. 1–5, Sequences 1–11 and captions for Tables 1–8.

Reporting Summary

Supplementary Tables Supplementary Tables 1–8.

## Data Availability

There is no restriction on experimental data availability from this study. High-throughput DNA sequencing data files are deposited to the National Center for Biotechnology Information’s Sequence Read Archive (NCBI SRA) database under accession code PRJNA1178796. DNA sequences of the AAV vectors are provided in the Supplementary Sequences. GRCm38/mm10 (mouse) reference genome sequence was obtained from NCBI RefSeq assembly GCF_000001635.20. GRCh37/hg19 (human) reference genome sequence was obtained from NCBI RefSeq assembly GCF_000001405.13. Other raw data are deposited in the study’s online Git repository at https://github.com/ericminikel/base_editing.

## Code availability

Codes used to analyze the frequency of off-target editing are available in the study’s online Git repository at https://github.com/ericminikel/base_editing.
