# Author Correction: In vivo base editing extends lifespan of a humanized mouse model of prion disease

**DOI:** 10.1038/s41591-025-03540-x

**Published:** 2025-01-30

**Authors:** Meirui An, Jessie R. Davis, Jonathan M. Levy, Fiona E. Serack, John W. Harvey, Pamela P. Brauer, Catherine P. Pirtle, Kiara N. Berríos, Gregory A. Newby, Wei-Hsi Yeh, Nikita Kamath, Meredith Mortberg, Yuan Lian, Michael Howard, Kendrick DeSouza-Lenz, Kenia Guzman, Aaron Thai, Samantha Graffam, Vanessa Laversenne, Alissa A. Coffey, Jeannine Frei, Sarah E. Pierce, Jiri G. Safar, Benjamin E. Deverman, Eric Vallabh Minikel, Sonia M. Vallabh, David R. Liu

**Affiliations:** 1 Merkin Institute of Transformative Technologies in Healthcare, Broad Institute of MIT and Harvard, Cambridge, MA USA; 2 Department of Chemistry and Chemical Biology, Harvard University, Cambridge, MA USA; 3 Howard Hughes Medical Institute, Harvard University, Cambridge, MA USA; 4 Stanley Center for Psychiatric Research, Broad Institute of MIT and Harvard, Cambridge, MA USA; 5 Comparative Medicine, Broad Institute of MIT and Harvard, Cambridge, MA USA; 6 Case Western Reserve University, Cleveland, OH USA; 7 McCance Center for Brain Health and Department of Neurology, Massachusetts General Hospital, Boston, MA USA; 8 Department of Neurology, Harvard Medical School, Boston, MA USA; 9 Prion Alliance, Cambridge, MA USA

**Keywords:** Targeted gene repair, Prion diseases

Correction to: *Nature Medicine* 10.1038/s41591-024-03466-w, published online 14 January 2025.

In the version of the article initially published, Vanessa Laversenne (Stanley Center for Psychiatric Research, Broad Institute of MIT and Harvard, Cambridge, MA, USA) was missing from the author list. The error has been corrected in the HTML and PDF versions of the article.

