# Supplementary material for: In vivo base editing extends lifespan of a humanized mouse model of prion disease
Source: Nat Med. 2025 Jan 14;31(4):1319–28. doi: 10.1038/s41591-024-03466-w (PMC12003183; doi:10.1038/s41591-024-03466-w)
Supplement: Supplementary file 1 — Supplementary Figs. 1–5, Sequences 1–11 and captions for Tables 1–8. [file 41591_2024_3466_MOESM1_ESM.pdf]

nature medicine

Supplementary information https://doi.org/10.1038/s41591-024-03466-w

In vivo base editing extends lifespan of a humanized mouse model of prion disease

In the format provided by the authors and unedited

***In vivo* base editing extends lifespan of a humanized mouse model of prion disease**

Supplementary Information

Supplementary Figures

Supplementary Figure 1. Off-target editing in HEK293T cells after plasmid transfection of TadCBEd and *PRNP* R37X sgRNA.

Supplementary Figure 2. Off-target editing in mouse brain tissues harvested 35 days after treatment with dual-AAV PHP.eB BE3.9max and dual-AAV PHP.eB TadCBEd encoding *PRNP* R37X sgRNA.

Supplementary Figure 3. Off-target editing in mouse brain tissues harvested 100 days after treatment with dual-AAV PHP.eB BE3.9max and dual-AAV PHP.eB TadCBEd encoding *PRNP* R37X sgRNA.

Supplementary Figure 4. Off-target editing in mouse brain tissues harvested 600 days after treatment with dual-AAV PHP.eB BE3.9max encoding *PRNP* R37X sgRNA.

Supplementary Figure 5. Representative flow cytometry gating for the analysis of PrP levels in HEK293T cells.

Supplementary Sequences

Supplementary Sequence 1. Dual-AAV BE3.9max *PRNP* R37X sgRNA (N-terminus).

Supplementary Sequence 2. Dual-AAV BE3.9max *PRNP* R37X sgRNA (C-terminus).

Supplementary Sequence 3. Dual-AAV TadCBEd *PRNP* R37X F+E-sgRNA (N-terminus).

Supplementary Sequence 4. Dual-AAV TadCBEd *PRNP* R37X F+E-sgRNA (C-terminus).

Supplementary Sequence 5. Dual-AAV TadCBEd *PRNP* R37X F+E-sgRNA (N-terminus) with hSYN promoter, 3xmiR-183 and 3xmiR-122 target sites.

Supplementary Sequence 6. Dual-AAV TadCBEd *PRNP* R37X F+E-sgRNA (C-terminus) with hSYN promoter, 3xmiR-183 and 3xmiR-122 target sites.

Supplementary Sequence 7. Single-AAV SauriCas9-TadCBEd with *PRNP* R37X F-sgRNA.

Supplementary Sequence 8. Single-AAV enCjCas9-TadCBEd with *PRNP* Q91X F-sgRNA.

Supplementary Sequence 9. Dual-AAV SpCas9-ABE8e(V106W) *PRNP* M1V F+E-sgRNA (N-terminus).

Supplementary Sequence 10. Dual-AAV SpCas9-ABE8e(V106W) *PRNP* M1V F+E-sgRNA (C-terminus).

Supplementary Sequence 11. Single-AAV SauriCas9-ABE8e with *PRNP* M1V F-sgRNA.

Supplementary Tables (provided as a separate file):

Supplementary Table 1. Body weight of Tg25109 mice in the human pathogenic prion inoculation study.

Supplementary Table 2. Nest scores of Tg25109 mice in the human pathogenic prion inoculation study.

Supplementary Table 3. Body weight of Tg25109 mice 3 days and 7 days after dual-AAV PHP.eB BE3.9max or TadCBEd treatment at 1.5×10^{13} vg/kg dose.

Supplementary Table 4. Sequences of 299 CIRCLE-seq-nominated off-target sites in the human genome.

Supplementary Table 5. Sequences of 197 CIRCLE-seq-nominated off-target sites in the mouse genome.

Supplementary Table 6. Sequences of sgRNAs evaluated in the study.

Supplementary Table 7. Sequences of primers used for RT-qPCR.

Supplementary Table 8. Mouse ID, sex, experimental condition and results.

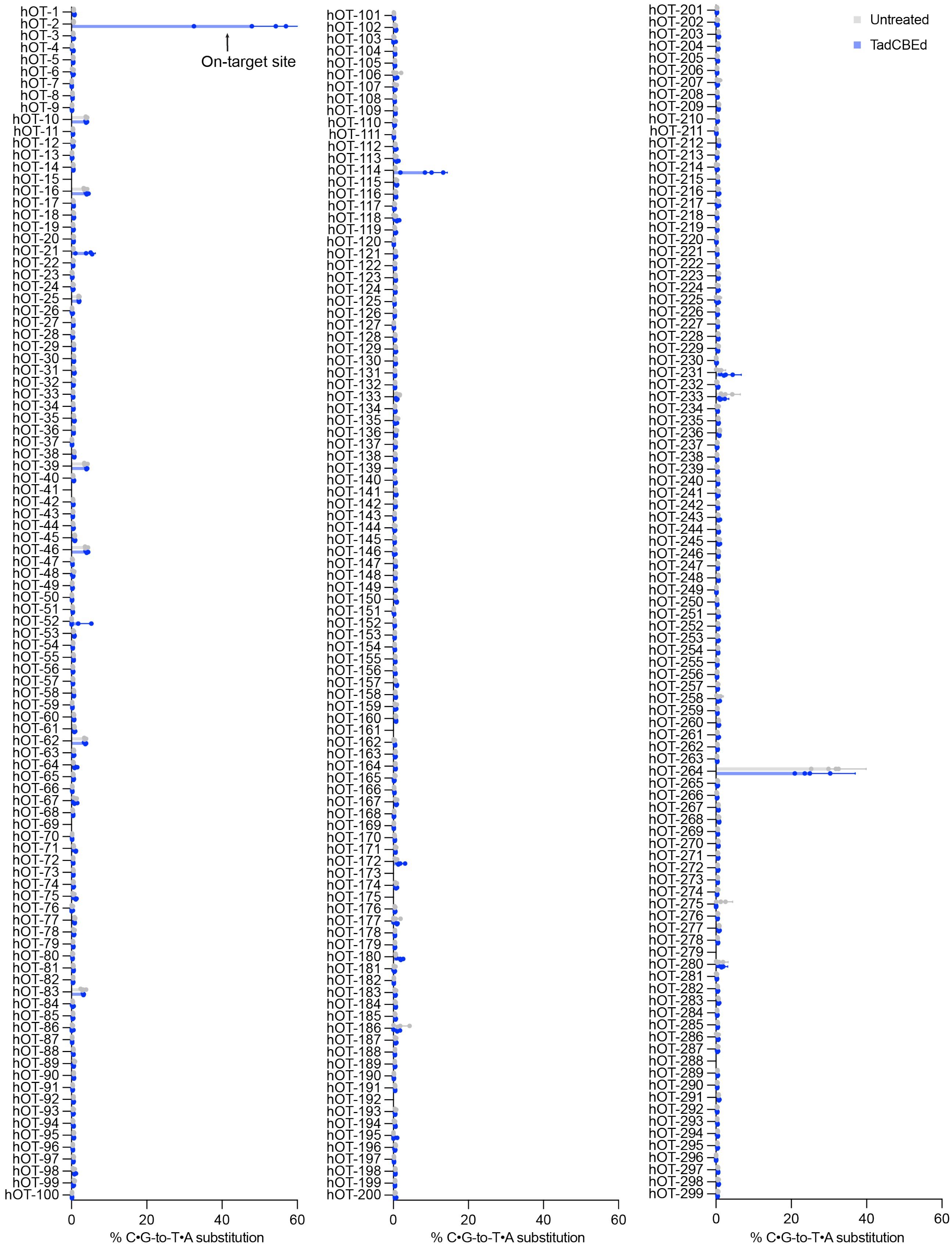
Untreated
TadCBEd
On-target site
% C•G-to-T•A substitution
% C•G-to-T•A substitution
% C•G-to-T•A substitution

Supplementary Fig. 1 | Off-target editing in HEK293T cells after plasmid transfection of TadCBEd and *PRNP* R37X sgRNA. The percentage of C•G-to-T•A substitution at the 299 CIRCLE-seq nominated off-target sites in the human genome (GRCh37) is shown; see Fig. 5a. Genomic DNA was extracted from HEK293T cells untreated (n=3) or after 3 days following transfection of plasmids encoding TadCBEd and the *PRNP* R37X sgRNA (n=3). Dots represent individual biological replicates and error bars represent 95% CI.

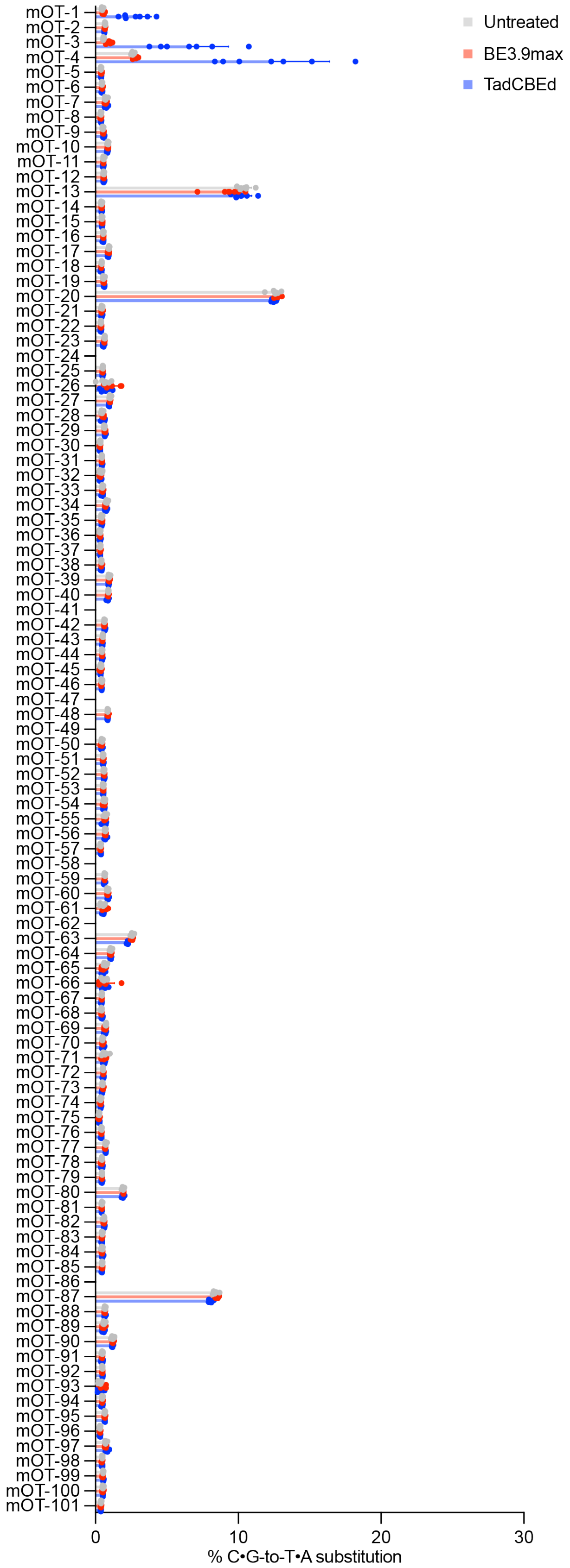

Supplementary Fig. 2 | Off-target editing in mouse brain tissues harvested 35 days after treatment with dual-AAV PHP.eB BE3.9max and dual-AAV PHP.eB TadCBEd

encoding *PRNP* R37X sgRNA. The percentage of C•G-to-T•A substitution at the top 100 CIRCLE-seq-nominated off-target sites in the mouse genome (GRCm38) is shown; see Fig. 5b. Genomic DNA was extracted from bulk brain hemispheres of Tg25109 mice untreated (n=6), or 35 days after treatment with dual-AAV PHP.eB BE3.9max with *PRNP* R37X sgRNA (n=6), or 35 days after treatment with dual-AAV PHP.eB TadCBEd with *PRNP* R37X sgRNA (n=6) at a total dose of 1.5×10^{13} vg/kg. Dots represent individual biological replicates and error bars represent 95% CI.

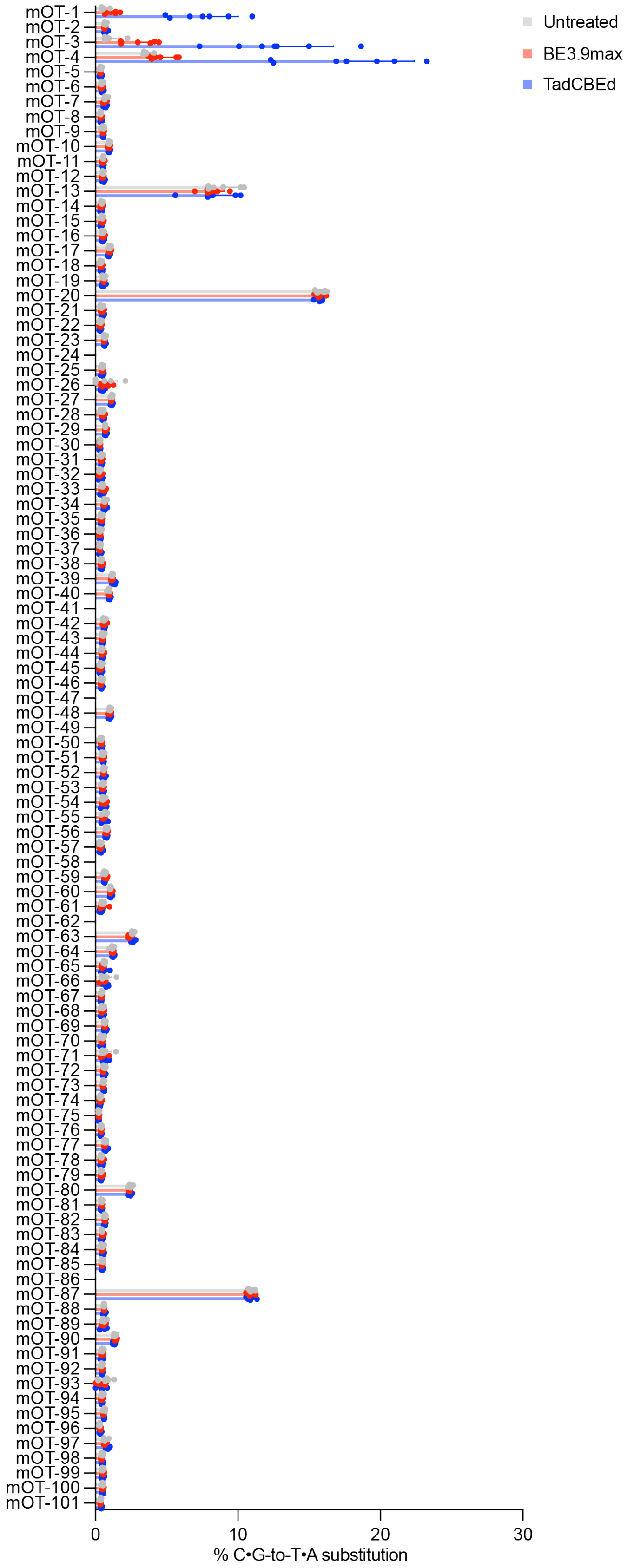

Supplementary Fig. 3 | Off-target editing in mouse brain tissues harvested 100 days after treatment with dual-AAV PHP.eB BE3.9max and dual-AAV PHP.eB TadCBEd

encoding *PRNP* R37X sgRNA. The percentage of C•G-to-T•A substitution at the top 100 CIRCLE-seq-nominated off-target sites in the mouse genome (GRCm38) is shown; see Fig. 5c. Genomic DNA was extracted from bulk brain hemispheres of Tg25109 mice untreated (n=6), or 100 days after treatment with dual-AAV PHP.eB BE3.9max with *PRNP* R37X sgRNA (n=6), or 100 days after treatment with dual-AAV PHP.eB TadCBEd with *PRNP* R37X sgRNA (n=6) at a total dose of $1.5x10^{13}$ vg/kg. Dots represent individual biological replicates and error bars represent 95% CI.

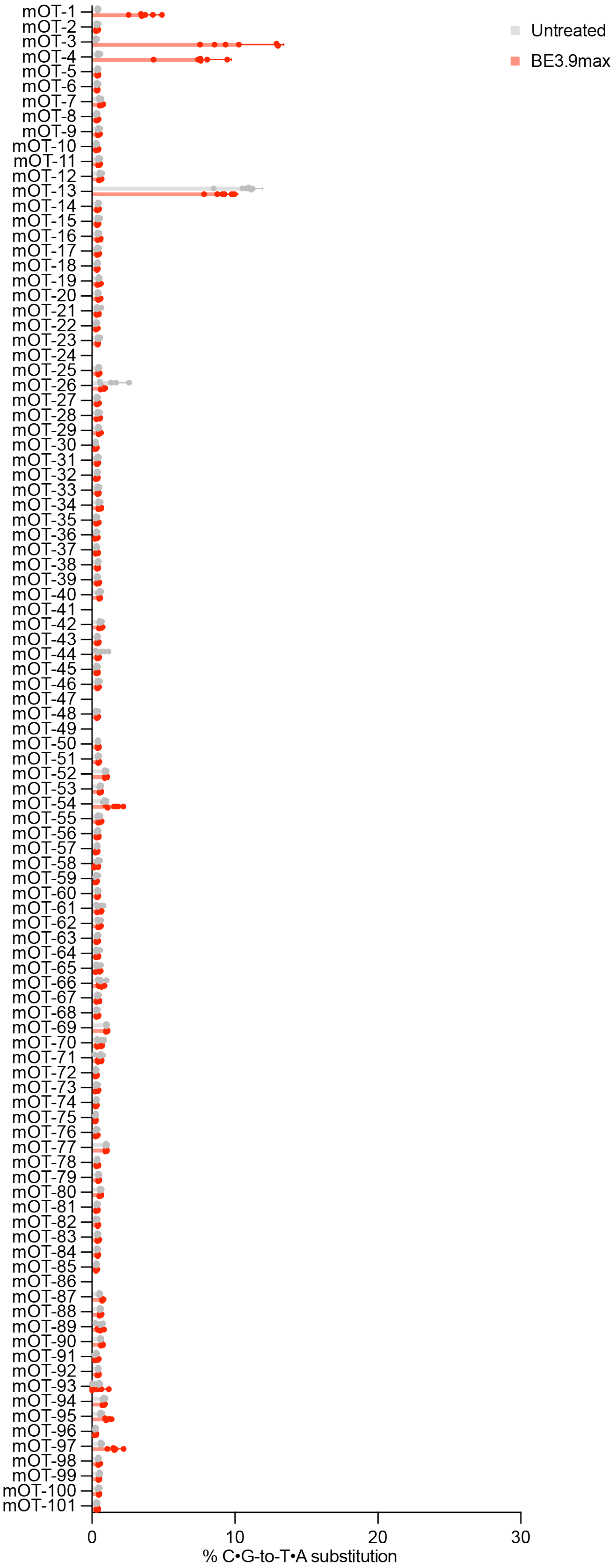

Supplementary Fig. 4 | Off-target editing in mouse brain tissues harvested 600 days after treatment with dual-AAV PHP.eB BE3.9max encoding *PRNP* R37X sgRNA. The

percentage of C•G-to-T•A substitution at the top 100 CIRCLE-seq-nominated off-target sites in the mouse genome (GRCm38) is shown; see Fig. 5d. Genomic DNA was extracted from bulk brain hemispheres of Tg25109 mice untreated (n=5), or 600 days after treatment with dual-AAV PHP.eB BE3.9max with *PRNP* R37X sgRNA (n=5) at a total dose of $1x10^{14}$ vg/kg. Dots represent individual biological replicates and error bars represent 95% CI.

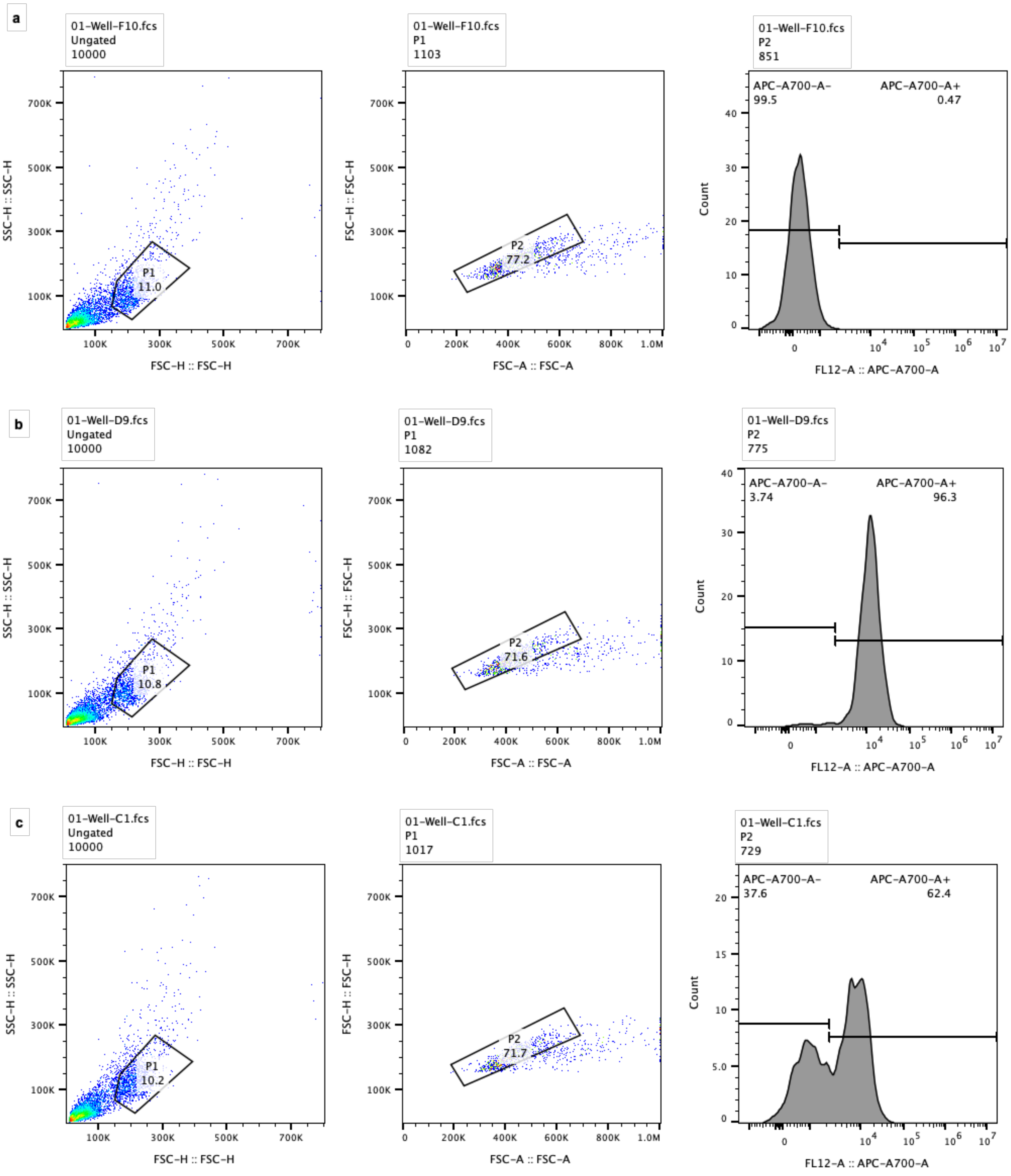

Supplementary Fig. 5 | Representative flow cytometry gating for the analysis of PrP levels in HEK293T cells. a and b, Representative flow cytometry gating plot for untreated HEK293T cells either without (a) or with (b) incubation using a fluorescently conjugated 6D11 antibody that binds PrP. c, Representative flow cytometry gating plot for BE4max-treated HEK293T cells incubated with 6D11 antibodies. Individual cells were gated based on forward scatter-height (FSC-H) and side scatter-height (SSC-H) ratios, as well as on forward scatter-area (FSC-A) and forward scatter-height (FSC-H) ratios. Cells were gated based on the intensity of the allophycocyanin (APC) signal.

Supplementary Sequence 1. Dual-AAV BE3.9max *PRNP* R37X sgRNA (N-terminus).

ITR-Cbh promoter-NLS-APOBEC deaminase domain-linker-SpCas9 (amino acids 1-572)-NpuN-NLS-WPRE-bovine growth hormone(bGH)-derived poly(A)-*PRNP* R37X sgRNA (reverse complement)-human U6 promoter (reverse complement)-ITR

CTGCGCGCTCGCTCGCTCACTGAGGCCGCCCGGGCAAAGCCCGGGCGTCGGGCGACC
TTTGGTCGCCCGGCCTCAGTGAGCGAGCGAGCGCGCAGAGAGGGAGTGGCCAACTCC
ATCACTAGGGGTTCCTGCGGCCTCTAGATCAGGGTACCCGTTACATAACTTACGGTAAAT
GGCCCGCCTGGCTGACCGCCCAACGACCCCCGCCCATTGACGTCAATAGTAACGCCAAT
AGGGACTTTCCATTGACGTCAATGGGTGGAGTATTTACGGTAAACTGCCCACTTGGCAGT
ACATCAAGTGTATCATATGCCAAGTACGCCCCCTATTGACGTCAATGACGGTAAATGGCCC
GCCTGGCATTGTGCCCAGTACATGACCTTATGGGACTTTCCTACTTGGCAGTACATCTAC
GTATTAGTCATCGCTATTACCATGGTCGAGGTGAGCCCCACGTTCTGCTTCACTCTCCCC
ATCTCCCCCCCCTCCCCACCCCCAATTTTGTATTTATTTATTTTTTAATTATTTTGTGCAGCG
ATGGGGGCGGGGGGGGGGGGGGGGCGCGCGCCAGGCGGGGCGGGGCGGGGCGAG
GGGCGGGGCGGGGCGAGGCGGAGAGGTGCGGCGGCAGCCAATCAGAGCGGCGCGCT
CCGAAAGTTTCCTTTTATGGCGAGGCGGCGGCGGCGGCGGCCCTATAAAAAGCGAAGC
GCGCGGCGGGCGGGAGTCGCTGCGACGCTGCCTTCGCCCCGTGCCCCGCTCCGCCG
CCGCCTCGCGCCGCCCGCCCCGGCTCTGACTGACCGCGTTACTCCCACAGGTGAGCG
GGCGGGACGGCCCTTCTCCTCCGGGCTGTAATTAGCTGAGCAAGAGGTAAGGGTTTAAG
GGATGGTTGGTTGGTGGGGTATTAATGTTTAATTACCTGGAGCACCTGCCTGAAATCACTT
TTTTTCAGGTTGGACCGGTGCCACCATGAAACGGACAGCCGACGGAAGCGAGTTCGAG
TCACCAAAGAAGAAGCGGAAAGTCTCCTCAGAGACTGGGCCTGTCGCCGTCGATCCAA
CCCTGCGCCGCCGGATTGAACCTCACGAGTTTGAAGTGTTCTTTGACCCCCGGGAGCT
GAGAAAGGAGACATGCCTGCTGTACGAGATCAACTGGGGAGGCAGGCACTCCATCTGG
AGGCACACCTCTCAGAACACAAATAAGCACGTGGAGGTGAACTTCATCGAGAAGTTTACC
ACAGAGCGGTACTTCTGCCCCAATACCAGATGTAGCATCACATGGTTTCTGAGCTGGTCC
CCTTGCGGAGAGTGTAGCAGGGCCATCACCGAGTTCCTGTCCAGATATCCACACGTGAC
ACTGTTTATCTACATCGCCAGGCTGTATCACCACGCAGACCCAAGGAATAGGCAGGGCCT
GCGCGATCTGATCAGCTCCGGCGTGACCATCCAGATCATGACAGAGCAGGAGTCCGGC
TACTGCTGGCGGAACTTCGTGAATTATTCTCCTAGCAACGAGGCCCACTGGCCTAGGTAC
CCACACCTGTGGGTGCGCCTGTACGTGCTGGAGCTGTATTGCATCATCCTGGGCCTGCC
CCCTTGTCTGAATATCCTGCGGAGAAAGCAGCCCCAGCTGACCTTCTTTACAATCGCCCT
GCAGTCTTGTCACTATCAGAGGCTGCCACCCCACATCCTGTGGGCCACAGGCCTGAAGT
CTGGAGGATCTAGCGGAGGATCCTCTGGCAGCGAGACACCAGGAACAAGCGAGTCAGC
AACACCAGAGAGCAGTGGCGGCAGCAGCGGCGGCAGCGACAAGAAGTACAGCATCGG
CCTGGCCATCGGCACCAACTCTGTGGGCTGGGCCGTGATCACCGACGAGTACAAGGTG
CCCAGCAAGAAATTCAAGGTGCTGGGCAACACCGACCGGCACAGCATCAAGAAGAACC
TGATCGGAGCCCTGCTGTTCGACAGCGGCGAAACAGCCGAGGCCACCCGGCTGAAGA
GAACCGCCAGAAGAAGATACACCAGACGGAAGAACCGGATCTGCTATCTGCAAGAGATC
TTCAGCAACGAGATGGCCAAGGTGGACGACAGCTTCTTCCACAGACTGGAAGAGTCCTT
CCTGGTGGAAGAGGATAAGAAGCACGAGCGGCACCCCATCTTCGGCAACATCGTGGAC

GAGGTGGCCTACCACGAGAAGTACCCCACCATCTACCACCTGAGAAAGAAACTGGTGGA
CAGCACCGACAAGGCCGACCTGCGGCTGATCTATCTGGCCCTGGCCCACATGATCAAGT
TCCGGGGCCACTTCCTGATCGAGGGCGACCTGAACCCCGACAACAGCGACGTGGACAA
GCTGTTCATCCAGCTGGTGCAGACCTACAACCAGCTGTTCGAGGAAAACCCCATCAACG
CCAGCGGCGTGGACGCCAAGGCCATCCTGTCTGCCAGACTGAGCAAGAGCAGACGGCT
GGAAAATCTGATCGCCCAGCTGCCCGGCGAGAAGAAGAATGGCCTGTTCGGAAACCTG
ATTGCCCTGAGCCTGGGCCTGACCCCCAACTTCAAGAGCAACTTCGACCTGGCCGAGG
ATGCCAAACTGCAGCTGAGCAAGGACACCTACGACGACGACCTGGACAACCTGCTGGC
CCAGATCGGCGACCAGTACGCCGACCTGTTTCTGGCCGCCAAGAACCTGTCCGACGCC
ATCCTGCTGAGCGACATCCTGAGAGTGAACACCGAGATCACCAAGGCCCCCCTGAGCG
CCTCTATGATCAAGAGATACGACGAGCACCACCAGGACCTGACCCTGCTGAAAGCTCTC
GTGCGGCAGCAGCTGCCTGAGAAGTACAAAGAGATTTTCTTCGACCAGAGCAAGAACGG
CTACGCCGGCTACATTGACGGCGGAGCCAGCCAGGAAGAGTTCTACAAGTTCATCAAGC
CCATCCTGGAAAAGATGGACGGCACCGAGGAACTGCTCGTGAAGCTGAACAGAGAGGA
CCTGCTGCGGAAGCAGCGGACCTTCGACAACGGCAGCATCCCCCACCAGATCCACCTG
GGAGAGCTGCACGCCATTCTGCGGCGGCAGGAAGATTTTTACCCATTCCTGAAGGACAA
CCGGGAAAAGATCGAGAAGATCCTGACCTTCCGCATCCCCTACTACGTGGGCCCTCTGG
CCAGGGGAAACAGCAGATTCGCCTGGATGACCAGAAAGAGCGAGGAAACCATCACCCC
CTGGAACTTCGAGGAAGTGGTGGACAAGGGCGCTTCCGCCCAGAGCTTCATCGAGCGG
ATGACCAACTTCGATAAGAACCTGCCCAACGAGAAGGTGCTGCCCAAGCACAGCCTGCT
GTACGAGTACTTCACCGTGTATAACGAGCTGACCAAAGTGAAATACGTGACCGAGGGAAT
GAGAAAGCCCGCCTTCCTGAGCGGCGAGCAGAAAAAGGCCATCGTGGACCTGCTGTTC
AAGACCAACCGGAAAGTGACCGTGAAGCAGCTGAAAGAGGACTACTTCAAGAAAATCGA
GTGCCTGTCCTACGAGACAGAGATCCTGACAGTGGAGTATGGCCTGCTGCCAATCGGCA
AGATCGTGGAGAAGAGGATCGAGTGTACCGTGTACTCTGTGGATAACAATGGCAACATCT
ATACACAGCCCGTGGCACAGTGGCACGATAGGGGAGAGCAGGAGGTGTTCGAGTATTG
CCTGGAGGACGGCAGCCTGATCAGGGCAACCAAGGACCACAAGTTCATGACAGTGGAT
GGCCAGATGCTGCCCATCGACGAGATTTTCGAGCGGGAGCTGGACCTGATGAGAGTGG
ATAACCTGCCTAATAGCGGAGGCAGTAAAAGAACAGCAGACGGGAGTGAGTTTGAGCCC
AAGAAAAAGAGAAAGGTGTAAGATCTGATAATCAACCTCTGGATTACAAAATTTGTGAAAG
ATTGACTGGTATTCTTAACTATGTTGCTCCTTTTACGCTATGTGGATACGCTGCTTTAATGC
CTTTGTATCATGCTATTGCTTCCCGTATGGCTTTCATTTTCTCCTCCTTGTATAAATCCTGGT
TAGTTCTTGCCACGGCGGAACTCATCGCCGCCTGCCTTGCCCGCTGCTGGACAGGGGC
TCGGCTGTTGGGCACTGACAATTCCGTGGTGCGACTGTGCCTTCTAGTTGCCAGCCATC
TGTTGTTTGCCCCTCCCCCGTGCCTTCCTTGACCCTGGAAGGTGCCACTCCCACTGTCC
TTTCCTAATAAAATGAGGAAATTGCATCGCATTGTCTGAGTAGGTGTCATTCTATTCTGGG
GGGTGGGGTGGGGCAGGACAGCAAGGGGGAGGATTGGGAAGACAATAGCAGGCATGC
TGGGGATGCGGTGGGCTCTATGGCTCGAGAAAAAAAGCACCGACTCGGTGCCACTTTTT
CAAGTTGATAACGGACTAGCCTTATTTTAACTTGCTATTTCTAGCTCTAAAACTGCCCCGG
GTATCGGCTGCCGGTGTTTCGTCCTTTCCACAAGATATATAAAGCCAAGAAATCGAAATAC
TTTCAAGTTACGGTAAGCATATGATAGTCCATTTTAAAACATAATTTTAAAACTGCAAACTAC
CCAAGAAATTATTACTTTCTACGTCACGTATTTTGTACTAATATCTTTGTGTTTACAGTCAAA

TTAATTCTAATTATCTCTCTAACAGCCTTGTATCGTATATGCAAATATGAAGGAATCATGGGA
AATAGGCCCTCTTCCTGCCCGACCTTGCGGCCGCAGGAACCCCTAGTGATGGAGTTGGC
CACTCCCTCTCTGCGCGCTCGCTCGCTCACTGAGGCCGGGCGACCAAAGGTCGCCCGA
CGCCCGGGCTTTGCCCGGGCGGCCTCAGTGAGCGAGCGAGCGCGCAG

Supplementary Sequence 2. Dual-AAV BE3.9max *PRNP* R37X sgRNA (C-terminus).

ITR-Cbh promoter-NLS-NpuC-SpCas9 (amino acids 573-1367)-UGI-NLS-WPRE-bovine growth hormone(bGH)-derived poly(A)-*PRNP* R37X sgRNA (reverse complement)-human U6 promoter (reverse complement)-ITR

CTGCGCGCTCGCTCGCTCACTGAGGCCGCCCGGGCAAAGCCCGGGCGTCGGGCGACC
TTTGGTCGCCCGGCCTCAGTGAGCGAGCGAGCGCGCAGAGAGGGAGTGGCCAACTCC
ATCACTAGGGGTTCCTGCGGCCTCTAGATCAGGGTACCCGTTACATAACTTACGGTAAAT
GGCCCGCCTGGCTGACCGCCCAACGACCCCCGCCCATTGACGTCAATAGTAACGCCAAT
AGGGACTTTCCATTGACGTCAATGGGTGGAGTATTTACGGTAAACTGCCCACTTGGCAGT
ACATCAAGTGTATCATATGCCAAGTACGCCCCCTATTGACGTCAATGACGGTAAATGGCCC
GCCTGGCATTGTGCCCAGTACATGACCTTATGGGACTTTCCTACTTGGCAGTACATCTAC
GTATTAGTCATCGCTATTACCATGGTCGAGGTGAGCCCCACGTTCTGCTTCACTCTCCCC
ATCTCCCCCCCCTCCCCACCCCCAATTTTGTATTTATTTATTTTTTAATTATTTTGTGCAGCG
ATGGGGGCGGGGGGGGGGGGGGGCGCGCGCCAGGCGGGGCGGGGCGGGGCGAG
GGGCGGGGCGGGGCGAGGCGGAGAGGTGCGGCGGCAGCCAATCAGAGCGGCGCGCT
CCGAAAGTTTCCTTTTATGGCGAGGCGGCGGCGGCGGCGGCCCTATAAAAAGCGAAGC
GCGCGGCGGGCGGGAGTCGCTGCGACGCTGCCTTCGCCCCGTGCCCCGCTCCGCCG
CCGCCTCGCGCCGCCCGCCCCGGCTCTGACTGACCGCGTTACTCCCACAGGTGAGCG
GGCGGGACGGCCCTTCTCCTCCGGGCTGTAATTAGCTGAGCAAGAGGTAAGGGTTTAAG
GGATGGTTGGTTGGTGGGGTATTAATGTTTAATTACCTGGAGCACCTGCCTGAAATCACTT
TTTTTCAGGTTGGACCGGTGCCACCATGAAACGGACAGCCGACGGAAGCGAGTTCGAG
TCACCAAAGAAGAAGCGGAAAGTCATCAAGATTGCTACACGGAAATACCTGGGAAAGCA
GAACGTGTACGACATCGGCGTGGAGCGGGATCACAACTTCGCCCTGAAGAATGGCTTTA
TCGCCAGCAATTGCTTCGACTCCGTGGAAATCTCCGGCGTGGAAGATCGGTTCAACGCC
TCCCTGGGCACATACCACGATCTGCTGAAAATTATCAAGGACAAGGACTTCCTGGACAAT
GAGGAAAACGAGGACATTCTGGAAGATATCGTGCTGACCCTGACACTGTTTGAGGACAG
AGAGATGATCGAGGAACGGCTGAAAACCTATGCCCACCTGTTCGACGACAAAGTGATGA
AGCAGCTGAAGCGGCGGAGATACACCGGCTGGGGCAGGCTGAGCCGGAAGCTGATCA
ACGGCATCCGGGACAAGCAGTCCGGCAAGACAATCCTGGATTTCCTGAAGTCCGACGG
CTTCGCCAACAGAAACTTCATGCAGCTGATCCACGACGACAGCCTGACCTTTAAAGAGG
ACATCCAGAAAGCCCAGGTGTCCGGCCAGGGCGATAGCCTGCACGAGCACATTGCCAAT
CTGGCCGGCAGCCCCGCCATTAAGAAGGGCATCCTGCAGACAGTGAAGGTGGTGGACG
AGCTCGTGAAAGTGATGGGCCGGCACAAGCCCGAGAACATCGTGATCGAAATGGCCAG
AGAGAACCAGACCACCCAGAAGGGACAGAAGAACAGCCGCGAGAGAATGAAGCGGATC
GAAGAGGGCATCAAAGAGCTGGGCAGCCAGATCCTGAAAGAACACCCCGTGGAAAACA
CCCAGCTGCAGAACGAGAAGCTGTACCTGTACTACCTGCAGAATGGGCGGGATATGTAC
GTGGACCAGGAACTGGACATCAACCGGCTGTCCGACTACGATGTGGACCATATCGTGCC
TCAGAGCTTTCTGAAGGACGACTCCATCGACAACAAGGTGCTGACCAGAAGCGACAAGA
ACCGGGGCAAGAGCGACAACGTGCCCTCCGAAGAGGTCGTGAAGAAGATGAAGAACTA
CTGGCGGCAGCTGCTGAACGCCAAGCTGATTACCCAGAGAAAGTTCGACAATCTGACCA
AGGCCGAGAGAGGCGGCCTGAGCGAACTGGATAAGGCCGGCTTCATCAAGAGACAGCT
GGTGGAAACCCGGCAGATCACAAAGCACGTGGCACAGATCCTGGACTCCCGGATGAAC
ACTAAGTACGACGAGAATGACAAGCTGATCCGGGAAGTGAAAGTGATCACCCTGAAGTC
CAAGCTGGTGTCCGATTTCCGGAAGGATTTCCAGTTTTACAAAGTGCGCGAGATCAACAA
CTACCACCACGCCCACGACGCCTACCTGAACGCCGTCGTGGGAACCGCCCTGATCAAA
AAGTACCCTAAGCTGGAAAGCGAGTTCGTGTACGGCGACTACAAGGTGTACGACGTGCG

GAAGATGATCGCCAAGAGCGAGCAGGAAATCGGCAAGGCTACCGCCAAGTACTTCTTCT
ACAGCAACATCATGAACTTTTTCAAGACCGAGATTACCCTGGCCAACGGCGAGATCCGG
AAGCGGCCTCTGATCGAGACAAACGGCGAAACCGGGGAGATCGTGTGGGATAAGGGCC
GGGATTTTGCCACCGTGCGGAAAGTGCTGAGCATGCCCCAAGTGAATATCGTGAAAAAG
ACCGAGGTGCAGACAGGCGGCTTCAGCAAAGAGTCTATCCTGCCCAAGAGGAACAGCG
ATAAGCTGATCGCCAGAAAGAAGGACTGGGACCCTAAGAAGTACGGCGGCTTCGACAGC
CCCACCGTGGCCTATTCTGTGCTGGTGGTGGCCAAAGTGGAAAAGGGCAAGTCCAAGA
AACTGAAGAGTGTGAAAGAGCTGCTGGGGATCACCATCATGGAAAGAAGCAGCTTCGAG
AAGAATCCCATCGACTTTCTGGAAGCCAAGGGCTACAAAGAAGTGAAAAAGGACCTGAT
CATCAAGCTGCCTAAGTACTCCCTGTTCGAGCTGGAAAACGGCCGGAAGAGAATGCTGG
CCTCTGCCGGCGAACTGCAGAAGGGAAACGAACTGGCCCTGCCCTCCAAATATGTGAAC
TTCCTGTACCTGGCCAGCCACTATGAGAAGCTGAAGGGCTCCCCCGAGGATAATGAGCA
GAAACAGCTGTTTGTGGAACAGCACAAGCACTACCTGGACGAGATCATCGAGCAGATCA
GCGAGTTCTCCAAGAGAGTGATCCTGGCCGACGCTAATCTGGACAAAGTGCTGTCCGCC
TACAACAAGCACCGGGATAAGCCCATCAGAGAGCAGGCCGAGAATATCATCCACCTGTTT
ACCCTGACCAATCTGGGAGCCCCTGCCGCCTTCAAGTACTTTGACACCACCATCGACCG
GAAGAGGTACACCAGCACCAAAGAGGTGCTGGACGCCACCCTGATCCACCAGAGCATC
ACCGGCCTGTACGAGACACGGATCGACCTGTCTCAGCTGGGAGGTGACAGCGGCGGG
AGCGGCGGGAGCGGGGGGAGCACTAATCTGAGCGACATCATTGAGAAGGAGACTGGGA
AACAGCTGGTCATTCAGGAGTCCATCCTGATGCTGCCTGAGGAGGTGGAGGAAGTGATC
GGCAACAAGCCAGAGTCTGACATCCTGGTGCACACCGCCTACGACGAGTCCACAGATGA
GAATGTGATGCTGCTGACCTCTGACGCCCCCGAGTATAAGCCTTGGGCCCTGGTCATCC
AGGATTCTAACGGCGAGAATAAGATCAAGATGCTGAGCGGAGGATCCAAAAGAACCGCC
GACGGCAGCGAATTCGAGCCCAAGAAGAAGAGGAAAGTCTAAGATCTGATAATCAACCT
CTGGATTACAAAATTTGTGAAAGATTGACTGGTATTCTTAACTATGTTGCTCCTTTTACGCT
ATGTGGATACGCTGCTTTAATGCCTTTGTATCATGCTATTGCTTCCCGTATGGCTTTCATTT
TCTCCTCCTTGTATAAATCCTGGTTAGTTCTTGCCACGGCGGAACTCATCGCCGCCTGCC
TTGCCCGCTGCTGGACAGGGGCTCGGCTGTTGGGCACTGACAATTCCGTGGTGCGACT
GTGCCTTCTAGTTGCCAGCCATCTGTTGTTTGCCCCTCCCCCGTGCCTTCCTTGACCCT
GGAAGGTGCCACTCCCACTGTCCTTTCCTAATAAAATGAGGAAATTGCATCGCATTGTCT
GAGTAGGTGTCATTCTATTCTGGGGGGTGGGGTGGGGCAGGACAGCAAGGGGGAGGAT
TGGGAAGACAATAGCAGGCATGCTGGGGATGCGGTGGGCTCTATGGCTCGAGAAAAAAA
GCACCGACTCGGTGCCACTTTTTCAAGTTGATAACGGACTAGCCTTATTTTAACTTGCTAT
TTCTAGCTCTAAAACTGCCCCGGGTATCGGCTGCCGGTGTTTCGTCCTTTCCACAAGATA
TATAAAGCCAAGAAATCGAAATACTTTCAAGTTACGGTAAGCATATGATAGTCCATTTTAAAA
CATAATTTTAAAACTGCAAACTACCCAAGAAATTATTACTTTCTACGTCACGTATTTTGTACT
AATATCTTTGTGTTTACAGTCAAATTAATTCTAATTATCTCTCTAACAGCCTTGTATCGTATAT
GCAAATATGAAGGAATCATGGGAAATAGGCCCTCTTCCTGCCCGACCTTGCGGCCGCAG
GAACCCCTAGTGATGGAGTTGGCCACTCCCTCTCTGCGCGCTCGCTCGCTCACTGAGG
CCGGGCGACCAAAGGTCGCCCGACGCCCGGGCTTTGCCCGGGCGGCCTCAGTGAGCG
AGCGAGCGCGCAG

Supplementary Sequence 3. Dual-AAV TadCBEd *PRNP* R37X F+E-sgRNA (N-terminus).

ITR-Cbh promoter-NLS-TadCBEd deaminase domain-linker-SpCas9 (amino acids 1-572)-NpuN-NLS-WPRE-bovine growth hormone(bGH)-derived poly(A)-*PRNP* R37X F+E-sgRNA (reverse complement)-human U6 promoter (reverse complement)-ITR

CTGCGCGCTCGCTCGCTCACTGAGGCCGCCCGGGCAAAGCCCGGGCGTCGGCGACC
TTTGGTCGCCCGGCCTCAGTGAGCGAGCGAGCGCGCAGAGAGGGAGTGGCCAACTCC
ATCACTAGGGGTTCCTGCGGCCTCTAGATCAGGGTACCCGTTACATAACTTACGGTAAAT
GGCCCGCCTGGCTGACCGCCCAACGACCCCCGCCCATTGACGTCAATAGTAACGCCAAT
AGGGACTTTCCATTGACGTCAATGGGTGGAGTATTTACGGTAAACTGCCCACTTGGCAGT
ACATCAAGTGTATCATATGCCAAGTACGCCCCCTATTGACGTCAATGACGGTAAATGGCCC
GCCTGGCATTGTGCCCAGTACATGACCTTATGGGACTTTCCTACTTGGCAGTACATCTAC
GTATTAGTCATCGCTATTACCATGGTCGAGGTGAGCCCCACGTTCTGCTTCACTCTCCCC
ATCTCCCCCCCCTCCCCACCCCCAATTTTGTATTTATTTATTTTTTAATTATTTTGTGCAGCG
ATGGGGGCGGGGGGGGGGGGGGCGCGCGCCAGGCGGGGCGGGGCGGGGCGAG
GGGCGGGGCGGGGCGAGGCGGAGAGGTGCGGCGGCAGCCAATCAGAGCGGCGCGCT
CCGAAAGTTTCCTTTTATGGCGAGGCGGCGGCGGCGGCGGCCCTATAAAAAGCGAAGC
GCGCGGCGGGCGGGAGTCGCTGCGACGCTGCCTTCGCCCCGTGCCCCGCTCCGCCG
CCGCCTCGCGCCGCCCGCCCCGGCTCTGACTGACCGCGTTACTCCCACAGGTGAGCG
GGCGGGACGGCCCTTCTCCTCCGGGCTGTAATTAGCTGAGCAAGAGGTAAGGGTTTAAG
GGATGGTTGGTTGGTGGGGTATTAATGTTTAATTACCTGGAGCACCTGCCTGAAATCACTT
TTTTTCAGGTTGGACCGGTGCCACCATGAAACGGACAGCCGACGGAAGCGAGTTCGAG
TCACCAAAGAAGAAGCGGAAAGTCAGTTCTGAGGTGGAGTTTTCCCACGAGTACTGGAT
GAGACATGCCCTGACCCTGGCCAAGAGGGCACGGGATGAGAGGAAGGCGCCTGTGGG
AGCCGTGCTGGTGCTGAACAATAGAGTGATCGGCGAGGGCTGGAACAGAGCCATCGGC
CTGCACGACCCAACAGCCCATGCCGAAATTATAGCCCTGAGACAGGGCGGCCTGGTCAT
GCAGAACTACAGACTGATTGACGCCACCCTGTACGTGACATTCGAGCCTTGCGTGATGT
GCGCCGGCGCCATGATCAACTCTAGGATCGGCCGCGTGGTGTTTGGCGTGAGGAACTC
AAAAAGAGGCGCCGCAGGCTCCCTGATGAACGTGCTGAACTACCCCGGAATGAATCACC
GCGTCGAAATTACCGAGGGAATCCTGGCAGATGAATGTGCCGCCCTGCTGTGCGATTTC
TATCGGATGCCTAGACAGGTGTTCAATGCTCAGAAGAAGGCCCAGAGCTCCATCAACTCT
GGAGGATCTAGCGGAGGATCCTCTGGCAGCGAGACACCAGGAACAAGCGAGTCAGCAA
CACCAGAGAGCAGTGGCGGCAGCAGCGGCGGCAGCGACAAGAAGTACAGCATCGGCC

TGGCCATCGGCACCAACTCTGTGGGCTGGGCCGTGATCACCGACGAGTACAAGGTGCC
CAGCAAGAAATTCAAGGTGCTGGGCAACACCGACCGGCACAGCATCAAGAAGAACCTG
ATCGGAGCCCTGCTGTTCGACAGCGGCGAAACAGCCGAGGCCACCCGGCTGAAGAGAA
CCGCCAGAAGAAGATACACCAGACGGAAGAACCGGATCTGCTATCTGCAAGAGATCTTC
AGCAACGAGATGGCCAAGGTGGACGACAGCTTCTTCCACAGACTGGAAGAGTCCTTCCT
GGTGGAAGAGGATAAGAAGCACGAGCGGCACCCCATCTTCGGCAACATCGTGGACGAG
GTGGCCTACCACGAGAAGTACCCCACCATCTACCACCTGAGAAAGAAACTGGTGGACAG
CACCGACAAGGCCGACCTGCGGCTGATCTATCTGGCCCTGGCCCACATGATCAAGTTCC
GGGGCCACTTCCTGATCGAGGGCGACCTGAACCCCGACAACAGCGACGTGGACAAGCT
GTTCATCCAGCTGGTGCAGACCTACAACCAGCTGTTCGAGGAAAACCCCATCAACGCCA
GCGGCGTGGACGCCAAGGCCATCCTGTCTGCCAGACTGAGCAAGAGCAGACGGCTGG
AAAATCTGATCGCCCAGCTGCCCGGCGAGAAGAAGAATGGCCTGTTCGGAAACCTGATT
GCCCTGAGCCTGGGCCTGACCCCCAACTTCAAGAGCAACTTCGACCTGGCCGAGGATG
CCAAACTGCAGCTGAGCAAGGACACCTACGACGACGACCTGGACAACCTGCTGGCCCA
GATCGGCGACCAGTACGCCGACCTGTTTCTGGCCGCCAAGAACCTGTCCGACGCCATC
CTGCTGAGCGACATCCTGAGAGTGAACACCGAGATCACCAAGGCCCCCCTGAGCGCCT
CTATGATCAAGAGATACGACGAGCACCACCAGGACCTGACCCTGCTGAAAGCTCTCGTG
CGGCAGCAGCTGCCTGAGAAGTACAAAGAGATTTTCTTCGACCAGAGCAAGAACGGCTA
CGCCGGCTACATTGACGGCGGAGCCAGCCAGGAAGAGTTCTACAAGTTCATCAAGCCCA
TCCTGGAAAAGATGGACGGCACCGAGGAACTGCTCGTGAAGCTGAACAGAGAGGACCT
GCTGCGGAAGCAGCGGACCTTCGACAACGGCAGCATCCCCCACCAGATCCACCTGGGA
GAGCTGCACGCCATTCTGCGGCGGCAGGAAGATTTTTACCCATTCCTGAAGGACAACCG
GGAAAAGATCGAGAAGATCCTGACCTTCCGCATCCCCTACTACGTGGGCCCTCTGGCCA
GGGGAAACAGCAGATTCGCCTGGATGACCAGAAAGAGCGAGGAAACCATCACCCCCTG
GAACTTCGAGGAAGTGGTGGACAAGGGCGCTTCCGCCCAGAGCTTCATCGAGCGGATG
ACCAACTTCGATAAGAACCTGCCCAACGAGAAGGTGCTGCCCAAGCACAGCCTGCTGTA
CGAGTACTTCACCGTGTATAACGAGCTGACCAAAGTGAAATACGTGACCGAGGGAATGA
GAAAGCCCGCCTTCCTGAGCGGCGAGCAGAAAAAGGCCATCGTGGACCTGCTGTTCAA
GACCAACCGGAAAGTGACCGTGAAGCAGCTGAAAGAGGACTACTTCAAGAAAATCGAGT
GCCTGTCCTACGAGACAGAGATCCTGACAGTGGAGTATGGCCTGCTGCCAATCGGCAAG
ATCGTGGAGAAGAGGATCGAGTGTACCGTGTACTCTGTGGATAACAATGGCAACATCTAT
ACACAGCCCGTGGCACAGTGGCACGATAGGGGAGAGCAGGAGGTGTTCGAGTATTGCC
TGGAGGACGGCAGCCTGATCAGGGCAACCAAGGACCACAAGTTCATGACAGTGGATGG

CCAGATGCTGCCCATCGACGAGATTTTCGAGCGGGAGCTGGACCTGATGAGAGTGGATA
ACCTGCCTAATAGCGGAGGCAGTAAAAGAACAGCAGACGGGAGTGAGTTTGAGCCCAAG
AAAAAGAGAAAGGTGTAAGATCTGATAATCAACCTCTGGATTACAAAATTTGTGAAAGATT
GACTGGTATTCTTAACTATGTTGCTCCTTTTACGCTATGTGGATACGCTGCTTTAATGCCTT
TGTATCATGCTATTGCTTCCCGTATGGCTTTCATTTTCTCCTCCTTGTATAAATCCTGGTTA
GTTCTTGCCACGGCGGAACTCATCGCCGCCTGCCTTGCCCGCTGCTGGACAGGGGCTC
GGCTGTTGGGCACTGACAATTCCGTGGTGCGACTGTGCCTTCTAGTTGCCAGCCATCTG
TTGTTTGCCCCTCCCCCGTGCCTTCCTTGACCCTGGAAGGTGCCACTCCCACTGTCCTT
TCCTAATAAAATGAGGAAATTGCATCGCATTGTCTGAGTAGGTGTCATTCTATTCTGGGGG
GTGGGGTGGGGCAGGACAGCAAGGGGGAGGATTGGGAAGACAATAGCAGGCATGCTG
GGGATGCGGTGGGCTCTATGGCTCGAGAAAAAAAGCACCGACTCGGTGCCACTTTTTCA
AGTTGATAACGGACTAGCCTTATTTAAACTTGCTATGCTGTTTCCAGCATAGCTCTTAAACT
GCCCCGGGTATCGGCTGCCGGTGTTTCGTCCTTTCCACAAGATATATAAAGCCAAGAAAT
CGAAATACTTTCAAGTTACGGTAAGCATATGATAGTCCATTTTAAAACATAATTTTAAAACTG
CAAACTACCCAAGAAATTATTACTTTCTACGTCACGTATTTTGTACTAATATCTTTGTGTTTA
CAGTCAAATTAATTCTAATTATCTCTCTAACAGCCTTGTATCGTATATGCAAATATGAAGGAA
TCATGGGAAATAGGCCCTCTTCCTGCCCGACCTTGCGGCCGCAGGAACCCCTAGTGATG
GAGTTGGCCACTCCCTCTCTGCGCGCTCGCTCGCTCACTGAGGCCGGGCGACCAAAGG
TCGCCCGACGCCCGGGCTTTGCCCGGGCGGCCTCAGTGAGCGAGCGAGCGCGCAG

Supplementary Sequence 4. Dual-AAV TadCBEd *PRNP* R37X F+E-sgRNA (C-terminus).

ITR-Cbh promoter-NLS-NpuC-SpCas9 (amino acids 573-1367)-UGI-NLS-WPRE-bovine growth hormone(bGH)-derived poly(A)-*PRNP* R37X F+E-sgRNA (reverse complement)-human U6 promoter (reverse complement)-ITR

CTGCGCGCTCGCTCGCTCACTGAGGCCGCCCGGGCAAAGCCCGGGCGTCGGGCGACC
TTTGGTCGCCCGGCCTCAGTGAGCGAGCGAGCGCGCAGAGAGGGAGTGGCCAACTCC
ATCACTAGGGGTTCCTGCGGCCTCTAGATCAGGGTACCCGTTACATAACTTACGGTAAAT
GGCCCGCCTGGCTGACCGCCCAACGACCCCCGCCCATTGACGTCAATAGTAACGCCAAT
AGGGACTTTCCATTGACGTCAATGGGTGGAGTATTTACGGTAAACTGCCCACTTGGCAGT
ACATCAAGTGTATCATATGCCAAGTACGCCCCCTATTGACGTCAATGACGGTAAATGGCCC
GCCTGGCATTGTGCCCAGTACATGACCTTATGGGACTTTCCTACTTGGCAGTACATCTAC
GTATTAGTCATCGCTATTACCATGGTCGAGGTGAGCCCCACGTTCTGCTTCACTCTCCCC
ATCTCCCCCCCCTCCCCACCCCCAATTTTGTATTTATTTATTTTTTAATTATTTTGTGCAGCG
ATGGGGGCGGGGGGGGGGGGGGCGCGCGCCAGGCGGGGCGGGGCGGGGCGAG
GGGCGGGGCGGGGCGAGGCGGAGAGGTGCGGCGGCAGCCAATCAGAGCGGCGCGCT
CCGAAAGTTTCCTTTTATGGCGAGGCGGCGGCGGCGGCGGCCCTATAAAAAGCGAAGC
GCGCGGCGGGCGGGAGTCGCTGCGACGCTGCCTTCGCCCCGTGCCCCGCTCCGCCG
CCGCCTCGCGCCGCCCGCCCCGGCTCTGACTGACCGCGTTACTCCCACAGGTGAGCG
GGCGGGACGGCCCTTCTCCTCCGGGCTGTAATTAGCTGAGCAAGAGGTAAGGGTTTAAG
GGATGGTTGGTTGGTGGGGTATTAATGTTTAATTACCTGGAGCACCTGCCTGAAATCACTT
TTTTTCAGGTTGGACCGGTGCCACCATGAAACGGACAGCCGACGGAAGCGAGTTCGAG
TCACCAAAGAAGAAGCGGAAAGTCATCAAGATTGCTACACGGAAATACCTGGGAAAGCA
GAACGTGTACGACATCGGCGTGGAGCGGGATCACAACTTCGCCCTGAAGAATGGCTTTA
TCGCCAGCAATTGCTTCGACTCCGTGGAAATCTCCGGCGTGGAAGATCGGTTCAACGCC
TCCCTGGGCACATACCACGATCTGCTGAAAATTATCAAGGACAAGGACTTCCTGGACAAT
GAGGAAAACGAGGACATTCTGGAAGATATCGTGCTGACCCTGACACTGTTTGAGGACAG
AGAGATGATCGAGGAACGGCTGAAAACCTATGCCCACCTGTTCGACGACAAAGTGATGA
AGCAGCTGAAGCGGCGGAGATACACCGGCTGGGGCAGGCTGAGCCGGAAGCTGATCA
ACGGCATCCGGGACAAGCAGTCCGGCAAGACAATCCTGGATTTCCTGAAGTCCGACGG
CTTCGCCAACAGAAACTTCATGCAGCTGATCCACGACGACAGCCTGACCTTTAAAGAGG
ACATCCAGAAAGCCCAGGTGTCCGGCCAGGGCGATAGCCTGCACGAGCACATTGCCAAT
CTGGCCGGCAGCCCCGCCATTAAGAAGGGCATCCTGCAGACAGTGAAGGTGGTGGACG

AGCTCGTGAAAGTGATGGGCCGGCACAAGCCCGAGAACATCGTGATCGAAATGGCCAG
AGAGAACCAGACCACCCAGAAGGGACAGAAGAACAGCCGCGAGAGAATGAAGCGGATC
GAAGAGGGCATCAAAGAGCTGGGCAGCCAGATCCTGAAAGAACACCCCGTGGAAAACA
CCCAGCTGCAGAACGAGAAGCTGTACCTGTACTACCTGCAGAATGGGCGGGATATGTAC
GTGGACCAGGAACTGGACATCAACCGGCTGTCCGACTACGATGTGGACCATATCGTGCC
TCAGAGCTTTCTGAAGGACGACTCCATCGACAACAAGGTGCTGACCAGAAGCGACAAGA
ACCGGGGCAAGAGCGACAACGTGCCCTCCGAAGAGGTCGTGAAGAAGATGAAGAACTA
CTGGCGGCAGCTGCTGAACGCCAAGCTGATTACCCAGAGAAAGTTCGACAATCTGACCA
AGGCCGAGAGAGGCGGCCTGAGCGAACTGGATAAGGCCGGCTTCATCAAGAGACAGCT
GGTGGAAACCCGGCAGATCACAAAGCACGTGGCACAGATCCTGGACTCCCGGATGAAC
ACTAAGTACGACGAGAATGACAAGCTGATCCGGGAAGTGAAAGTGATCACCCTGAAGTC
CAAGCTGGTGTCCGATTTCCGGAAGGATTTCCAGTTTTACAAAGTGCGCGAGATCAACAA
CTACCACCACGCCCACGACGCCTACCTGAACGCCGTCGTGGGAACCGCCCTGATCAAA
AAGTACCCTAAGCTGGAAAGCGAGTTCGTGTACGGCGACTACAAGGTGTACGACGTGCG
GAAGATGATCGCCAAGAGCGAGCAGGAAATCGGCAAGGCTACCGCCAAGTACTTCTTCT
ACAGCAACATCATGAACTTTTTCAAGACCGAGATTACCCTGGCCAACGGCGAGATCCGG
AAGCGGCCTCTGATCGAGACAAACGGCGAAACCGGGGAGATCGTGTGGGATAAGGGCC
GGGATTTTGCCACCGTGCGGAAAGTGCTGAGCATGCCCCAAGTGAATATCGTGAAAAAG
ACCGAGGTGCAGACAGGCGGCTTCAGCAAAGAGTCTATCCTGCCCAAGAGGAACAGCG
ATAAGCTGATCGCCAGAAAGAAGGACTGGGACCCTAAGAAGTACGGCGGCTTCGACAGC
CCCACCGTGGCCTATTCTGTGCTGGTGGTGGCCAAAGTGGAAAAGGGCAAGTCCAAGA
AACTGAAGAGTGTGAAAGAGCTGCTGGGGATCACCATCATGGAAAGAAGCAGCTTCGAG
AAGAATCCCATCGACTTTCTGGAAGCCAAGGGCTACAAAGAAGTGAAAAAGGACCTGAT
CATCAAGCTGCCTAAGTACTCCCTGTTCGAGCTGGAAAACGGCCGGAAGAGAATGCTGG
CCTCTGCCGGCGAACTGCAGAAGGGAAACGAACTGGCCCTGCCCTCCAAATATGTGAAC
TTCCTGTACCTGGCCAGCCACTATGAGAAGCTGAAGGGCTCCCCCGAGGATAATGAGCA
GAAACAGCTGTTTGTGGAACAGCACAAGCACTACCTGGACGAGATCATCGAGCAGATCA
GCGAGTTCTCCAAGAGAGTGATCCTGGCCGACGCTAATCTGGACAAAGTGCTGTCCGCC
TACAACAAGCACCGGGATAAGCCCATCAGAGAGCAGGCCGAGAATATCATCCACCTGTTT
ACCCTGACCAATCTGGGAGCCCCTGCCGCCTTCAAGTACTTTGACACCACCATCGACCG
GAAGAGGTACACCAGCACCAAAGAGGTGCTGGACGCCACCCTGATCCACCAGAGCATC
ACCGGCCTGTACGAGACACGGATCGACCTGTCTCAGCTGGGAGGTGACAGCGGCGGG
AGCGGCGGGAGCGGGGGGAGCACTAATCTGAGCGACATCATTGAGAAGGAGACTGGGA

AACAGCTGGTCATTCAGGAGTCCATCCTGATGCTGCCTGAGGAGGTGGAGGAAGTGATC
GGCAACAAGCCAGAGTCTGACATCCTGGTGCACACCGCCTACGACGAGTCCACAGATGA
GAATGTGATGCTGCTGACCTCTGACGCCCCCGAGTATAAGCCTTGGGCCCTGGTCATCC
AGGATTCTAACGGCGAGAATAAGATCAAGATGCTGAGCGGAGGATCCAAAAGAACCGCC
GACGGCAGCGAATTCGAGCCCAAGAAGAAGAGGAAAGTCTAAGATCTGATAATCAACCT
CTGGATTACAAAATTTGTGAAAGATTGACTGGTATTCTTAACTATGTTGCTCCTTTTACGCT
ATGTGGATACGCTGCTTTAATGCCTTTGTATCATGCTATTGCTTCCCGTATGGCTTTCATTT
TCTCCTCCTTGTATAAATCCTGGTTAGTTCTTGCCACGGCGGAACTCATCGCCGCCTGCC
TTGCCCGCTGCTGGACAGGGGCTCGGCTGTTGGGCACTGACAATTCCGTGGTGCGACT
GTGCCTTCTAGTTGCCAGCCATCTGTTGTTTGCCCCTCCCCCGTGCCTTCCTTGACCCT
GGAAGGTGCCACTCCCACTGTCCTTTCCTAATAAAATGAGGAAATTGCATCGCATTGTCT
GAGTAGGTGTCATTCTATTCTGGGGGGTGGGGTGGGGCAGGACAGCAAGGGGGAGGAT
TGGGAAGACAATAGCAGGCATGCTGGGGATGCGGTGGGCTCTATGGCTCGAGAAAAAAA
GCACCGACTCGGTGCCACTTTTTCAAGTTGATAACGGACTAGCCTTATTTAAACTTGCTAT
GCTGTTTCCAGCATAGCTCTTAAACTGCCCCGGGTATCGGCTGCCGGTGTTTCGTCCTTT
CCACAAGATATATAAAGCCAAGAAATCGAAATACTTTCAAGTTACGGTAAGCATATGATAGT
CCATTTTAAAACATAATTTTAAAACTGCAAACTACCCAAGAAATTATTACTTTCTACGTCACG
TATTTTGTACTAATATCTTTGTGTTTACAGTCAAATTAATTCTAATTATCTCTCTAACAGCCTT
GTATCGTATATGCAAATATGAAGGAATCATGGGAAATAGGCCCTCTTCCTGCCCGACCTTG
CGGCCGCAGGAACCCCTAGTGATGGAGTTGGCCACTCCCTCTCTGCGCGCTCGCTCGC
TCACTGAGGCCGGGCGACCAAAGGTCGCCCGACGCCCGGGCTTTGCCCGGGCGGCCT
CAGTGAGCGAGCGAGCGCGCAG

Supplementary Sequence 5. Dual-AAV TadCBEd *PRNP* R37X F+E-sgRNA (N-terminus) with hSYN promoter, 3xmiR-183 and 3xmiR-122 target sites.

ITR-hSYN promoter-NLS-TadCBEd deaminase domain-linker-SpCas9 (amino acids 1-572)-NpuN-NLS-3x miR-183+ 3x miR-122 target sites-WPRE-bovine growth hormone(bGH)-derived poly(A)-*PRNP* R37X F+E-sgRNA (reverse complement)-human U6 promoter (reverse complement)-ITR

CTGCGCGCTCGCTCGCTCACTGAGGCCGCCCGGGCAAAGCCCGGGCGTCGGGCGACC
TTTGGTCGCCCGGCCTCAGTGAGCGAGCGAGCGCGCAGAGAGGGAGTGGCCAACTCC
ATCACTAGGGGTTCCTGCGGCCTCTAGATCAGGGTACCAGTGCAAGTGGGTTTTAGGAC
CAGGATGAGGCGGGGTGGGGGTGCCTACCTGACGACCGACCCCGACCCACTGGACAA
GCACCCAACCCCCATTCCCCAAATTGCGCATCCCCTATCAGAGAGGGGGAGGGGAAACA
GGATGCGGCGAGGCGCGTGCGCACTGCCAGCTTCAGCACCGCGGACAGTGCCTTCGC
CCCCGCCTGGCGGCGCGCGCCACCGCCGCCTCAGCACTGAAGGCGCGCTGACGTCAC
TCGCCGGTCCCCCGCAAACTCCCCTTCCCGGCCACCTTGGTCGCGTCCGCGCCGCCG
CCGGCCCAGCCGGACCGCACCACGCGAGGCGCGAGATAGGGGGGCACGGGCGCGAC
CATCTGCGCTGCGGCGCCGGCGACTCAGCGCTGCCTCAGTCTGCGGTGGGCAGCGGA
GGAGTCGTGTCGTGCCTGAGAGCGCAGGACCGGTGCCACCATGAAACGGACAGCCGA
CGGAAGCGAGTTCGAGTCACCAAAGAAGAAGCGGAAAGTCAGTTCTGAGGTGGAGTTTT
CCCACGAGTACTGGATGAGACATGCCCTGACCCTGGCCAAGAGGGCACGGGATGAGAG
GAAGGCGCCTGTGGGAGCCGTGCTGGTGCTGAACAATAGAGTGATCGGCGAGGGCTGG
AACAGAGCCATCGGCCTGCACGACCCAACAGCCCATGCCGAAATTATAGCCCTGAGACA
GGGCGGCCTGGTCATGCAGAACTACAGACTGATTGACGCCACCCTGTACGTGACATTCG
AGCCTTGCGTGATGTGCGCCGGCGCCATGATCAACTCTAGGATCGGCCGCGTGGTGTTT
GGCGTGAGGAACTCAAAAAGAGGCGCCGCAGGCTCCCTGATGAACGTGCTGAACTACC
CCGGAATGAATCACCGCGTCGAAATTACCGAGGGAATCCTGGCAGATGAATGTGCCGCC
CTGCTGTGCGATTTCTATCGGATGCCTAGACAGGTGTTCAATGCTCAGAAGAAGGCCCAG
AGCTCCATCAACTCTGGAGGATCTAGCGGAGGATCCTCTGGCAGCGAGACACCAGGAAC
AAGCGAGTCAGCAACACCAGAGAGCAGTGGCGGCAGCAGCGGCGGCAGCGACAAGAA
GTACAGCATCGGCCTGGCCATCGGCACCAACTCTGTGGGCTGGGCCGTGATCACCGAC
GAGTACAAGGTGCCCAGCAAGAAATTCAAGGTGCTGGGCAACACCGACCGGCACAGCA
TCAAGAAGAACCTGATCGGAGCCCTGCTGTTCGACAGCGGCGAAACAGCCGAGGCCAC
CCGGCTGAAGAGAACCGCCAGAAGAAGATACACCAGACGGAAGAACCGGATCTGCTATC
TGCAAGAGATCTTCAGCAACGAGATGGCCAAGGTGGACGACAGCTTCTTCCACAGACTG

GAAGAGTCCTTCCTGGTGGAAGAGGATAAGAAGCACGAGCGGCACCCCATCTTCGGCA
ACATCGTGGACGAGGTGGCCTACCACGAGAAGTACCCCACCATCTACCACCTGAGAAAG
AAACTGGTGGACAGCACCGACAAGGCCGACCTGCGGCTGATCTATCTGGCCCTGGCCC
ACATGATCAAGTTCCGGGGCCACTTCCTGATCGAGGGCGACCTGAACCCCGACAACAGC
GACGTGGACAAGCTGTTCATCCAGCTGGTGCAGACCTACAACCAGCTGTTCGAGGAAAA
CCCCATCAACGCCAGCGGCGTGGACGCCAAGGCCATCCTGTCTGCCAGACTGAGCAAG
AGCAGACGGCTGGAAAATCTGATCGCCCAGCTGCCCGGCGAGAAGAAGAATGGCCTGT
TCGGAAACCTGATTGCCCTGAGCCTGGGCCTGACCCCCAACTTCAAGAGCAACTTCGAC
CTGGCCGAGGATGCCAAACTGCAGCTGAGCAAGGACACCTACGACGACGACCTGGACA
ACCTGCTGGCCCAGATCGGCGACCAGTACGCCGACCTGTTTCTGGCCGCCAAGAACCT
GTCCGACGCCATCCTGCTGAGCGACATCCTGAGAGTGAACACCGAGATCACCAAGGCC
CCCCTGAGCGCCTCTATGATCAAGAGATACGACGAGCACCACCAGGACCTGACCCTGCT
GAAAGCTCTCGTGCGGCAGCAGCTGCCTGAGAAGTACAAAGAGATTTTCTTCGACCAGA
GCAAGAACGGCTACGCCGGCTACATTGACGGCGGAGCCAGCCAGGAAGAGTTCTACAA
GTTCATCAAGCCCATCCTGGAAAAGATGGACGGCACCGAGGAACTGCTCGTGAAGCTGA
ACAGAGAGGACCTGCTGCGGAAGCAGCGGACCTTCGACAACGGCAGCATCCCCCACCA
GATCCACCTGGGAGAGCTGCACGCCATTCTGCGGCGGCAGGAAGATTTTTACCCATTCC
TGAAGGACAACCGGGAAAAGATCGAGAAGATCCTGACCTTCCGCATCCCCTACTACGTG
GGCCCTCTGGCCAGGGGAAACAGCAGATTCGCCTGGATGACCAGAAAGAGCGAGGAAA
CCATCACCCCCTGGAACTTCGAGGAAGTGGTGGACAAGGGCGCTTCCGCCCAGAGCTT
CATCGAGCGGATGACCAACTTCGATAAGAACCTGCCCAACGAGAAGGTGCTGCCCAAGC
ACAGCCTGCTGTACGAGTACTTCACCGTGTATAACGAGCTGACCAAAGTGAAATACGTGA
CCGAGGGAATGAGAAAGCCCGCCTTCCTGAGCGGCGAGCAGAAAAAGGCCATCGTGGA
CCTGCTGTTCAAGACCAACCGGAAAGTGACCGTGAAGCAGCTGAAAGAGGACTACTTCA
AGAAAATCGAGTGCCTGTCCTACGAGACAGAGATCCTGACAGTGGAGTATGGCCTGCTG
CCAATCGGCAAGATCGTGGAGAAGAGGATCGAGTGTACCGTGTACTCTGTGGATAACAAT
GGCAACATCTATACACAGCCCGTGGCACAGTGGCACGATAGGGGAGAGCAGGAGGTGT
TCGAGTATTGCCTGGAGGACGGCAGCCTGATCAGGGCAACCAAGGACCACAAGTTCATG
ACAGTGGATGGCCAGATGCTGCCCATCGACGAGATTTTCGAGCGGGAGCTGGACCTGAT
GAGAGTGGATAACCTGCCTAATAGCGGAGGCAGTAAAAGAACAGCAGACGGGAGTGAGT
TTGAGCCCAAGAAAAAGAGAAAGGTGTAAGGTACCAGTGAATTCTACCAGTGCCATAGGA
TAGTGAATTCTACCAGTGCCATACACGTGAGTGAATTCTACCAGTGCCATAGCATGCCAAA
CACCATTGTCACACTCCATACTATCAAACACCATTGTCACACTCCAGATCAACAAACACCA

TTGTCACACTCCAGATCTGATAATCAACCTCTGGATTACAAAATTTGTGAAAGATTGACTG
GTATTCTTAACTATGTTGCTCCTTTTACGCTATGTGGATACGCTGCTTTAATGCCTTTGTATC
ATGCTATTGCTTCCCGTATGGCTTTCATTTTCTCCTCCTTGTATAAATCCTGGTTAGTTCTT
GCCACGGCGGAACTCATCGCCGCCTGCCTTGCCCGCTGCTGGACAGGGGCTCGGCTG
TTGGGCACTGACAATTCCGTGGTGCGACTGTGCCTTCTAGTTGCCAGCCATCTGTTGTTT
GCCCCTCCCCCGTGCCTTCCTTGACCCTGGAAGGTGCCACTCCCACTGTCCTTTCCTAA
TAAAATGAGGAAATTGCATCGCATTGTCTGAGTAGGTGTCATTCTATTCTGGGGGGTGGG
GTGGGGCAGGACAGCAAGGGGGAGGATTGGGAAGACAATAGCAGGCATGCTGGGGAT
GCGGTGGGCTCTATGGCTCGAGAAAAAAAGCACCGACTCGGTGCCACTTTTTCAAGTTG
ATAACGGACTAGCCTTATTTAAACTTGCTATGCTGTTTCCAGCATAGCTCTTAAACTGCCCC
GGGTATCGGCTGCCGGTGTTTCGTCCTTTCCACAAGATATATAAAGCCAAGAAATCGAAAT
ACTTTCAAGTTACGGTAAGCATATGATAGTCCATTTTAAAACATAATTTTAAAACTGCAAACT
ACCCAAGAAATTATTACTTTCTACGTCACGTATTTTGTACTAATATCTTTGTGTTTACAGTCA
AATTAATTCTAATTATCTCTCTAACAGCCTTGTATCGTATATGCAAATATGAAGGAATCATGG
GAAATAGGCCCTCTTCCTGCCCGACCTTGCGGCCGCAGGAACCCCTAGTGATGGAGTTG
GCCACTCCCTCTCTGCGCGCTCGCTCGCTCACTGAGGCCGGGCGACCAAAGGTCGCCC
GACGCCCGGGCTTTGCCCGGGCGGCCTCAGTGAGCGAGCGAGCGCGCAG

Supplementary Sequence 6. Dual-AAV TadCBEd *PRNP* R37X F+E-sgRNA (C-terminus) with hSYN promoter, 3xmiR-183 and 3xmiR-122 target sites.

ITR-hSYN promoter-NLS-NpuC-SpCas9 (amino acids 573-1367)-UGI-NLS-3x miR-183 + 3x miR-122 target sites-WPRE-bovine growth hormone(bGH)-derived poly(A)-*PRNP* R37X F+E-sgRNA (reverse complement)-human U6 promoter (reverse complement)-ITR

CTGCGCGCTCGCTCGCTCACTGAGGCCGCCCGGGCAAAGCCCGGGCGTCGGGCGACC
TTTGGTCGCCCGGCCTCAGTGAGCGAGCGAGCGCGCAGAGAGGGAGTGGCCAACTCC
ATCACTAGGGGTTCCTGCGGCCTCTAGATCAGGGTACCAGTGCAAGTGGGTTTTAGGAC
CAGGATGAGGCGGGGTGGGGGTGCCTACCTGACGACCGACCCCGACCCACTGGACAA
GCACCCAACCCCCATTCCCCAAATTGCGCATCCCCTATCAGAGAGGGGGAGGGGAAACA
GGATGCGGCGAGGCGCGTGCGCACTGCCAGCTTCAGCACCGCGGACAGTGCCTTCGC
CCCCGCCTGGCGGCGCGCGCCACCGCCGCCTCAGCACTGAAGGCGCGCTGACGTCAC
TCGCCGGTCCCCCGCAAACTCCCCTTCCCGGCCACCTTGGTCGCGTCCGCGCCGCCG
CCGGCCCAGCCGGACCGCACCACGCGAGGCGCGAGATAGGGGGGCACGGGCGCGAC
CATCTGCGCTGCGGCGCCGGCGACTCAGCGCTGCCTCAGTCTGCGGTGGGCAGCGGA
GGAGTCGTGTCGTGCCTGAGAGCGCAGGACCGGTGCCACCATGAAACGGACAGCCGA
CGGAAGCGAGTTCGAGTCACCAAAGAAGAAGCGGAAAGTCATCAAGATTGCTACACGGA
AATACCTGGGAAAGCAGAACGTGTACGACATCGGCGTGGAGCGGGATCACAACTTCGCC
CTGAAGAATGGCTTTATCGCCAGCAATTGCTTCGACTCCGTGGAAATCTCCGGCGTGGA
AGATCGGTTCAACGCCTCCCTGGGCACATACCACGATCTGCTGAAAATTATCAAGGACAA
GGACTTCCTGGACAATGAGGAAAACGAGGACATTCTGGAAGATATCGTGCTGACCCTGA
CACTGTTTGAGGACAGAGAGATGATCGAGGAACGGCTGAAAACCTATGCCCACCTGTTC
GACGACAAAGTGATGAAGCAGCTGAAGCGGCGGAGATACACCGGCTGGGGCAGGCTGA
GCCGGAAGCTGATCAACGGCATCCGGGACAAGCAGTCCGGCAAGACAATCCTGGATTTC
CTGAAGTCCGACGGCTTCGCCAACAGAAACTTCATGCAGCTGATCCACGACGACAGCCT
GACCTTTAAAGAGGACATCCAGAAAGCCCAGGTGTCCGGCCAGGGCGATAGCCTGCAC
GAGCACATTGCCAATCTGGCCGGCAGCCCCGCCATTAAGAAGGGCATCCTGCAGACAGT
GAAGGTGGTGGACGAGCTCGTGAAAGTGATGGGCCGGCACAAGCCCGAGAACATCGTG
ATCGAAATGGCCAGAGAGAACCAGACCACCCAGAAGGGACAGAAGAACAGCCGCGAGA
GAATGAAGCGGATCGAAGAGGGCATCAAAGAGCTGGGCAGCCAGATCCTGAAAGAACA
CCCCGTGGAAAACACCCAGCTGCAGAACGAGAAGCTGTACCTGTACTACCTGCAGAATG
GGCGGGATATGTACGTGGACCAGGAACTGGACATCAACCGGCTGTCCGACTACGATGTG

GACCATATCGTGCCTCAGAGCTTTCTGAAGGACGACTCCATCGACAACAAGGTGCTGAC
CAGAAGCGACAAGAACCGGGGCAAGAGCGACAACGTGCCCTCCGAAGAGGTCGTGAA
GAAGATGAAGAACTACTGGCGGCAGCTGCTGAACGCCAAGCTGATTACCCAGAGAAAGT
TCGACAATCTGACCAAGGCCGAGAGAGGCGGCCTGAGCGAACTGGATAAGGCCGGCTT
CATCAAGAGACAGCTGGTGGAAACCCGGCAGATCACAAAGCACGTGGCACAGATCCTG
GACTCCCGGATGAACACTAAGTACGACGAGAATGACAAGCTGATCCGGGAAGTGAAAGT
GATCACCCTGAAGTCCAAGCTGGTGTCCGATTTCCGGAAGGATTTCCAGTTTTACAAAGT
GCGCGAGATCAACAACTACCACCACGCCCACGACGCCTACCTGAACGCCGTCGTGGGA
ACCGCCCTGATCAAAAAGTACCCTAAGCTGGAAAGCGAGTTCGTGTACGGCGACTACAA
GGTGTACGACGTGCGGAAGATGATCGCCAAGAGCGAGCAGGAAATCGGCAAGGCTACC
GCCAAGTACTTCTTCTACAGCAACATCATGAACTTTTTCAAGACCGAGATTACCCTGGCCA
ACGGCGAGATCCGGAAGCGGCCTCTGATCGAGACAAACGGCGAAACCGGGGAGATCGT
GTGGGATAAGGGCCGGGATTTTGCCACCGTGCGGAAAGTGCTGAGCATGCCCCAAGTG
AATATCGTGAAAAAGACCGAGGTGCAGACAGGCGGCTTCAGCAAAGAGTCTATCCTGCC
CAAGAGGAACAGCGATAAGCTGATCGCCAGAAAGAAGGACTGGGACCCTAAGAAGTACG
GCGGCTTCGACAGCCCCACCGTGGCCTATTCTGTGCTGGTGGTGGCCAAAGTGGAAAA
GGGCAAGTCCAAGAAACTGAAGAGTGTGAAAGAGCTGCTGGGGATCACCATCATGGAAA
GAAGCAGCTTCGAGAAGAATCCCATCGACTTTCTGGAAGCCAAGGGCTACAAAGAAGTG
AAAAAGGACCTGATCATCAAGCTGCCTAAGTACTCCCTGTTCGAGCTGGAAAACGGCCG
GAAGAGAATGCTGGCCTCTGCCGGCGAACTGCAGAAGGGAAACGAACTGGCCCTGCCC
TCCAAATATGTGAACTTCCTGTACCTGGCCAGCCACTATGAGAAGCTGAAGGGCTCCCCC
GAGGATAATGAGCAGAAACAGCTGTTTGTGGAACAGCACAAGCACTACCTGGACGAGAT
CATCGAGCAGATCAGCGAGTTCTCCAAGAGAGTGATCCTGGCCGACGCTAATCTGGACA
AAGTGCTGTCCGCCTACAACAAGCACCGGGATAAGCCCATCAGAGAGCAGGCCGAGAAT
ATCATCCACCTGTTTACCCTGACCAATCTGGGAGCCCCTGCCGCCTTCAAGTACTTTGAC
ACCACCATCGACCGGAAGAGGTACACCAGCACCAAAGAGGTGCTGGACGCCACCCTGA
TCCACCAGAGCATCACCGGCCTGTACGAGACACGGATCGACCTGTCTCAGCTGGGAGG
TGACAGCGGCGGGAGCGGCGGGAGCGGGGGGAGCACTAATCTGAGCGACATCATTGA
GAAGGAGACTGGGAAACAGCTGGTCATTCAGGAGTCCATCCTGATGCTGCCTGAGGAG
GTGGAGGAAGTGATCGGCAACAAGCCAGAGTCTGACATCCTGGTGCACACCGCCTACG
ACGAGTCCACAGATGAGAATGTGATGCTGCTGACCTCTGACGCCCCCGAGTATAAGCCT
TGGGCCCTGGTCATCCAGGATTCTAACGGCGAGAATAAGATCAAGATGCTGAGCGGAGG
ATCCAAAAGAACCGCCGACGGCAGCGAATTCGAGCCCAAGAAGAAGAGGAAAGTCTAAG

GTACCAGTGAATTCTACCAGTGCCATAGGATAGTGAATTCTACCAGTGCCATACACGTGAG
TGAATTCTACCAGTGCCATAGCATGCCAAACACCATTGTCACACTCCATACTATCAAACAC
CATTGTCACACTCCAGATCAACAAACACCATTGTCACACTCCAGATCTGATAATCAACCTC
TGGATTACAAAATTTGTGAAAGATTGACTGGTATTCTTAACTATGTTGCTCCTTTTACGCTAT
GTGGATACGCTGCTTTAATGCCTTTGTATCATGCTATTGCTTCCCGTATGGCTTTCATTTTC
TCCTCCTTGTATAAATCCTGGTTAGTTCTTGCCACGGCGGAACTCATCGCCGCCTGCCTT
GCCCGCTGCTGGACAGGGGCTCGGCTGTTGGGCACTGACAATTCCGTGGTGCGACTGT
GCCTTCTAGTTGCCAGCCATCTGTTGTTTGCCCCTCCCCCGTGCCTTCCTTGACCCTGG
AAGGTGCCACTCCCACTGTCCTTTCCTAATAAAATGAGGAAATTGCATCGCATTGTCTGAG
TAGGTGTCATTCTATTCTGGGGGGTGGGGTGGGGCAGGACAGCAAGGGGGAGGATTGG
GAAGACAATAGCAGGCATGCTGGGGATGCGGTGGGCTCTATGGCTCGAGAAAAAAAGCA
CCGACTCGGTGCCACTTTTTCAAGTTGATAACGGACTAGCCTTATTTAAACTTGCTATGCT
GTTTCCAGCATAGCTCTTAAACTGCCCCGGGTATCGGCTGCCGGTGTTTCGTCCTTTCCA
CAAGATATATAAAGCCAAGAAATCGAAATACTTTCAAGTTACGGTAAGCATATGATAGTCCA
TTTTAAAACATAATTTTAAAACTGCAAACTACCCAAGAAATTATTACTTTCTACGTCACGTAT
TTTGTACTAATATCTTTGTGTTTACAGTCAAATTAATTCTAATTATCTCTCTAACAGCCTTGTA
TCGTATATGCAAATATGAAGGAATCATGGGAAATAGGCCCTCTTCCTGCCCGACCTTGCG
GCCGCAGGAACCCCTAGTGATGGAGTTGGCCACTCCCTCTCTGCGCGCTCGCTCGCTC
ACTGAGGCCGGGCGACCAAAGGTCGCCCGACGCCCGGGCTTTGCCCGGGCGGCCTCA
GTGAGCGAGCGAGCGCGCAG

Supplementary Sequence 7. Single-AAV SauriCas9-TadCBEd with *PRNP* R37X F-sgRNA.

ITR-EFS promoter-NLS-TadCBEd deaminase domain-linker 1-SauriCas9-linker 2-UGI-NLS-synthetic poly(A)-*PRNP* R37X F-sgRNA (reverse complement)-human U6 promoter (reverse complement)-ITR

CTGCGCGCTCGCTCGCTCACTGAGGCCGCCCGGGCAAAGCCCGGGCGTCGGGCGACC
TTTGGTCGCCCGGCCTCAGTGAGCGAGCGAGCGCGCAGAGAGGGAGTGGCCAACTCC
ATCACTAGGGGTTCCTGCGGCCTCTAGATAGGTCTTGAAAGGAGTGGGAATTGGCTCCG
GTGCCCGTCAGTGGGCAGAGCGCACATCGCCCACAGTCCCCGAGAAGTTGGGGGGAG
GGGTCGGCAATTGATCCGGTGCCTAGAGAAGGTGGCGCGGGGTAAACTGGGAAAGTGA
TGTCGTGTACTGGCTCCGCCTTTTTCCCGAGGGTGGGGGAGAACCGTATATAAGTGCAG
TAGTCGCCGTGAACGTTCTTTTTCGCAACGGGTTTGCCGCCAGAACACAGGACCGGTGC
CACCATGCCTGCCGCTAAGAGAGTGAAGCTGGACTCTGAGGTGGAGTTTTCCCACGAGT
ACTGGATGAGACATGCCCTGACCCTGGCCAAGAGGGCACGGGATGAGAGGAAGGCGCC
TGTGGGAGCCGTGCTGGTGCTGAACAATAGAGTGATCGGCGAGGGCTGGAACAGAGCC
ATCGGCCTGCACGACCCAACAGCCCATGCCGAAATTATAGCCCTGAGACAGGGCGGCCT
GGTCATGCAGAACTACAGACTGATTGACGCCACCCTGTACGTGACATTCGAGCCTTGCG
TGATGTGCGCCGGCGCCATGATCAACTCTAGGATCGGCCGCGTGGTGTTTGGCGTGAG
GAACTCAAAAAGAGGCGCCGCAGGCTCCCTGATGAACGTGCTGAACTACCCCGGAATGA
ATCACCGCGTCGAAATTACCGAGGGAATCCTGGCAGATGAATGTGCCGCCCTGCTGTGC
GATTTCTATCGGATGCCTAGACAGGTGTTCAATGCTCAGAAGAAGGCCCAGAGCTCCATC
AACTCTGGCTCTGAGACACCTGGCACAAGCGAGAGCGCAACACCTGAAAGCCAGGAGA
ACCAGCAGAAGCAAAATTACATCCTGGGCCTGGCCATCGGCATCACCAGCGTGGGCTAT
GGCCTGATCGACAGCAAGACCAGAGAAGTGATTGACGCCGGCGTGCGGCTATTCCCAG
AGGCCGACTCTGAAAACAACAGCAATAGAAGATCTAAGCGGGGCGCCCGGAGACTGAAA
AGACGAAGAATCCACAGACTGAACAGAGTGAAAGACCTCCTGGCTGACTACCAGATGAT
CGACTTAAACAACGTGCCCAAGTCTACCGACCCCTACACCATCCGGGTGAAGGGACTGC
GGGAACCTCTGACCAAGGAAGAGTTTGCCATCGCTCTGCTGCATATCGCCAAGAGAAGA
GGCCTGCACAACATCTCCGTGAGCATGGGCGATGAGGAACAGGACAACGAGCTGTCCA
CCAAGCAGCAGCTGCAGAAGAACGCTCAGCAGCTGCAGGACAAATACGTGTGCGAGCT
GCAACTGGAAAGACTGACCAACATCAACAAGGTCAGAGGCGAGAAGAACCGGTTCAAG
ACCGAAGATTTCGTGAAGGAAGTGAAGCAGCTGTGCGAGACCCAGCGGCAGTACCACA

ACATCGACGATCAGTTCATCCAGCAGTACATCGACCTGGTGAGCACCCGGAGAGAATACT
TCGAGGGCCCTGGCAACGGCTCTCCATATGGCTGGGATGGAGATCTGCTGAAGTGGTAT
GAGAAGCTGATGGGCAGATGCACCTACTTCCCTGAGGAGCTGAGAAGCGTGAAGTACG
CCTACAGCGCCGATCTGTTTAACGCCCTGAACGATCTGAACAACCTGGTTGTGACCCGC
GACGACAACCCTAAGCTGGAATACTACGAGAAATACCACATTATCGAGAACGTGTTCAAG
CAGAAGAAAAATCCAACTCTGAAGCAAATCGCCAAAGAGATCGGCGTGCAGGATTACGA
CATCAGAGGATACAGAATTACCAAGTCCGGTAAGCCTCAGTTCACCAGCTTCAAACTCTA
CCACGACCTGAAAAACATCTTTGAACAGGCCAAATACCTGGAAGATGTGGAGATGCTGG
ATGAGATAGCTAAAATCCTGACAATCTACCAAGACGAGATCAGCATCAAGAAAGCCCTGG
ACCAGCTGCCTGAGCTGCTGACCGAGAGCGAAAAAAGCCAGATCGCTCAGCTGACCGG
CTACACCGGTACACATAGACTGTCTCTGAAGTGCATCCACATCGTGATCGACGAGCTGTG
GGAGAGCCCCGAAAACCAGATGGAAATCTTCACCAGACTGAACCTCAAGCCAAAGAAGG
TGGAAATGAGCGAGATCGACAGCATCCCTACCACACTGGTGGATGAGTTCATCCTGAGC
CCTGTGGTGAAGCGGGCCTTCATCCAGTCCATCAAGGTGATCAACGCTGTGATCAACAG
ATTCGGCCTGCCCGAGGACATCATTATCGAGCTGGCCAGAGAGAAGAACAGCAAGGACA
GAAGGAAGTTCATCAACAAGCTGCAGAAACAGAATGAGGCCACCCGGAAAAAAATCGAG
CAGCTGCTGGCCAAGTACGGCAATACCAATGCCAAGTACATGATCGAAAAGATCAAGCTG
CATGACATGCAGGAGGGCAAGTGTCTGTACAGCCTGGAAGCTATCCCCCTGGAAGACCT
GCTGTCTAATCCTACACACTACGAGGTGGACCACATCATCCCTAGAAGCGTGTCCTTCGA
CAACAGCCTGAACAACAAGGTTCTGGTGAAGCAAAGCGAGAACAGCAAGAAGGGCAATA
GGACCCCTTACCAGTACCTGAGCAGCAACGAGTCCAAGATCTCTTACAACCAGTTCAAG
CAGCACATTCTGAACCTGTCTAAGGCCAAAGATAGAATCAGCAAGAAGAAACGAGATATG
CTGCTGGAAGAACGGGACATCAACAAATTCGAGGTGCAGAAGGAATTCATCAACAGAAA
CCTTGTGGACACCCGGTACGCCACTCGGGAGCTGAGCAACCTGCTGAAGACCTACTTCA
GCACACACGACTACGCCGTGAAAGTGAAGACCATCAACGGCGGCTTCACAAACCACCTG
AGGAAGGTGTGGGACTTCAAGAAGCACCGGAACCACGGCTACAAGCACCACGCCGAGG
ATGCCCTGGTCATCGCCAACGCCGACTTTCTGTTCAAAACCCACAAGGCCCTGAGACGG
ACAGACAAGATCCTGGAACAGCCTGGACTGGAAGTCAACGACACCACCGTGAAGGTGG
ACACAGAGGAGAAGTACCAGGAGTTATTCGAGACACCGAAACAAGTGAAGAACATCAAG
CAGTTTAGAGATTTCAAGTATTCTCACAGAGTTGACAAGAAGCCCAACCGGCAGCTGATC
AATGATACCCTGTACAGTACCAGAGAGATCGATGGCGAAACCTACGTGGTCCAAACACTG
AAAGACCTGTACGCCAAGGACAATGAAAAGGTGAAAAAGCTCTTTACAGAACGGCCTCA
AAAGATACTGATGTACCAGCACGATCCTAAGACCTTTGAGAAACTGATGACCATTCTGAAT

CAGTACGCTGAGGCAAAGAATCCTCTGGCCGCTTATTACGAGGATAAGGGCGAATACGTG
ACCAAGTACGCCAAGAAGGGCAACGGCCCTGCCATCCACAAGATCAAATACATCGACAA
GAAACTGGGCAGCTACCTGGACGTGAGTAACAAATATCCTGAGACACAGAACAAGCTGG
TGAAACTGTCTCTGAAGAGCTTTAGATTCGACATCTACAAATGTGAACAGGGCTACAAGAT
GGTGTCCATTGGCTACCTCGACGTACTGAAGAAGGACAACTACTACTACATCCCCAAAGA
TAAGTACGAGGCCGAGAAGCAGAAAAAGAAGATCAAGGAAAGCGACCTCTTCGTGGGCA
GCTTCTACTACAACGACCTGATCATGTACGAGGACGAACTCTTCCGGGTGATCGGAGTGA
ACTCCGATATCAACAACCTGGTTGAGCTGAATATGGTCGACATCACCTACAAGGATTTCTG
CGAGGTGAACAACGTGACAGGCGAGAAGAGAATCAAGAAAACCATCGGAAAGAGAGTG
GTGCTGATCGAGAAGTATACGACCGACATCCTGGGAAATCTGTATAAAACGCCCCTGCCT
AAGAAGCCCCAGCTCATTTTCAAGAGAGGCGAGCTGAGCGGCGGGAGCGGCGGGAGC
GGGGGGAGCACTAATCTGAGCGACATCATTGAGAAGGAGACTGGGAAACAGCTGGTCAT
TCAGGAGTCCATCCTGATGCTGCCTGAGGAGGTGGAGGAAGTGATCGGCAACAAGCCA
GAGTCTGACATCCTGGTGCACACCGCCTACGACGAGTCCACAGATGAGAATGTGATGCT
GCTGACCTCTGACGCCCCCGAGTATAAGCCTTGGGCCCTGGTCATCCAGGATTCTAACG
GCGAGAATAAGATCAAGATGCTGAGCGGAGGATCCAAAAGAACCGCCGACGGCAGCGA
ATTCGAGCCCAAGAAGAAGAGGAAAGTCTAACTCGAGAATAAAATATCTTTATTTTCATTAC
ATCTGTGTGTTGGTTTTTTGTGTGAAGCTTAAAAAAATCTCGCCAACAAGTTGACGAGATA
AACACGGCATTTTGCCTTGTTATAGTAGATTCTGTAATTTTCATTACAGAGTACTATAACGC
CCCGGGTATCGGCTGCCCCGGTGTTTCGTCCTTTCCACAAGATATATAAAGCCAAGAAAT
CGAAATACTTTCAAGTTACGGTAAGCATATGATAGTCCATTTTAAAACATAATTTTAAAACTG
CAAACTACCCAAGAAATTATTACTTTCTACGTCACGTATTTTGTACTAATATCTTTGTGTTTA
CAGTCAAATTAATTCTAATTATCTCTCTAACAGCCTTGTATCGTATATGCAAATATGAAGGAA
TCATGGGAAATAGGCCCTCTTCCTGCCCGACCTTGCGGCCGCAGGAACCCCTAGTGATG
GAGTTGGCCACTCCCTCTCTGCGCGCTCGCTCGCTCACTGAGGCCGGGCGACCAAAGG
TCGCCCGACGCCCGGGCTTTGCCCGGGCGGCCTCAGTGAGCGAGCGAGCGCGCAG

Supplementary Sequence 8. Single-AAV enCjCas9-TadCBEd with *PRNP* Q91X F-sgRNA.

ITR-EFS promoter-NLS-TadCBEd deaminase domain-linker 1-enCjCas9-linker 2-UGI-NLS-SV40 poly(A)-*PRNP* Q91X F-sgRNA (reverse complement)-human U6 promoter (reverse complement)-ITR

CTGCGCGCTCGCTCGCTCACTGAGGCCGCCCGGGCAAAGCCCGGGCGTCGGGCGACC
TTTGGTCGCCCGGCCTCAGTGAGCGAGCGAGCGCGCAGAGAGGGAGTGGCCAACTCC
ATCACTAGGGGTTCCTGCGGCCTCTAGATAGGTCTTGAAAGGAGTGGGAATTGGCTCCG
GTGCCCGTCAGTGGGCAGAGCGCACATCGCCCACAGTCCCCGAGAAGTTGGGGGGAG
GGGTCGGCAATTGATCCGGTGCCTAGAGAAGGTGGCGCGGGGTAAACTGGGAAAGTGA
TGTCGTGTACTGGCTCCGCCTTTTTCCCGAGGGTGGGGGAGAACCGTATATAAGTGCAG
TAGTCGCCGTGAACGTTCTTTTTCGCAACGGGTTTGCCGCCAGAACACAGGACCGGTGC
CACCATGAAACGGACAGCCGACGGAAGCGAGTTCGAGTCACCAAAGAAGAAGCGGAAA
GTCTCTGAGGTGGAGTTTTCCCACGAGTACTGGATGAGACATGCCCTGACCCTGGCCAA
GAGGGCACGGGATGAGAGGAAGGCGCCTGTGGGAGCCGTGCTGGTGCTGAACAATAG
AGTGATCGGCGAGGGCTGGAACAGAGCCATCGGCCTGCACGACCCAACAGCCCATGCC
GAAATTATAGCCCTGAGACAGGGCGGCCTGGTCATGCAGAACTACAGACTGATTGACGC
CACCCTGTACGTGACATTCGAGCCTTGCGTGATGTGCGCCGGCGCCATGATCAACTCTA
GGATCGGCCGCGTGGTGTTTGGCGTGAGGAACTCAAAAAGAGGCGCCGCAGGCTCCCT
GATGAACGTGCTGAACTACCCCGGAATGAATCACCGCGTCGAAATTACCGAGGGAATCC
TGGCAGATGAATGTGCCGCCCTGCTGTGCGATTTCTATCGGATGCCTAGACAGGTGTTCA
ATGCTCAGAAGAAGGCCCAGAGCTCCATCAACTCTGGCGGATCTAGCGGAGGATCCTCT
GGCAGCGAGACACCAGGAACAAGCGAGTCAGCAACACCAGAGAGCAGTGGCGGCAGC
AGCGGCGGCAGCGCCCGCATCCTCGCTTTCGCAATCGGAATCTCTAGTATCGGATGGGC
CTTCTCTGAAAACGACGAACTGAAAGACTGCGGCGTGAGAATCTTCACAAAGGTTGAAA
ACCCTAAAACAGGCGAGTCTTTAGCTCTGCCACGTAGGTTGGCCCGCTCCGCCCGAAAA
AGGTATGCTCGGCGGAAGGCTCGCCTCAACCACTTGAAGCATTTGATAGCTAATGAGTTC
AAACTGAACTACGAAGATTACCAGTCCTTCGACGAGTCATTGGCAAAAGCCTACAAAGGC
AGCCTTATCAGTCCTTATGAGTTGAGATTTCGCGCACTCAACGAACTGCTTTCTAAGCAG
ACTTTGCTAGGGTCATTCTGCACATCGCAAAACGGCGAGGTTATGACGATATCAAGAACT
CCGACGATAAAGAAAAGGGAGCCATTCTCAAGGCGATCAAACAGAATGAGGAAAAATTG
GCAAACTACCAGAGTGTGGGCGAGTATCTGTATAAAGAGTATTTCCAGAAGTTTAAGGAAA

ACAGCAAGGAGTTTACAAACGTCAGAAATAAAAAGGAGTCTTACGAGAGATGCATCGCGC
AGTCATTCCTCAAAGATGAGCTGAAGCTGATATTTAAGAAGCAACGCGAATTTGGTTTCTC
ATTCTCTAAGAAGTTCGAAGAGGAGGTTCTTTCCGTGGCGTTTTACAAGAGGGCGCTCAA
AGACTTCTCCCACCTGGTTGGTAACTGTAGTTTCTTCACGGATGAGAAGCGAGCTCCCAA
AAATTCTCCCCTGGCTTTCATGTTTGTTGCCCTGACTCGGATCATTAACCTGCTGAACAAC
CTGAAAAATACTGAAGGGATCTTGTATACGAAGGACGACCTAAATGCACTCCTGAATGAA
GTGCTCAAAAACGGAACTCTAACCTATAAACAGACCAAGAAATTACTGGGGCTCTCTGAC
GACTACGAGTTCAAGGGCGAGAAGGGTACTTATTTTATCGAATTCAAAAAGTATAAGGAGT
TCATTAAAGCATTGGGGGAACACAACCTCAGCCAGGACGATCTCAATGAAATTGCCAAGG
ACATCACGCTGATTAAAGACGAGATAAAACTGAAAAAGGCACTGGCCAAGTATGACCTCA
ACCAGAACCAGATCGACTCTCTGTCCAAGCTGGAGTTCAAAGACCACCTAAACATATCCT
TCAAAGCCCTGAAACTGGTCACCCCTCTAATGCTCGAAGGAAAAAAATACGACGAGGCG
TGTAATGAACTGAATCTTAAGGTGGCCATCAATGAGGATAAGAAGGACTTTCTTCCAGCCT
TTAACGAGACATATTACAAAGACGAGGTCACAAACCCGGTTGTGCTGAGGGCCATAAAAG
AGTATCGGAAGGTTCTGAATGCCCTCCTGAAGAAGTACGGCAAAGTGCACAAAATAAATA
TCGAATTGGCTAGGGAGGTGGGGAAGAACCATTCTCAGCGAGCAAAGATCGAGAAAGAG
CAGAATGAGAACTACAAAGCCAAGAAAGACGCCGAACTGGAGTGCGAAAAGCTGGGGC
TTAAAATAAACAGTAAAAACATCCTGAAATTAAGATTGTTCAAAGAGCAAAAGGAGTTTTGC
GCCTACTCAGGGGAAAAAATCAAAATATCAGACCTGCAGGACGAGAAAATGCTGGAGATC
GACCATATCTATCCGTATAGCAGGTCATTTGACGATTCCTACATGAACAAAGTGCTTGTGTT
TACCAAACAGAACCAAGAAAAGCTGAACCAAACCCCCTTTGAGGCTTTCGGAAACGACT
CAGCCAAGTGGCAGAAAATCGAAGTCCTAGCCAAGAATCTGCCTACAAAAAAACAAAAGA
GGATTCTTGATAAGAACTATAAGGACAAGGAACAGAAAAACTTTAAAGACAGGAACCTGA
ATGACACGAGGTACATTGCGCGACTGGTTCTAAACTATACCAAAGACTACCTGGATTTCCT
CCCTCTGAGCGACGACGAGAATACTAAACTGAATGATACCCAGAAAGGCTCAAAGGTCCA
CGTTGAGGCTAAGTCCGGGATGCTGACTAGCGCCCTCCGCCACACGTGGGGCTTCAGC
GCCAAAGATCGGAATAATCATCTTCATCACGCTATTGATGCAGTAATCATAGCCTACGCTAA
CAACAGCATCGTGAAAGCCTTCTCCGATTTCAAGAAAGAACAGGAGTCTAATAGCGCCGA
GTTGTACGCCAAGAAAATTTCCGAATTGGACTATAAAAATAAGAGAAAATTCTTCGAACCC
TTCTCCGGGTTTCGCCAAAAGGTCTTAGATAAGATCGACGAGATTTTCGTTTCCAAGCCC
GAAAGAAAAAAGCCTTCAGGGGCACTGCACGAAGAGACATTCCGCAAGGAAGAGGAATT
TTACCAATCTTACGGTGGTAAAGAGGGAGTTCTGAAGGCTCTGGAGCTTGGGAAGATCC
GCAAGGTAAACGGGAAAATCGTGAAAAACGGGGACATGTTCAGGGTGGATATCTTCAAG

CACAAAAAGACCAACAAGTTCTACGCAGTACCCATCTACACTATGGATTTCGCTTTAAAGG
TTCTCCCAAATAAGGCGGTGGCTCGATCGAAGAAAGGAGAGATCAAGGACTGGATCTTAA
TGGATGAAAATTACGAGTTTTGCTTCTCGCTCTACAAAGATAGCCTGATTCTGATCCAGAC
AAAAAAGATGCAGGAACCAGAATTTGTTTATTATAACGCCTTCACGAGCAGTACAGTGTCC
CTGATTGTGAGCAAGCATGATAACAAGTTCGAGACTCTGTCTAAGAATCAGAAAATCCTTT
TCAAGAACGCCAACGAGAAGGAGGTCATCGCAAAGTCAATTGGCATCCAAAACCTGAAG
GTGTTCGAGAAATACATAGTGTCCGCACTCGGTGAAGTAACTAAAGCCGAATTTCGACAG
CGCGAGGATTTTAAGAAAAGCGGCGGGAGCGGCGGGAGCGGGGGGAGCACTAATCTG
AGCGACATCATTGAGAAGGAGACTGGGAAACAGCTGGTCATTCAGGAGTCCATCCTGAT
GCTGCCTGAGGAGGTGGAGGAAGTGATCGGCAACAAGCCAGAGTCTGACATCCTGGTG
CACACCGCCTACGACGAGTCCACAGATGAGAATGTGATGCTGCTGACCTCTGACGCCCC
CGAGTATAAGCCTTGGGCCCTGGTCATCCAGGATTCTAACGGCGAGAATAAGATCAAGAT
GCTGAGCGGAGGATCCAAAAGAACCGCCGACGGCAGCGAATTCGAGCCCAAGAAGAAG
AGGAAAGTCTAATAGATCTCTTTATTTGTGAAATTTGTGATGCTATTGCTTTATTTGTAACCA
TTATAAGCTGCAATAAACAAGTTAACAACAACAATTGCATTCATTTTATGTTTCAGGTTCAG
GGGGAGATGTGGGAGGTTTTTTAAAGCAAGCTTAAAAAAGCGGTTTTAGGGGATTGTAAC
CCCGCAGAGTCCCGCAAACTCTTTATTATAGTCCCTTTTCAGGGACTATAACTCCTTGACC
CCAGCCACCACCGGTGTTTCGTCCTTTCCACAAGATATATAAAGCCAAGAAATCGAAATAC
TTTCAAGTTACGGTAAGCATATGATAGTCCATTTTAAAACATAATTTTAAAACTGCAAACTAC
CCAAGAAATTATTACTTTCTACGTCACGTATTTTGTACTAATATCTTTGTGTTTACAGTCAAA
TTAATTCTAATTATCTCTCTAACAGCCTTGTATCGTATATGCAAATATGAAGGAATCATGGGA
AATAGGCCCTCTTCCTGCCCGACCTTGCGGCCGCAGGAACCCCTAGTGATGGAGTTGGC
CACTCCCTCTCTGCGCGCTCGCTCGCTCACTGAGGCCGGGCGACCAAAGGTCGCCCGA
CGCCCGGGCTTTGCCCGGGCGGCCTCAGTGAGCGAGCGAGCGCGCAG

Supplementary Sequence 9. Dual-AAV SpCas9-ABE8e(V106W) *PRNP* M1V F+E-sgRNA (N-terminus).

ITR-Cbh promoter-NLS-ABE8e(V106W) deaminase domain-linker-SpCas9 (amino acids 1-572)-NpuN-NLS-WPRE-bovine growth hormone(bGH)-derived poly(A)-*PRNP* M1V F+E-sgRNA (reverse complement)-human U6 promoter (reverse complement)-ITR

CTGCGCGCTCGCTCGCTCACTGAGGCCGCCCGGGCAAAGCCCGGGCGTCGGGCGACC
TTTGGTCGCCCGGCCTCAGTGAGCGAGCGAGCGCGCAGAGAGGGAGTGGCCAACTCC
ATCACTAGGGGTTCCTGCGGCCTCTAGATCAGGGTACCCGTTACATAACTTACGGTAAAT
GGCCCGCCTGGCTGACCGCCCAACGACCCCCGCCCATTGACGTCAATAGTAACGCCAAT
AGGGACTTTCCATTGACGTCAATGGGTGGAGTATTTACGGTAAACTGCCCACTTGGCAGT
ACATCAAGTGTATCATATGCCAAGTACGCCCCCTATTGACGTCAATGACGGTAAATGGCCC
GCCTGGCATTGTGCCCAGTACATGACCTTATGGGACTTTCCTACTTGGCAGTACATCTAC
GTATTAGTCATCGCTATTACCATGGTCGAGGTGAGCCCCACGTTCTGCTTCACTCTCCCC
ATCTCCCCCCCCTCCCCACCCCCAATTTTGTATTTATTTATTTTTTAATTATTTTGTGCAGCG
ATGGGGGCGGGGGGGGGGGGGGGGCGCGCGCCAGGCGGGGCGGGGCGGGGCGAG
GGGCGGGGCGGGGCGAGGCGGAGAGGTGCGGCGGCAGCCAATCAGAGCGGCGCGCT
CCGAAAGTTTCCTTTTATGGCGAGGCGGCGGCGGCGGCGGCCCTATAAAAAGCGAAGC
GCGCGGCGGGCGGGAGTCGCTGCGACGCTGCCTTCGCCCCGTGCCCCGCTCCGCCG
CCGCCTCGCGCCGCCCGCCCCGGCTCTGACTGACCGCGTTACTCCCACAGGTGAGCG
GGCGGGACGGCCCTTCTCCTCCGGGCTGTAATTAGCTGAGCAAGAGGTAAGGGTTTAAG
GGATGGTTGGTTGGTGGGGTATTAATGTTTAATTACCTGGAGCACCTGCCTGAAATCACTT
TTTTTCAGGTTGGACCGGTGCCACCATGAAACGGACAGCCGACGGAAGCGAGTTCGAG
TCACCAAAGAAGAAGCGGAAAGTCTCTGAGGTGGAGTTTTCCCACGAGTACTGGATGAG
ACATGCCCTGACCCTGGCCAAGAGGGCACGGGATGAGAGGGAGGTGCCTGTGGGAGC
CGTGCTGGTGCTGAACAATAGAGTGATCGGCGAGGGCTGGAACAGAGCCATCGGCCTG
CACGACCCAACAGCCCATGCCGAAATTATGGCCCTGAGACAGGGCGGCCTGGTCATGC
AGAACTACAGACTGATTGACGCCACCCTGTACGTGACATTCGAGCCTTGCGTGATGTGC
GCCGGCGCCATGATCCACTCTAGGATCGGCCGCGTGGTGTTTGGATGGAGAAATTCTAA
AAGAGGCGCCGCAGGCTCCCTGATGAACGTGCTGAACTACCCCGGCATGAATCACCGC
GTCGAAATTACCGAGGGAATCCTGGCAGATGAATGTGCCGCCCTGCTGTGCGATTTCTAT
CGGATGCCTAGACAGGTGTTCAATGCTCAGAAGAAGGCCCAGAGCTCCATCAACTCCGG
AGGATCTAGCGGAGGCTCCTCTGGCTCTGAGACACCTGGCACAAGCGAGAGCGCAACA

CCTGAAAGCAGCGGGGGCAGCAGCGGGGGGTCAGACAAGAAGTACAGCATCGGCCTG
GCCATCGGCACCAACTCTGTGGGCTGGGCCGTGATCACCGACGAGTACAAGGTGCCCA
GCAAGAAATTCAAGGTGCTGGGCAACACCGACCGGCACAGCATCAAGAAGAACCTGATC
GGAGCCCTGCTGTTCGACAGCGGCGAAACAGCCGAGGCCACCCGGCTGAAGAGAACC
GCCAGAAGAAGATACACCAGACGGAAGAACCGGATCTGCTATCTGCAAGAGATCTTCAG
CAACGAGATGGCCAAGGTGGACGACAGCTTCTTCCACAGACTGGAAGAGTCCTTCCTG
GTGGAAGAGGATAAGAAGCACGAGCGGCACCCCATCTTCGGCAACATCGTGGACGAGG
TGGCCTACCACGAGAAGTACCCCACCATCTACCACCTGAGAAAGAAACTGGTGGACAGC
ACCGACAAGGCCGACCTGCGGCTGATCTATCTGGCCCTGGCCCACATGATCAAGTTCCG
GGGCCACTTCCTGATCGAGGGCGACCTGAACCCCGACAACAGCGACGTGGACAAGCTG
TTCATCCAGCTGGTGCAGACCTACAACCAGCTGTTCGAGGAAAACCCCATCAACGCCAG
CGGCGTGGACGCCAAGGCCATCCTGTCTGCCAGACTGAGCAAGAGCAGACGGCTGGAA
AATCTGATCGCCCAGCTGCCCGGCGAGAAGAAGAATGGCCTGTTCGGAAACCTGATTGC
CCTGAGCCTGGGCCTGACCCCCAACTTCAAGAGCAACTTCGACCTGGCCGAGGATGCC
AAACTGCAGCTGAGCAAGGACACCTACGACGACGACCTGGACAACCTGCTGGCCCAGA
TCGGCGACCAGTACGCCGACCTGTTTCTGGCCGCCAAGAACCTGTCCGACGCCATCCT
GCTGAGCGACATCCTGAGAGTGAACACCGAGATCACCAAGGCCCCCCTGAGCGCCTCT
ATGATCAAGAGATACGACGAGCACCACCAGGACCTGACCCTGCTGAAAGCTCTCGTGCG
GCAGCAGCTGCCTGAGAAGTACAAAGAGATTTTCTTCGACCAGAGCAAGAACGGCTACG
CCGGCTACATTGACGGCGGAGCCAGCCAGGAAGAGTTCTACAAGTTCATCAAGCCCATC
CTGGAAAAGATGGACGGCACCGAGGAACTGCTCGTGAAGCTGAACAGAGAGGACCTGC
TGCGGAAGCAGCGGACCTTCGACAACGGCAGCATCCCCCACCAGATCCACCTGGGAGA
GCTGCACGCCATTCTGCGGCGGCAGGAAGATTTTTACCCATTCCTGAAGGACAACCGGG
AAAAGATCGAGAAGATCCTGACCTTCCGCATCCCCTACTACGTGGGCCCTCTGGCCAGG
GGAAACAGCAGATTCGCCTGGATGACCAGAAAGAGCGAGGAAACCATCACCCCCTGGA
ACTTCGAGGAAGTGGTGGACAAGGGCGCTTCCGCCCAGAGCTTCATCGAGCGGATGAC
CAACTTCGATAAGAACCTGCCCAACGAGAAGGTGCTGCCCAAGCACAGCCTGCTGTACG
AGTACTTCACCGTGTATAACGAGCTGACCAAAGTGAAATACGTGACCGAGGGAATGAGAA
AGCCCGCCTTCCTGAGCGGCGAGCAGAAAAAGGCCATCGTGGACCTGCTGTTCAAGAC
CAACCGGAAAGTGACCGTGAAGCAGCTGAAAGAGGACTACTTCAAGAAAATCGAGTGCC
TGTCCTACGAGACAGAGATCCTGACAGTGGAGTATGGCCTGCTGCCAATCGGCAAGATC
GTGGAGAAGAGGATCGAGTGTACCGTGTACTCTGTGGATAACAATGGCAACATCTATACA
CAGCCCGTGGCACAGTGGCACGATAGGGGAGAGCAGGAGGTGTTCGAGTATTGCCTGG

AGGACGGCAGCCTGATCAGGGCAACCAAGGACCACAAGTTCATGACAGTGGATGGCCA
GATGCTGCCCATCGACGAGATTTTCGAGCGGGAGCTGGACCTGATGAGAGTGGATAACC
TGCCTAATAGCGGAGGCAGTAAAAGAACAGCAGACGGGAGTGAGTTTGAGCCCAAGAAA
AAGAGAAAGGTGTAAGATCTGATAATCAACCTCTGGATTACAAAATTTGTGAAAGATTGAC
TGGTATTCTTAACTATGTTGCTCCTTTTACGCTATGTGGATACGCTGCTTTAATGCCTTTGT
ATCATGCTATTGCTTCCCGTATGGCTTTCATTTTCTCCTCCTTGTATAAATCCTGGTTAGTT
CTTGCCACGGCGGAACTCATCGCCGCCTGCCTTGCCCGCTGCTGGACAGGGGCTCGG
CTGTTGGGCACTGACAATTCCGTGGTGCGACTGTGCCTTCTAGTTGCCAGCCATCTGTT
GTTTGCCCCTCCCCCGTGCCTTCCTTGACCCTGGAAGGTGCCACTCCCACTGTCCTTTC
CTAATAAAATGAGGAAATTGCATCGCATTGTCTGAGTAGGTGTCATTCTATTCTGGGGGGT
GGGGTGGGGCAGGACAGCAAGGGGGAGGATTGGGAAGACAATAGCAGGCATGCTGGG
GATGCGGTGGGCTCTATGGCTCGAGAAAAAAAGCACCGACTCGGTGCCACTTTTTCAAG
TTGATAACGGACTAGCCTTATTTAAACTTGCTATGCTGTTTCCAGCATAGCTCTTAAACGCA
GCCAAGGTTCGCCATAACGGTGTTTCGTCCTTTCCACAAGATATATAAAGCCAAGAAATCG
AAATACTTTCAAGTTACGGTAAGCATATGATAGTCCATTTTAAAACATAATTTTAAAACTGCA
AACTACCCAAGAAATTATTACTTTCTACGTCACGTATTTTGTACTAATATCTTTGTGTTTACA
GTCAAATTAATTCTAATTATCTCTCTAACAGCCTTGTATCGTATATGCAAATATGAAGGAATC
ATGGGAAATAGGCCCTCTTCCTGCCCGACCTTGCGGCCGCAGGAACCCCTAGTGATGGA
GTTGGCCACTCCCTCTCTGCGCGCTCGCTCGCTCACTGAGGCCGGGCGACCAAAGGTC
GCCCGACGCCCGGGCTTTGCCCGGGCGGCCTCAGTGAGCGAGCGAGCGCGCAG

Supplementary Sequence 10. Dual-AAV SpCas9-ABE8e(V106W) *PRNP* M1V F+E-sgRNA (C-terminus).

ITR-Cbh promoter-NLS-NpuC-SpCas9 (amino acids 573-1367)-NLS-WPRE-bovine growth hormone(bGH)-derived poly(A)-*PRNP* M1V F+E-sgRNA (reverse complement)-human U6 promoter (reverse complement)-ITR

CTGCGCGCTCGCTCGCTCACTGAGGCCGCCCGGGCAAAGCCCGGGCGTCGGGCGACC
TTTGGTCGCCCGGCCTCAGTGAGCGAGCGAGCGCGCAGAGAGGGAGTGGCCAACTCC
ATCACTAGGGGTTCCTGCGGCCTCTAGATCAGGGTACCCGTTACATAACTTACGGTAAAT
GGCCCGCCTGGCTGACCGCCCAACGACCCCCGCCCATTGACGTCAATAGTAACGCCAAT
AGGGACTTTCCATTGACGTCAATGGGTGGAGTATTTACGGTAAACTGCCCACTTGGCAGT
ACATCAAGTGTATCATATGCCAAGTACGCCCCCTATTGACGTCAATGACGGTAAATGGCCC
GCCTGGCATTGTGCCCAGTACATGACCTTATGGGACTTTCCTACTTGGCAGTACATCTAC
GTATTAGTCATCGCTATTACCATGGTCGAGGTGAGCCCCACGTTCTGCTTCACTCTCCCC
ATCTCCCCCCCCTCCCCACCCCCAATTTTGTATTTATTTATTTTTTAATTATTTTGTGCAGCG
ATGGGGGCGGGGGGGGGGGGGGGGCGCGCGCCAGGCGGGGCGGGGCGGGGCGAG
GGGCGGGGCGGGGCGAGGCGGAGAGGTGCGGCGGCAGCCAATCAGAGCGGCGCGCT
CCGAAAGTTTCCTTTTATGGCGAGGCGGCGGCGGCGGCGGCCCTATAAAAAGCGAAGC
GCGCGGCGGGCGGGAGTCGCTGCGACGCTGCCTTCGCCCCGTGCCCCGCTCCGCCG
CCGCCTCGCGCCGCCCGCCCCGGCTCTGACTGACCGCGTTACTCCCACAGGTGAGCG
GGCGGGACGGCCCTTCTCCTCCGGGCTGTAATTAGCTGAGCAAGAGGTAAGGGTTTAAG
GGATGGTTGGTTGGTGGGGTATTAATGTTTAATTACCTGGAGCACCTGCCTGAAATCACTT
TTTTTCAGGTTGGACCGGTGCCACCATGAAACGGACAGCCGACGGAAGCGAGTTCGAG
TCACCAAAGAAGAAGCGGAAAGTCATCAAGATTGCTACACGGAAATACCTGGAAAGCA
GAACGTGTACGACATCGGCGTGGAGCGGGATCACAACTTCGCCCTGAAGAATGGCTTTA
TCGCCAGCAATTGCTTCGACTCCGTGGAAATCTCCGGCGTGGAAGATCGGTTCAACGCC
TCCCTGGGCACATACCACGATCTGCTGAAAATTATCAAGGACAAGGACTTCCTGGACAAT
GAGGAAAACGAGGACATTCTGGAAGATATCGTGCTGACCCTGACACTGTTTGAGGACAG
AGAGATGATCGAGGAACGGCTGAAAACCTATGCCCACCTGTTCGACGACAAAGTGATGA
AGCAGCTGAAGCGGCGGAGATACACCGGCTGGGGCAGGCTGAGCCGGAAGCTGATCA
ACGGCATCCGGGACAAGCAGTCCGGCAAGACAATCCTGGATTTCCTGAAGTCCGACGG
CTTCGCCAACAGAAACTTCATGCAGCTGATCCACGACGACAGCCTGACCTTTAAAGAGG
ACATCCAGAAAGCCCAGGTGTCCGGCCAGGGCGATAGCCTGCACGAGCACATTGCCAAT

CTGGCCGGCAGCCCCGCCATTAAGAAGGGCATCCTGCAGACAGTGAAGGTGGTGGACG
AGCTCGTGAAAGTGATGGGCCGGCACAAGCCCGAGAACATCGTGATCGAAATGGCCAG
AGAGAACCAGACCACCCAGAAGGGACAGAAGAACAGCCGCGAGAGAATGAAGCGGATC
GAAGAGGGCATCAAAGAGCTGGGCAGCCAGATCCTGAAAGAACACCCCGTGGAAAACA
CCCAGCTGCAGAACGAGAAGCTGTACCTGTACTACCTGCAGAATGGGCGGGATATGTAC
GTGGACCAGGAACTGGACATCAACCGGCTGTCCGACTACGATGTGGACCATATCGTGCC
TCAGAGCTTTCTGAAGGACGACTCCATCGACAACAAGGTGCTGACCAGAAGCGACAAGA
ACCGGGGCAAGAGCGACAACGTGCCCTCCGAAGAGGTCGTGAAGAAGATGAAGAACTA
CTGGCGGCAGCTGCTGAACGCCAAGCTGATTACCCAGAGAAAGTTCGACAATCTGACCA
AGGCCGAGAGAGGCGGCCTGAGCGAACTGGATAAGGCCGGCTTCATCAAGAGACAGCT
GGTGGAAACCCGGCAGATCACAAAGCACGTGGCACAGATCCTGGACTCCCGGATGAAC
ACTAAGTACGACGAGAATGACAAGCTGATCCGGGAAGTGAAAGTGATCACCCTGAAGTC
CAAGCTGGTGTCCGATTTCCGGAAGGATTTCCAGTTTTACAAAGTGCGCGAGATCAACAA
CTACCACCACGCCCACGACGCCTACCTGAACGCCGTCGTGGGAACCGCCCTGATCAAA
AAGTACCCTAAGCTGGAAAGCGAGTTCGTGTACGGCGACTACAAGGTGTACGACGTGCG
GAAGATGATCGCCAAGAGCGAGCAGGAAATCGGCAAGGCTACCGCCAAGTACTTCTTCT
ACAGCAACATCATGAACTTTTTCAAGACCGAGATTACCCTGGCCAACGGCGAGATCCGG
AAGCGGCCTCTGATCGAGACAAACGGCGAAACCGGGGAGATCGTGTGGGATAAGGGCC
GGGATTTTGCCACCGTGCGGAAAGTGCTGAGCATGCCCCAAGTGAATATCGTGAAAAAG
ACCGAGGTGCAGACAGGCGGCTTCAGCAAAGAGTCTATCCTGCCCAAGAGGAACAGCG
ATAAGCTGATCGCCAGAAAGAAGGACTGGGACCCTAAGAAGTACGGCGGCTTCGACAGC
CCCACCGTGGCCTATTCTGTGCTGGTGGTGGCCAAAGTGGAAAAGGGCAAGTCCAAGA
AACTGAAGAGTGTGAAAGAGCTGCTGGGGATCACCATCATGGAAAGAAGCAGCTTCGAG
AAGAATCCCATCGACTTTCTGGAAGCCAAGGGCTACAAAGAAGTGAAAAAGGACCTGAT
CATCAAGCTGCCTAAGTACTCCCTGTTCGAGCTGGAAAACGGCCGGAAGAGAATGCTGG
CCTCTGCCGGCGAACTGCAGAAGGGAAACGAACTGGCCCTGCCCTCCAAATATGTGAAC
TTCCTGTACCTGGCCAGCCACTATGAGAAGCTGAAGGGCTCCCCCGAGGATAATGAGCA
GAAACAGCTGTTTGTGGAACAGCACAAGCACTACCTGGACGAGATCATCGAGCAGATCA
GCGAGTTCTCCAAGAGAGTGATCCTGGCCGACGCTAATCTGGACAAAGTGCTGTCCGCC
TACAACAAGCACCGGGATAAGCCCATCAGAGAGCAGGCCGAGAATATCATCCACCTGTTT
ACCCTGACCAATCTGGGAGCCCCTGCCGCCTTCAAGTACTTTGACACCACCATCGACCG
GAAGAGGTACACCAGCACCAAAGAGGTGCTGGACGCCACCCTGATCCACCAGAGCATC
ACCGGCCTGTACGAGACACGGATCGACCTGTCTCAGCTGGGAGGTGACAAAAGAACCG

CCGACGGCAGCGAATTCGAGCCCAAGAAGAAGAGGAAAGTCTAAGATCTGATAATCAAC
CTCTGGATTACAAAATTTGTGAAAGATTGACTGGTATTCTTAACTATGTTGCTCCTTTTACG
CTATGTGGATACGCTGCTTTAATGCCTTTGTATCATGCTATTGCTTCCCGTATGGCTTTCAT
TTTCTCCTCCTTGTATAAATCCTGGTTAGTTCTTGCCACGGCGGAACTCATCGCCGCCTG
CCTTGCCCGCTGCTGGACAGGGGCTCGGCTGTTGGGCACTGACAATTCCGTGGTGCGA
CTGTGCCTTCTAGTTGCCAGCCATCTGTTGTTTGCCCCTCCCCCGTGCCTTCCTTGACCC
TGGAAGGTGCCACTCCCACTGTCCTTTCCTAATAAAATGAGGAAATTGCATCGCATTGTCT
GAGTAGGTGTCATTCTATTCTGGGGGGTGGGGTGGGGCAGGACAGCAAGGGGGAGGAT
TGGGAAGACAATAGCAGGCATGCTGGGGATGCGGTGGGCTCTATGGCTCGAGGCACCG
ACTCGGTGCCACTTTTTCAAGTTGATAACGGACTAGCCTTATTTAAACTTGCTATGCTGTTT
CCAGCATAGCTCTTAAACGCAGCCAAGGTTCGCCATAACGGTGTTTCGTCCTTTCCACAA
GATATATAAAGCCAAGAAATCGAAATACTTTCAAGTTACGGTAAGCATATGATAGTCCATTTT
AAAACATAATTTTAAAACTGCAAACTACCCAAGAAATTATTACTTTCTACGTCACGTATTTTG
TACTAATATCTTTGTGTTTACAGTCAAATTAATTCTAATTATCTCTCTAACAGCCTTGTATCGT
ATATGCAAATATGAAGGAATCATGGGAAATAGGCCCTCTTCCTGCCCGACCTTGCGGCCG
CAGGAACCCCTAGTGATGGAGTTGGCCACTCCCTCTCTGCGCGCTCGCTCGCTCACTGA
GGCCGGGCGACCAAAGGTCGCCCGACGCCCGGGCTTTGCCCGGGCGGCCTCAGTGAG
CGAGCGAGCGCGCAG

Supplementary Sequence 11. Single-AAV SauriCas9-ABE8e with *PRNP* M1V F-sgRNA.

ITR-EFS promoter-NLS-ABE8e(V106W) deaminase domain-linker-SauriCas9-NLS-bovine growth hormone(bGH)-derived poly(A)-*PRNP* M1V F-sgRNA (reverse complement)-human U6 promoter (reverse complement)-ITR

CTGCGCGCTCGCTCGCTCACTGAGGCCGCCCGGGCAAAGCCCGGGCGTCGGGCGACC
TTTGGTCGCCCGGCCTCAGTGAGCGAGCGAGCGCGCAGAGAGGGAGTGGCCAACTCC
ATCACTAGGGGTTCCTGCGGCCTCTAGAATTCGCTAGCTAGGTCTTGAAAGGAGTGGGA
ATTGGCTCCGGTGCCCGTCAGTGGGCAGAGCGCACATCGCCCACAGTCCCCGAGAAGT
TGGGGGGAGGGGTCGGCAATTGATCCGGTGCCTAGAGAAGGTGGCGCGGGGTAAACT
GGGAAAGTGATGTCGTGTACTGGCTCCGCCTTTTTCCCGAGGGTGGGGGAGAACCGTAT
ATAAGTGCAGTAGTCGCCGTGAACGTTCTTTTTCGCAACGGGTTTGCCGCCAGAACACA
GGACCGGTGCCACCATGAAACGGACAGCCGACGGAAGCGAGTTCGAGTCACCAAAGAA
GAAGCGGAAAGTCTCTGAGGTGGAGTTTTCCCACGAGTACTGGATGAGACATGCCCTGA
CCCTGGCCAAGAGGGCACGGGATGAGAGGGAGGTGCCTGTGGGAGCCGTGCTGGTGC
TGAACAATAGAGTGATCGGCGAGGGCTGGAACAGAGCCATCGGCCTGCACGACCCAAC
AGCCCATGCCGAAATTATGGCCCTGAGACAGGGCGGCCTGGTCATGCAGAACTACAGAC
TGATTGACGCCACCCTGTACGTGACATTCGAGCCTTGCGTGATGTGCGCCGGCGCCATG
ATCCACTCTAGGATCGGCCGCGTGGTGTTTGGCGTGAGGAACTCAAAAAGAGGCGCCG
CAGGCTCCCTGATGAACGTGCTGAACTACCCCGGCATGAATCACCGCGTCGAAATTACC
GAGGGAATCCTGGCAGATGAATGTGCCGCCCTGCTGTGCGATTTCTATCGGATGCCTAG
ACAGGTGTTCAATGCTCAGAAGAAGGCCCAGAGCTCCATCAACTCCGGAGGATCTAGCG
GAGGCTCCTCTGGCTCTGAGACACCTGGCACAAGCGAGAGCGCAACACCTGAAAGCAG
CGGGGGCAGCAGCGGGGGGTCACAGGAGAACCAGCAGAAGCAAAATTACATCCTGGGC
CTGGCCATCGGCATCACCAGCGTGGGCTATGGCCTGATCGACAGCAAGACCAGAGAAG
TGATTGACGCCGGCGTGCGGCTATTCCCAGAGGCCGACTCTGAAAACAACAGCAATAGA
AGATCTAAGCGGGGCGCCCGGAGACTGAAAAGACGAAGAATCCACAGACTGAACAGAG
TGAAAGACCTCCTGGCTGACTACCAGATGATCGACTTAAACAACGTGCCCAAGTCTACCG
ACCCCTACACCATCCGGGTGAAGGGACTGCGGGAACCTCTGACCAAGGAAGAGTTTGC
CATCGCTCTGCTGCATATCGCCAAGAGAAGAGGCCTGCACAACATCTCCGTGAGCATGG
GCGATGAGGAACAGGACAACGAGCTGTCCACCAAGCAGCAGCTGCAGAAGAACGCTCA
GCAGCTGCAGGACAAATACGTGTGCGAGCTGCAACTGGAAAGACTGACCAACATCAACA
AGGTCAGAGGCGAGAAGAACCGGTTCAAGACCGAAGATTTCGTGAAGGAAGTGAAGCA
GCTGTGCGAGACCCAGCGGCAGTACCACAACATCGACGATCAGTTCATCCAGCAGTACA
TCGACCTGGTGAGCACCCGGAGAGAATACTTCGAGGGCCCTGGCAACGGCTCTCCATAT
GGCTGGGATGGAGATCTGCTGAAGTGGTATGAGAAGCTGATGGGCAGATGCACCTACTT
CCCTGAGGAGCTGAGAAGCGTGAAGTACGCCTACAGCGCCGATCTGTTTAACGCCCTGA
ACGATCTGAACAACCTGGTTGTGACCCGCGACGACAACCCTAAGCTGGAATACTACGAG
AAATACCACATTATCGAGAACGTGTTCAAGCAGAAGAAAAATCCAACTCTGAAGCAAATCG
CCAAAGAGATCGGCGTGCAGGATTACGACATCAGAGGATACAGAATTACCAAGTCCGGTA
AGCCTCAGTTCACCAGCTTCAAACTCTACCACGACCTGAAAAACATCTTTGAACAGGCCA
AATACCTGGAAGATGTGGAGATGCTGGATGAGATAGCTAAAATCCTGACAATCTACCAAGA
CGAGATCAGCATCAAGAAAGCCCTGGACCAGCTGCCTGAGCTGCTGACCGAGAGCGAA
AAAAGCCAGATCGCTCAGCTGACCGGCTACACCGGTACACATAGACTGTCTCTGAAGTG
CATCCACATCGTGATCGACGAGCTGTGGGAGAGCCCCGAAAACCAGATGGAAATCTTCA
CCAGACTGAACCTCAAGCCAAAGAAGGTGGAAATGAGCGAGATCGACAGCATCCCTACC
ACACTGGTGGATGAGTTCATCCTGAGCCCTGTGGTGAAGCGGGCCTTCATCCAGTCCAT

CAAGGTGATCAACGCTGTGATCAACAGATTCGGCCTGCCCGAGGACATCATTATCGAGCT
GGCCAGAGAGAAGAACAGCAAGGACAGAAGGAAGTTCATCAACAAGCTGCAGAAACAG
AATGAGGCCACCCGGAAAAAAATCGAGCAGCTGCTGGCCAAGTACGGCAATACCAATGC
CAAGTACATGATCGAAAAGATCAAGCTGCATGACATGCAGGAGGGCAAGTGTCTGTACAG
CCTGGAAGCTATCCCCCTGGAAGACCTGCTGTCTAATCCTACACACTACGAGGTGGACC
ACATCATCCCTAGAAGCGTGTCCTTCGACAACAGCCTGAACAACAAGGTTCTGGTGAAG
CAAAGCGAGAACAGCAAGAAGGGCAATAGGACCCCTTACCAGTACCTGAGCAGCAACGA
GTCCAAGATCTCTTACAACCAGTTCAAGCAGCACATTCTGAACCTGTCTAAGGCCAAAGA
TAGAATCAGCAAGAAGAAACGAGATATGCTGCTGGAAGAACGGGACATCAACAAATTCGA
GGTGCAGAAGGAATTCATCAACAGAAACCTTGTGGACACCCGGTACGCCACTCGGGAGC
TGAGCAACCTGCTGAAGACCTACTTCAGCACACACGACTACGCCGTGAAAGTGAAGACC
ATCAACGGCGGCTTCACAAACCACCTGAGGAAGGTGTGGGACTTCAAGAAGCACCGGA
ACCACGGCTACAAGCACCACGCCGAGGATGCCCTGGTCATCGCCAACGCCGACTTTCT
GTTCAAAACCCACAAGGCCCTGAGACGGACAGACAAGATCCTGGAACAGCCTGGACTG
GAAGTCAACGACACCACCGTGAAGGTGGACACAGAGGAGAAGTACCAGGAGTTATTCGA
GACACCGAAACAAGTGAAGAACATCAAGCAGTTTAGAGATTTCAAGTATTCTCACAGAGT
TGACAAGAAGCCCAACCGGCAGCTGATCAATGATACCCTGTACAGTACCAGAGAGATCG
ATGGCGAAACCTACGTGGTCCAAACACTGAAAGACCTGTACGCCAAGGACAATGAAAAG
GTGAAAAAGCTCTTTACAGAACGGCCTCAAAAGATACTGATGTACCAGCACGATCCTAAG
ACCTTTGAGAAACTGATGACCATTCTGAATCAGTACGCTGAGGCAAAGAATCCTCTGGCC
GCTTATTACGAGGATAAGGGCGAATACGTGACCAAGTACGCCAAGAAGGGCAACGGCCC
TGCCATCCACAAGATCAAATACATCGACAAGAAACTGGGCAGCTACCTGGACGTGAGTAA
CAAATATCCTGAGACACAGAACAAGCTGGTGAAACTGTCTCTGAAGAGCTTTAGATTCGA
CATCTACAAATGTGAACAGGGCTACAAGATGGTGTCCATTGGCTACCTCGACGTACTGAA
GAAGGACAACTACTACTACATCCCCAAAGATAAGTACGAGGCCGAGAAGCAGAAAAAGAA
GATCAAGGAAAGCGACCTCTTCGTGGGCAGCTTCTACTACAACGACCTGATCATGTACGA
GGACGAACTCTTCCGGGTGATCGGAGTGAACTCCGATATCAACAACCTGGTTGAGCTGA
ATATGGTCGACATCACCTACAAGGATTTCTGCGAGGTGAACAACGTGACAGGCGAGAAG
AGAATCAAGAAAACCATCGGAAAGAGAGTGGTGCTGATCGAGAAGTATACGACCGACATC
CTGGGAAATCTGTATAAAACGCCCCTGCCTAAGAAGCCCCAGCTCATTTTCAAGAGAGGC
GAGCTGTCTGGCGGCTCAAAAAGAACCGCCGACGGCAGCGAATTCGAGCCCAAGAAGA
AGAGGAAAGTCTAATAGATCTCGACTGTGCCTTCTAGTTGCCAGCCATCTGTTGTTTGCC
CCTCCCCCGTGCCTTCCTTGACCCTGGAAGGTGCCACTCCCACTGTCCTTTCCTAATAAA
ATGAGGAAATTGCATCGCATTGTCTGAGTAGGTGTCATTCTATTCTGGGGGGTGGGGTGG
GGCAGGACAGCAAGGGGGAGGATTGGGAAGACAATAGCAGGCATGCTGGGGATGCGGT
GGGCTCTATGGCTCGAGCGGCCCAAGCTTAAAAAAATCTCGCCAACAAGTTGACGAGAT
AAACACGGCATTTTGCCTTGTTATAGTAGATTCTGTAATTTTCATTACAGAGTACTATAAC
CAGCCAAGGTTCGCCATAATGCGGTGTTTCGTCCTTTCCACAAGATATATAAAGCCAAGAA
ATCGAAATACTTTCAAGTTACGGTAAGCATATGATAGTCCATTTTAAAACATAATTTTAAAAC
TGCAAACTACCCAAGAAATTATTACTTTCTACGTCACGTATTTTGTACTAATATCTTTGTGTT
TACAGTCAAATTAATTCTAATTATCTCTCTAACAGCCTTGTATCGTATATGCAAATATGAAGG
AATCATGGGAAATAGGCCCTCTTCCTGCCCGACCTTGCGGCCGCAGGAACCCCTAGTGA
TGGAGTTGGCCACTCCCTCTCTGCGCGCTCGCTCGCTCACTGAGGCCGGGCGACCAAA
GGTCGCCCGACGCCCGGGCTTTGCCCGGGCGGCCTCAGTGAGCGAGCGAGCGCGCA
G
