# Supplementary material for: In vivo base editing extends lifespan of a humanized mouse model of prion disease
Source: Nat Med. 2025 Jan 14;31(4):1319–28. doi: 10.1038/s41591-024-03466-w (PMC12003183; doi:10.1038/s41591-024-03466-w)
Supplement: Supplementary file 2 — Reporting Summary [file 41591_2024_3466_MOESM2_ESM.pdf]

nature portfolio

Corresponding author(s): David R. Liu, Sonia M. Vallabh, Eric Vallabh Minikel

Last updated by author(s): Nov 26, 2024

Reporting Summary

Nature Portfolio wishes to improve the reproducibility of the work that we publish. This form provides structure for consistency and transparency in reporting. For further information on Nature Portfolio policies, see our Editorial Policies and the Editorial Policy Checklist.

Statistics

For all statistical analyses, confirm that the following items are present in the figure legend, table legend, main text, or Methods section.

n/a	Confirmed	
☐	☒	The exact sample size (*n*) for each experimental group/condition, given as a discrete number and unit of measurement
☐	☒	A statement on whether measurements were taken from distinct samples or whether the same sample was measured repeatedly
☐	☒	The statistical test(s) used AND whether they are one- or two-sided<br>*Only common tests should be described solely by name; describe more complex techniques in the Methods section.*
☒	☐	A description of all covariates tested
☒	☐	A description of any assumptions or corrections, such as tests of normality and adjustment for multiple comparisons
☐	☒	A full description of the statistical parameters including central tendency (e.g. means) or other basic estimates (e.g. regression coefficient) AND variation (e.g. standard deviation) or associated estimates of uncertainty (e.g. confidence intervals)
☐	☒	For null hypothesis testing, the test statistic (e.g. *F*, *t*, *r*) with confidence intervals, effect sizes, degrees of freedom and *P* value noted<br>*Give P values as exact values whenever suitable.*
☒	☐	For Bayesian analysis, information on the choice of priors and Markov chain Monte Carlo settings
☒	☐	For hierarchical and complex designs, identification of the appropriate level for tests and full reporting of outcomes
☐	☒	Estimates of effect sizes (e.g. Cohen's *d*, Pearson's *r*), indicating how they were calculated

Our web collection on statistics for biologists contains articles on many of the points above.

Software and code

Policy information about availability of computer code

Data collection	Miseq reporter software (v2.6) was used on the Illumina Miseq to demultiplex HTS data. Beckman CytoFLEX LX was used for flow cytometry with CytExpert Acquisition and Analysis Software (v2.4). . Fluorescence activated cell sorting (FACS) was performed on the SONY MA900 Cell Sorter (Sony Biotechnology) with MA900 Cell Sorter software (v3.1). QX ONE ddPCR system was used for ddPCR with QX Manager Software, Standard Edition (v1.4.0). IDT online rhAmpSeq design tool (www.idtdna.com/rhAmpSeqDesignTool) (no version number available) was used for the design of rhAmpSeq primer pools for the targeted amplicon sequencing of nominated off-target sites.
Data analysis	Mean, 95% confidence interval, standard error of mean, linear correlation were calculated using GraphPad Prism 10. CRISPResso2 (v2.2.12) was used to analyze HTS data for quantifying editing efficiency at the genomic sites (https://github.com/pinellolab/CRISPResso2). CIRCLE-seq analysis was performed using open-source CIRCLE-seq analysis software (v1.1) and default recommended parameters (https://github.com/tsailabSJ/circleseq). Code used to analyze the frequency of off-target editing are deposited to Github (https://github.com/ericminikel/base_editing). Flow cytometry data were analyzed by FlowJo (v10.10). Sequences for rhAmpSeq amplicons were extracted using the R Bioconductor BSGenome package (v1.4.3) using the GRCm38/mm10 (mouse) and GRCh37/hg19 (human) reference genomes.

For manuscripts utilizing custom algorithms or software that are central to the research but not yet described in published literature, software must be made available to editors and reviewers. We strongly encourage code deposition in a community repository (e.g. GitHub). See the Nature Portfolio guidelines for submitting code & software for further information.

Data

Policy information about availability of data

All manuscripts must include a data availability statement. This statement should provide the following information, where applicable:

- Accession codes, unique identifiers, or web links for publicly available datasets
- A description of any restrictions on data availability
- For clinical datasets or third party data, please ensure that the statement adheres to our policy

There is no restriction on experimental data availability from this study. High-throughput DNA sequencing data files are deposited to the National Center for Biotechnology Information's Sequence Read Archive (NCBI SRA) database under accession code PRJNA1178796. DNA sequences of the AAV vectors are provided in the Supplementary Sequences. GRCm38/mm10 (mouse) reference genome sequence was obtained from NCBI RefSeq assembly GCF_000001635.20. GRCh37/hg19 (human) reference genome sequence was obtained from NCBI RefSeq assembly GCF_000001405.13. Other raw data are deposited in the study's online git repository at https://github.com/ericminikel/base_editing.

Research involving human participants, their data, or biological material

Policy information about studies with human participants or human data. See also policy information about sex, gender (identity/presentation), and sexual orientation and race, ethnicity and racism.

Reporting on sex and gender	Not applicable - the use of human pathogenic prion isolate is ruled as "Not Human Subjects Research" (Broad ORSP NHSR-5934)
Reporting on race, ethnicity, or other socially relevant groupings	Not applicable.
Population characteristics	No data on population characteristics were collected.
Recruitment	This study does not involve human participants, therefore recruitment is not applicable to the study.
Ethics oversight	The use of human pathogenic prion isolate is ruled as "Not Human Subjects Research" (Broad ORSP NHSR-5934). Use of human tissue was approved under NPDPSC IRB protocol 01-14-18 and Safar lab IRB protocol 03-14-28.

Note that full information on the approval of the study protocol must also be provided in the manuscript.

Field-specific reporting

Please select the one below that is the best fit for your research. If you are not sure, read the appropriate sections before making your selection.

☒ Life sciences ☐ Behavioural & social sciences ☐ Ecological, evolutionary & environmental sciences

For a reference copy of the document with all sections, see nature.com/documents/nr-reporting-summary-flat.pdf

Life sciences study design

All studies must disclose on these points even when the disclosure is negative.

Sample size	Sample sizes were n=3 independent biological replicates for cell culture experiments, in accordance with previous literature and standards in the field of genome editing technologies (Anzalone 2019, Levy 2020, Banskota and Raguram 2022). Sample sizes for in vivo experiments are specified in the figure legends. No statistical method was used to predetermine sample size but our sample sizes are similar to those reported in previous publications (Neumann and Bertozzi 2024, Reichart, Newby and Wakimoto, 2023).
Data exclusions	Mice that met predetermined criteria of euthanasia were excluded from the study. The predetermined criteria include: signs of pain or distress (including lethargy, significant hair loss, loss of body weight of 20% or more from pre-injection baseline), respiratory distress, neurological deficits, dehydration, inability to prehend food or access water.
Replication	All experiments were conducted for at least three times with biological replicates and all attempts at replication were successful.
Randomization	All control and test conditions were assigned randomly. For in vivo experiments, mice were randomly assigned into groups, with consideration on including both sexes in each experimental group. For cell culture experiments, conditions were assigned randomly to wells across 96-well plates.
Blinding	All HTS data were analyzed using an automated CRISPResso2 script that does not allow experimenter intervention, therefore experimenter was not blinded. Human pathogenic prion isolate inoculation challenge study data analysis (HTS, ELISA, lifespan) were performed by blinded investigators.

Reporting for specific materials, systems and methods

We require information from authors about some types of materials, experimental systems and methods used in many studies. Here, indicate whether each material, system or method listed is relevant to your study. If you are not sure if a list item applies to your research, read the appropriate section before selecting a response.

Materials & experimental systems

n/a	Involved in the study
☐	☒ Antibodies
☐	☒ Eukaryotic cell lines
☒	☐ Palaeontology and archaeology
☐	☒ Animals and other organisms
☒	☐ Clinical data
☒	☐ Dual use research of concern
☒	☐ Plants

Methods

n/a	Involved in the study
☒	☐ ChIP-seq
☐	☒ Flow cytometry
☒	☐ MRI-based neuroimaging

Antibodies

Antibodies used

Mouse anti-230 antibody 6D11 diluted 1:100 (BioLegend, 808008); Rabbit anti-prion antibody EP1802Y (Abcam, ab52604) and mouse anti-prion antibody 8H4 (Abcam, ab61409) are diluted to 2 µg/ml for PrP ELISA.

Validation

All commercial antibodies were validated by the manufacturers. For mouse anti-230 antibody 6D11, manufacturer states "This antibody is effective in immunoblotting (WB), immunohistochemistry (IHC), ELISA, immunoprecipitation (IP), and flow cytometry (FC). 6D11 reacts with both the PrPc and PrPsc forms. The epitope falls within amino acids 93-109 of PrP". For rabbit anti-prion antibody EP1802Y, manufacturer states "We have tested this species and application combination and it works.". For mouse anti-prion antibody 8H4 (Abcam, ab61409), manufacturer states "We have not tested this specific species and application combination in-house, but expect it will work". The use of rabbit anti-prion antibody EP1802Y and mouse anti-prion antibody 8H4 for the assessment of PrP level via ELISA has been previously described in previous publication (Nuvolone 2016).

Eukaryotic cell lines

Policy information about cell lines and Sex and Gender in Research

Cell line source(s)

HEK293T cells (ATCC, CRL-3216); HEK293T clone 17 cells (ATCC, CRL-11268)

Authentication

Commercial cell lines were authenticated by the supplier using STR analysis.

Mycoplasma contamination

All cell lines were tested negative for mycoplasma.

Commonly misidentified lines
(See ICLAC register)

Not used.

Animals and other research organisms

Policy information about studies involving animals; ARRIVE guidelines recommended for reporting animal research, and Sex and Gender in Research

Laboratory animals

Tg666 humanized mouse line was a generous gift from the Research Foundation for Mental Hygiene facilitated by NIH Rocky Mountain Laboratories. Tg666 were treated with AAVs at 4-weeks of age. Tg25109 contains 3 copies of a human 129M PRNP BAC on a background of endogenous Prnp knockout (ZH3/ZH3) on a mixed C57BL6N/J background, and are provided by Prion Alliance. Tg25109 mice were treated at 5-8-weeks of age. Mouse housing facilities were maintained at 20-22°C with 30-50% humidity. Mice were kept on a 12 h light/dark cycle with ad libitum access to standard rodent diet and water.

Wild animals

This study does not involve wild animals.

Reporting on sex

Both sexes were included for each experimental condition in in vivo experiments involving mouse models. Both sexes were assigned to each experimental group as evenly as possible, availability of mice permitting. Sex disaggregated numbers for individual experiments are provided in Supplementary Table 8. No sex-based analysis is performed as prion disease is not a sex-linked disease therefore it is not expected to have sex-based differences. Also due to the limited availability of the mice, we did not include sufficient sample size for each sex to enable sex-based analysis.

Field-collected samples

This study does not involve samples collected from the field.

Ethics oversight

Broad Institute IACUC committee (D16-00903; 0162-05-16-2 and 0048-04-15-2) provided ethics overnight for all experiments involving live animals.

Note that full information on the approval of the study protocol must also be provided in the manuscript.

Plants

Seed stocks	*Report on the source of all seed stocks or other plant material used. If applicable, state the seed stock centre and catalogue number. If plant specimens were collected from the field, describe the collection location, date and sampling procedures.*
Novel plant genotypes	*Describe the methods by which all novel plant genotypes were produced. This includes those generated by transgenic approaches, gene editing, chemical/radiation-based mutagenesis and hybridization. For transgenic lines, describe the transformation method, the number of independent lines analyzed and the generation upon which experiments were performed. For gene-edited lines, describe the editor used, the endogenous sequence targeted for editing, the targeting guide RNA sequence (if applicable) and how the editor was applied.*
Authentication	*Describe any authentication procedures for each seed stock used or novel genotype generated. Describe any experiments used to assess the effect of a mutation and, where applicable, how potential secondary effects (e.g. second site T-DNA insertions, mosiacism, off-target gene editing) were examined.*

Flow Cytometry

Plots

Confirm that:

☒ The axis labels state the marker and fluorochrome used (e.g. CD4-FITC).

☒ The axis scales are clearly visible. Include numbers along axes only for bottom left plot of group (a 'group' is an analysis of identical markers).

☒ All plots are contour plots with outliers or pseudocolor plots.

☒ A numerical value for number of cells or percentage (with statistics) is provided.

Methodology

Sample preparation	HEK293T cells that were treated by plasmid transfection were washed with flow cytometry buffer six-days after treatment, and were incubated with anti-CD230 6D11 antibodies for 30 min on ice in the dark. Cells were washed twice with the flow cytometry buffer after antibody incubation.
Instrument	CytoFLEX LX Flow Cytometer (Beckman Coulter, C06779) was used for flow cytometry.
Software	CytExpert Acquisition and analysis Software (v2.4) was used for data acquisition. FlowJo (v10.10) was used for the data analysis.
Cell population abundance	The mean fluorescence intensity reduced by 43% after BE4max treatment, 53% after Cas9 nuclease treatment and no reduction after dead BE4max treatment.
Gating strategy	Single cells were gated by FSC-H:SSC-H ratio and FSC-A:FSC-H ratio. PrP-negative population was gated with untreated sample as a benchmark.

☒ Tick this box to confirm that a figure exemplifying the gating strategy is provided in the Supplementary Information.
